# Ligand-controlled cobalt-catalyzed regiodivergent hydroboration of aryl,alkyl-disubstituted internal allenes†

**DOI:** 10.1039/c9sc06136c

**Published:** 2020-02-05

**Authors:** Caizhi Wu, Shaozhong Ge

**Affiliations:** Department of Chemistry, National University of Singapore 3 Science Drive 3 Singapore 117543 Singapore chmgsh@nus.edu.sg

## Abstract

We report a stereoselective regiodivergent hydroboration of aryl,alkyl-disubstituted internal allenes with pinacolborane (HBpin) in the presence of cobalt catalysts generated from bench-stable Co(acac)_2_ and bisphosphine ligands. An interesting correlation between the regioselectivity of this hydroboration and the bite angles of bisphosphine ligands was identified. When hydroboration was conducted with cobalt catalysts containing bisphosphines with medium bite angles (*e.g.* 98° for dppb and 96° for dppf), HBpin was selectively added to the alkyl-substituted double bond. However, HBpin was selectively added to the aryl-substituted double bond when the reactions were conducted with cobalt catalysts containing bisphosphines with large bite angles (*e.g.* 111° for xantphos and 114° for Nixantphos). A range of internal allenes underwent these Co-catalyzed hydroboration reactions in a regiodivergent manner to yield the corresponding (*Z*)-alkenylboronates in high isolated yields and with high regioselectivity. These reactions show good functional group compatibility and can be readily scaled up to gram scales without using a dry box. In addition, the comparison of regioselectivity between the Co-catalyzed hydrosilylation and hydroboration reactions of the same allene substrate suggests that this Co-catalyzed regiodivergent hydroboration of allenes proceeds through a Co-Bpin intermediate. Deuterium-labeling experiments suggest that the Co-Bpin intermediates react with allenes to form allylcobalt species which then react with HBpin to release (*Z*)-alkenylboronate products.

## Abstract 2

The first cobalt-catalyzed stereoselective regiodivergent hydroboration of internal allenes to access synthetically versatile (*Z*)-alkenylboronate compounds was effectively achieved with two cobalt catalysts through ligand control.

## Graphical abstract

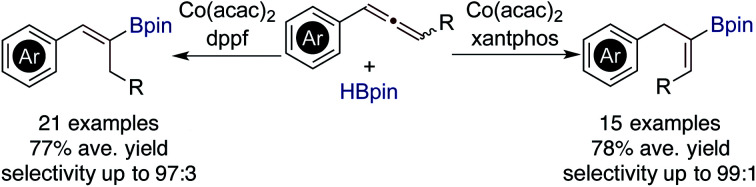

## Introduction

Alkenylboronates are synthetically versatile building blocks in organic chemistry because they are stable and non-toxic, and can undergo a range of catalytic cross-coupling reactions to produce stereodefined multisubstituted alkenes.^1^ The hydroboration of allenes with hydroboranes may be an effective and atom-economical approach to prepare these alkenylboronates. However, the development of selective hydroboration of allenes with hydroboranes to access alkenylboron compounds is very challenging because this reaction can potentially yield a series of organoboron products caused by diverse regio- and stereoselectivity issues. For example, hydroboranes can add to either of the two double bonds of allenes, the stereochemistry of the remaining double bond can be *cis* or *trans*, and both monohydroboration and dihydroboration can take place.

**Scheme 1:**
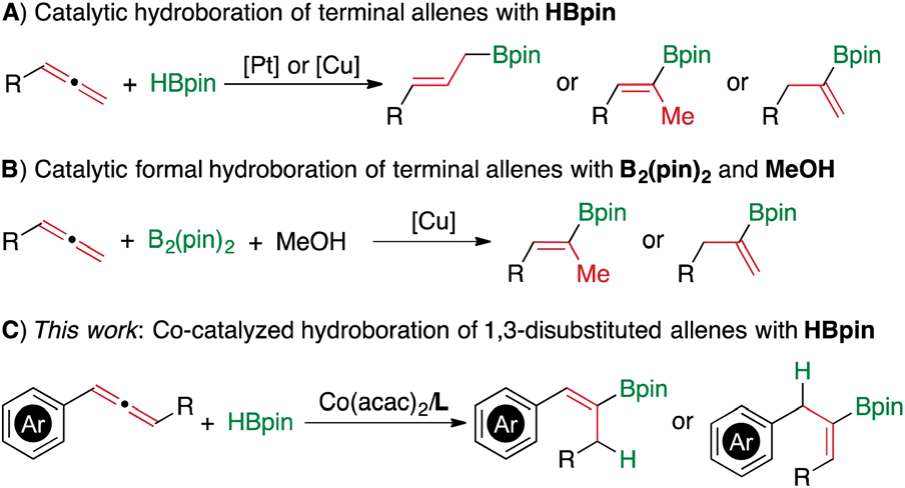
Hydroboration and formal hydroboration of allenes.

Allenes can readily undergo uncatalyzed hydroboration with reactive dialkylboranes to afford allylboron or alkenylboron products.^2^ The regio- and stereo-selectivity for these uncatalyzed hydroboration reactions are generally controlled by allene or dialkylborane reagents. However, the hydroboration of allenes with less reactive dialkoxyboranes, such as pinacolborane (HBpin), does not occur in the absence of metal catalysts. Accordingly, the hydroboration of allenes with HBpin has been studied with platinum and copper catalysts,^3^ and these reactions have been limited to terminal allenes with a single example employing an internal allene (Scheme 1A).^3*b*^ Alternatively, formal hydroboration of terminal allenes with B_2_(pin)_2_ in the presence of MeOH as a proton source has been achieved with copper catalysts (Scheme 1B).^3*b*,4^ To the best of our knowledge, metal catalysts for the hydroboration of 1,3-disubstituted allenes with HBpin to yield alkenylboronates still remain unknown. Driven by our interest in developing cobalt catalysts for selective hydrofunctionalization of unsaturated hydrocarbons,^5^ we are interested in identifying selective cobalt catalysts for the hydroboration of internal allenes to prepare (*Z*)-alkenylboronate compounds (Scheme 1C).

In recent years, cobalt complexes have gained increasing attention in hydroboration^6^ and hydrosilylation^7^ of unsaturated hydrocarbons,^8^ and the majority of these reactions occur through cobalt hydride intermediates.^9^ Very recently, we have reported Co-catalyzed diborylation of alkenes and mechanistic studies suggest the existence of a Co-Bpin species.^10^ To develop Co-catalyzed hydroboration of internal allenes, we envisioned that the reaction would proceed through borylmetallation to afford alkenylboronates. Herein, we report Co-catalyzed regiodivergent hydroboration of aryl,alkyl-disubstituted internal allenes to prepare (*Z*)-alkenylboronates, and the regioselectivity of this Co-catalyzed hydroboration is predominantly controlled by bisphosphine ligands employed in these reactions.

## Results and discussion

We initiated the studies on the Co-catalyzed hydroboration of allenes by identifying selective and reactive cobalt catalysts for the reaction between buta-1,2-dieny-1-ylbenzene (**1a**) and HBpin. The catalysts generated *in situ* from Co(acac)_2_ and a series of phosphine ligands were tested and the results are summarized in Table 1. In general, these reactions were conducted with allene **1a** as a limiting reagent in the presence of 1.1 equivalents of HBpin and 3 mol% catalysts at room temperature for 12 h. This hydroboration reaction yielded two (*Z*)-alkenylboronate products **2a** and **3a**, resulting from the addition of HBpin to the alkyl-substituted and aryl-substituted alkene moieties, respectively.

**Table 1:** Evaluation of conditions for the Co-catalyzed hydroboration of allene **1a** with HBpin^a^

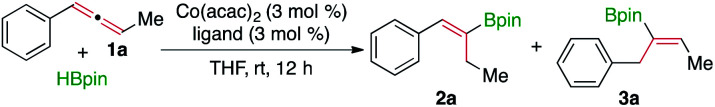					
Entry	Ligand	Bite angle^b^	Conversion^c^ (%)	Ratio^c^ (**2a**/**3a**)	Yield (%)
1	PMe_3_^d^	—	>99	71 : 29	—
2	PPh_3_^d^	—	>99	83 : 17	—
3	Dppm	72	>99	65 : 35	—
4	Dppe	85	18	76 : 24	—
5	Dppp	91	15	86 : 14	—
6	Dppb	98	>99	94 : 6	78 (**2a**)
7	Dpppe	—	>99	83 : 17	—
8	Dppbz	83	>99	74 : 26	—
9	*rac*-binap	92	91	84 : 16	—
10	Dppf	96	>99	96 : 4	81 (**2a**)
11	dpephos	102	>99	61 : 39	—
12	Xantphos	111	>99	6 : 94	80 (**3a**)
13	Nixantphos	114	>99	8 : 92	76 (**3a**)
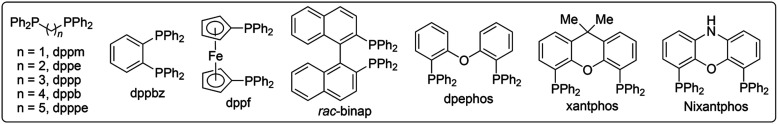					

a Conditions: buta-1,2-dien-1-ylbenzene (0.400 mmol), HBpin (0.440 mmol), Co(acac)_2_ (12 μmol), ligand (12 μmol), toluene (1 mL), room temperature, and 12 h.

b These values are taken from ref. 11, and the bite angle of dpppe has not been reported.

c Determined by GC analysis with dodecane as the internal standard.

d 6 mol% of ligand was used.

The reactions conducted with monophosphine ligands, such as PMe_3_ and PPh_3_, proceeded to complete conversion of **1a**, affording a mixture of **2a** and **3a** with a modest selectivity towards **2a** (entries 1 and 2). The catalysts generated by the combination of Co(acac)_2_ and a series of bisphosphine ligands were subsequently evaluated, and the majority of these cobalt catalysts (entries 3 and 6–13) were highly active for the hydroboration of **1a**, except the catalysts containing dppe and dppp ligands (entries 4 and 5). These reactions showed varied selectivity for products **2a** and **3a**. In addition, we also tested other hydroboranes, such as HB(cat) and 9-BBN, for this Co-catalyzed hydroboration of **1a**. However, the reactions with these hydroboranes became less selective and multiple products were obtained (see the ESI† for details).

**Fig. 1:**
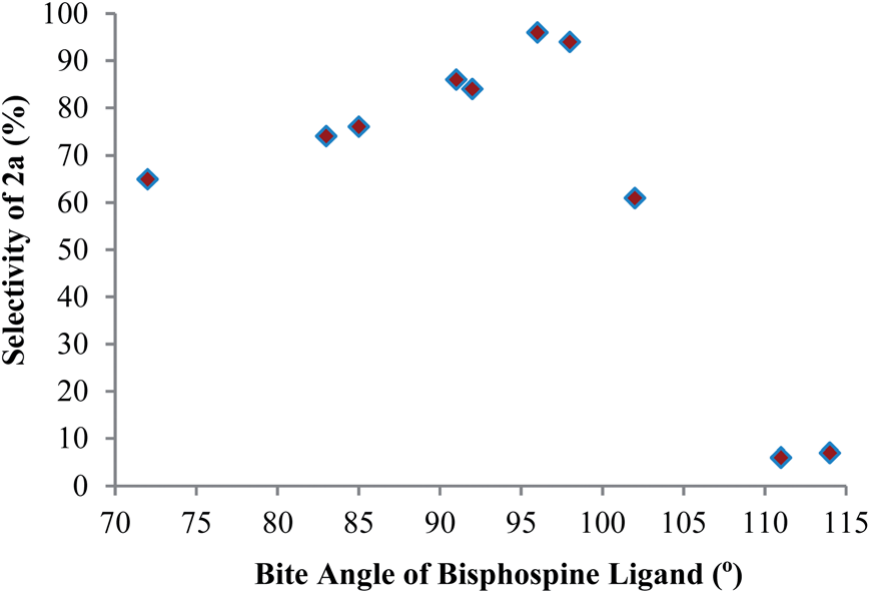
Plotting of the selectivity of **2a***vs.* bite angles of ligands.

While studying the ligand effect on the Co-catalyzed hydroboration of allene **1a**, we found that the selectivity of this reaction was sensitive to the bite angles of the bisphosphine ligands,^11^ and the correlation between the selectivity towards product **2a** and the bite angles of bisphosphines is depicted in Fig. 1. The selectivity of product **2a** increased with the increase of bite angles till the highest selectivity was reached for dppf and dppb ligands with bite angles of 96° and 98°, respectively (entries 6 and 10). However, in the presence of ligands with large bite angles, such as xantphos and Nixantphos, the hydroboration of **1a** afforded the (*Z*)-alkenylboronate **3a** as the major product in high isolated yields with high selectivity (entries 12 and 13).

The two internal double bonds in allene **1a** have different steric properties and the phenyl-substituted double bond is more sterically hindered than the methyl-substituted one, which means that the methyl-substituted double bond is sterically more accessible for hydroboration. Meanwhile, the phenyl-substituted double bond is more reactive towards hydroboration due to the electron-withdrawing effect of the phenyl group. Therefore, the observed ligand effect (Fig. 1) on the regioselectivity of this Co-catalyzed hydroboration of allene **1a** could be explained by the steric and electronic interactions between the substrate **1a** and the cobalt catalysts. The data in Fig. 1 suggest that steric effects dominate with bisphosphine ligands with medium bite angles (such as dppb and dppf) and that electronic activation dominates with bisphosphine ligands with large bite angles (such as xantphos and Nixantphos).

**Fig. 2:**
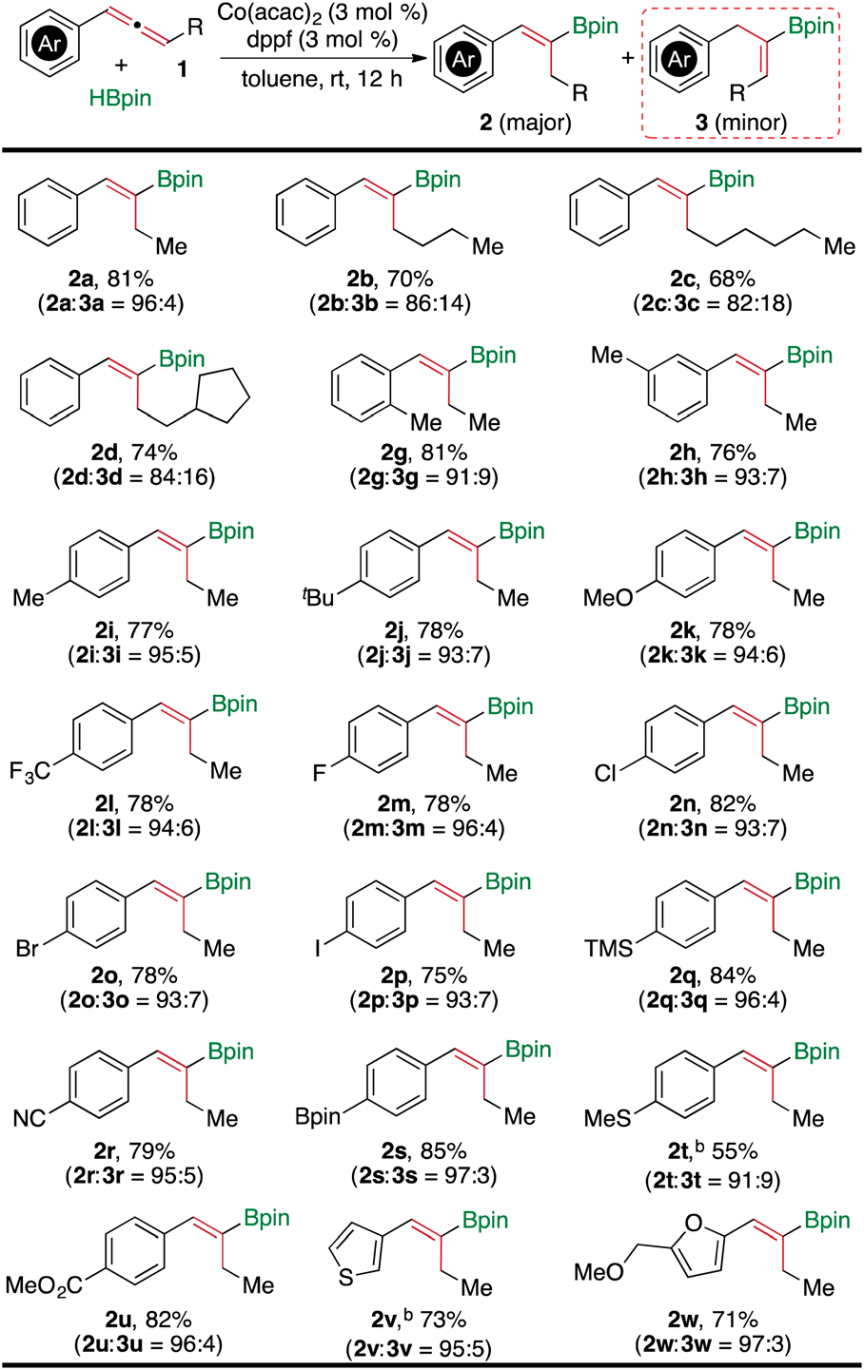
Co(acac)_2_/dppf-catalyzed hydroboration of internal allenes. ^a^Conditions: allene (0.400 mmol), HBpin (0.440 mmol), Co(acac)_2_ (12 μmol), dppf (12 μmol), toluene (1 mL), room temperature, 12 h, and isolated yields after column chromatography; ^b^18 h.

Under the identified conditions (entry 10 in Table 1), we studied the scope of internal allenes that undergo this cobalt-catalyzed hydroboration to yield the alkenylboronate **2** and the results are summarized in Fig. 2. In general, a wide range of internal allenes reacted smoothly with HBpin in the presence of 3 mol% Co(acac)_2_/dppf at room temperature, affording the corresponding (*Z*)-alkenylboronates (**2a–2u**) in high yields (55–85%) with high regioselectivity (**2** : **3** ratio up to 97 : 3). The GC-MS analysis on crude mixtures of these reactions showed that the formation of trace amounts (<5%) of the corresponding alkene products resulted from the hydrogenation of allene substrates.

The data in Fig. 2 indicate that the electronic properties of aromatic substituents of the allenes have little influence on the yield and regioselectivity of these Co-catalyzed hydroboration reactions that produce the alkenylboronate **2**. For example, allenes containing electron-rich (**2k**, **2v**, and **2w**), electron-neutral (**2a** and **2m**), and electron-deficient (**2l**, **2r**, and **2u**) aryl groups reacted with similarly high regioselectivity, and the isolated yields of the corresponding (*Z*)-alkenylboronates are similarly high as well. This is likely because the hydroboration does not take place on the aryl-substituted double bond. As HBpin was added to the alkyl-substituted double bond, the steric properties of the aliphatic groups have a noticeable effect on the regioselectivity. For example, the regioselectivity towards the alkenylboronate **2** decreased when the aliphatic groups became more bulky (**2a–2d**). Most significantly, hydroboration of the alkyl-substituted double bond was inhibited when the alkyl substituents of allenes were changed to cyclohexyl and isopropyl groups (**1e** and **1f**). Instead, the hydroboration of these two allenes yielded the alkenylboronates **3e** and **3f** in high yields in the presence of Co(acac)_2_ and dppf (eqn (1)). The results of these two reactions (eqn (1)) suggest that this Co/dppf-catalyzed hydroboration reaction occurs at the phenyl-activated double bond when both double bonds in allenes become similarly accessible with respect to steric hindrance.1
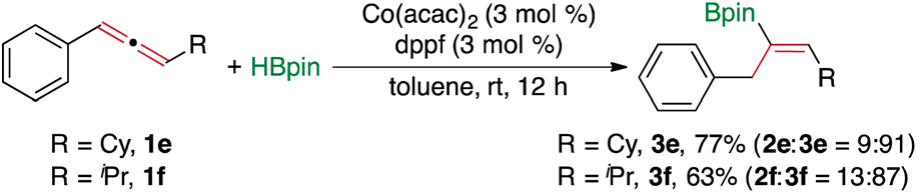


This Co(acac)_2_/dppf-catalyzed hydroboration of allenes tolerates a range of functionalities, including ether (**2k**), chloro (**2n**), bromo (**2o**), iodo (**2p**), trimethylsilyl (**2q**), cyano (**2r**), boronic ester (**2s**), thioether (**2t**), and carboxylic ester (**2u**) moieties. In addition, sulfur- and oxygen-containing heteroaryl substituted allenes also reacted with high yields and high regioselectivity (**2v** and **2w**).

**Fig. 3:**
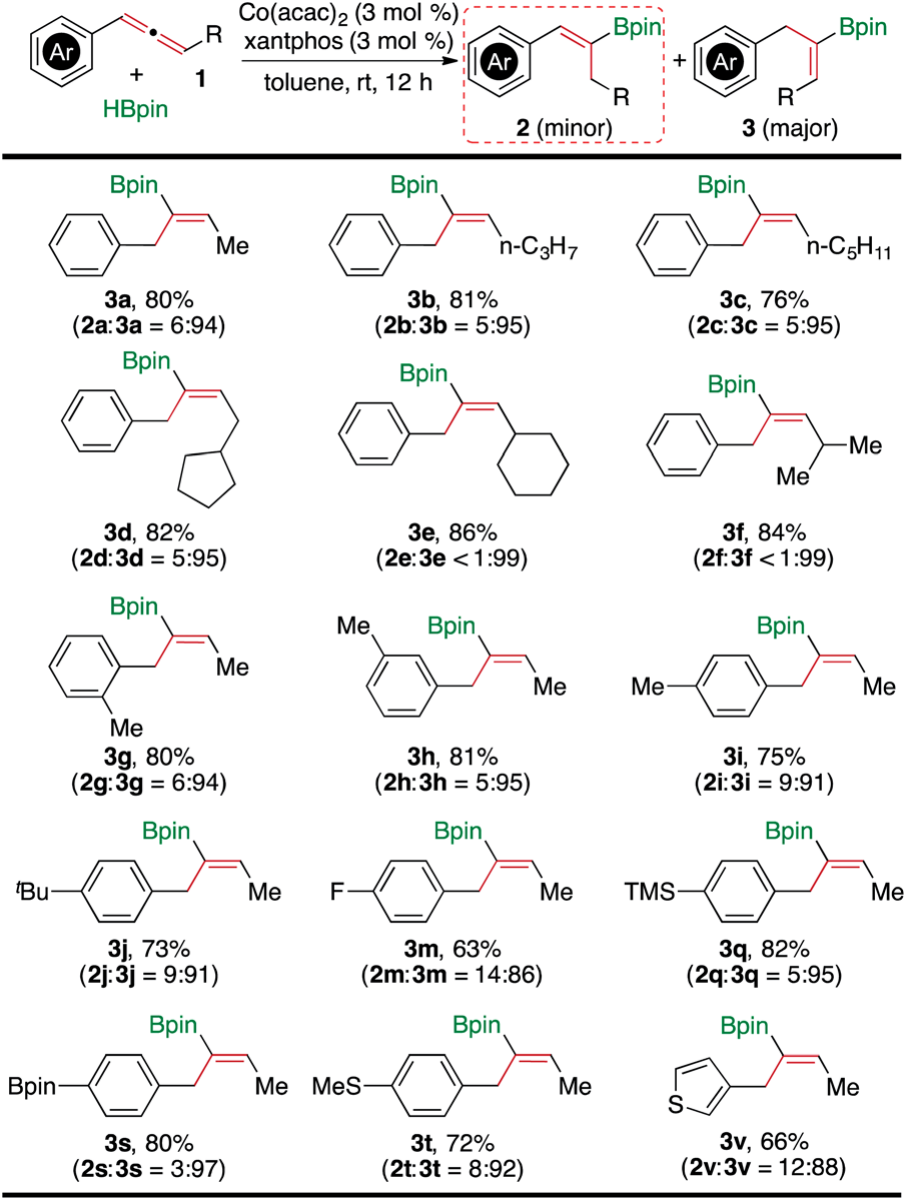
Co(acac)_2_/xantphos-catalyzed hydroboration of internal allenes. ^a^Conditions: allene (0.400 mmol), HBpin (0.440 mmol), Co(acac)_2_ (12 μmol), xantphos (12 μmol), toluene (1 mL), room temperature, 12 h, and isolated yields after column chromatography.

Fig. 3 shows the summary of the scope of allenes that undergo hydroboration to yield the alkenylboronate **3** in the presence of Co(acac)_2_ and xantphos (entry 12 in Table 1). In general, a range of internal allenes underwent this Co-catalyzed hydroboration in the presence of 3 mol% Co(acac)_2_/xantphos at room temperature, yielding the corresponding (*Z*)-alkenylboronates in high yields (63–86%) with good regioselectivity (**2** : **3** ratio up to 1 : 99). Different from the Co(acac)_2_/dppf-catalyzed allene hydroboration, the hydroboration of allenes catalyzed by Co(acac)_2_ and xantphos could not tolerate chloro-, bromo-, and iodo-substituted aromatic groups, which underwent Co-mediated C-X (X = Cl, Br, or I) borylation under the reaction conditions.^12^ The GC-MS analysis on the crude reaction mixtures of the Co(acac)_2_/xantphos-catalyzed hydroboration of allenes **1n–1p** indicated the formation of the alkenylboronate **3s**.

**Scheme 2:**
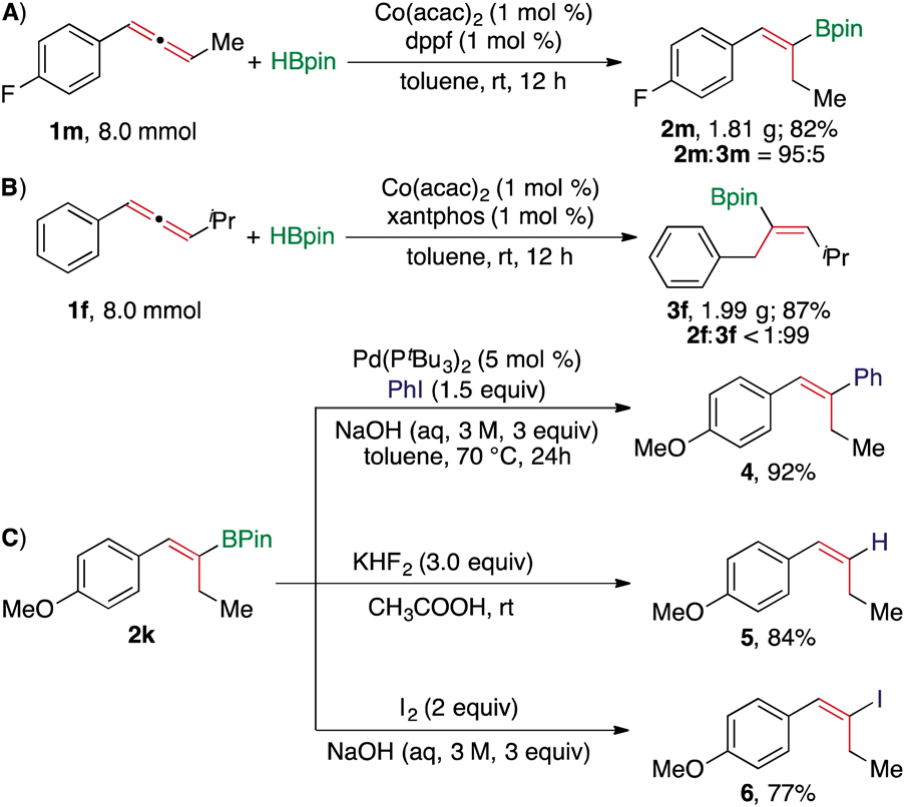
Gram-scale reactions and transformations of alkenylboronate **2k**.

**Scheme 3:**
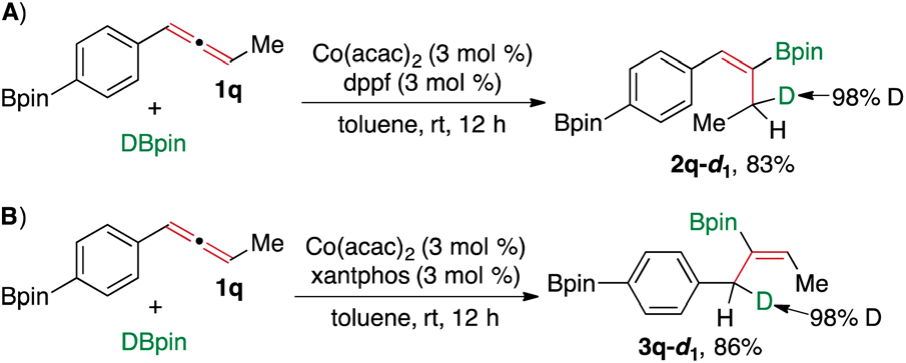
Deuterium-labelling experiments.

Subsequently, we conducted gram-scale reactions to highlight the utility of these Co-catalyzed protocols for the synthesis of (*Z*)-alkenylboronates. The hydroboration of allenes **1m** and **1f** on a 8.0 mmol scale proceeded well in the presence of 1 mol% Co(acac)_2_/dppf and Co(acac)_2_/xantphos, yielding the corresponding (*Z*)-alkenylboronate **2m** (1.81 g and 82%, Scheme 2A) and **3f** (1.99 g and 87%, Scheme 2B) with similarly high regioselectivity, respectively. In addition, we also showed that the (*Z*)-alkenylboronate products could be utilized in several organic transformations (Scheme 2C). For example, the (*Z*)-alkenylboronate **2k** could undergo Pd-catalyzed Suzuki–Miyaura coupling with iodobenzene to give a trisubstituted (*E*)-alkene **4** in 92% yield, protodeboronation to yield a (*Z*)-alkene **5** in 84% yield, and iodination with I_2_ to produce an (*E*)-alkenyl iodide **6** in 77% yield. Thus, this Co-catalyzed allene hydroboration provides a practical method to prepare organic compounds containing stereodefined alkene moieties.

To understand this Co-catalyzed hydroboration of internal allenes, we conducted deuterium-labeling experiments in the presence of both cobalt catalysts, and the results are summarized in Scheme 3. The hydroboration of buta-1,2-dien-1-ylbenzene (**1a**) with DBpin in the presence of Co(acac)_2_ and dppf produced **2a**-*d*_1_, and the deuterium atom of this alkenylboronate was exclusively located at the allylic carbon (Scheme 3A). The same reaction catalyzed by Co(acac)_2_ and xantphos yielded **3a**-*d*_1_ with the deuterium atom located at the benzylic carbon (Scheme 3B). Previous studies suggest that the hydroboration reactions of unsaturated hydrocarbons proceed through either a Co–H or a Co-Bpin intermediate. However, the results of these deuterium-labeling experiments could not provide conclusive evidence on the identity of cobalt intermediates in these allene hydroboration reactions.

**Scheme 4:**
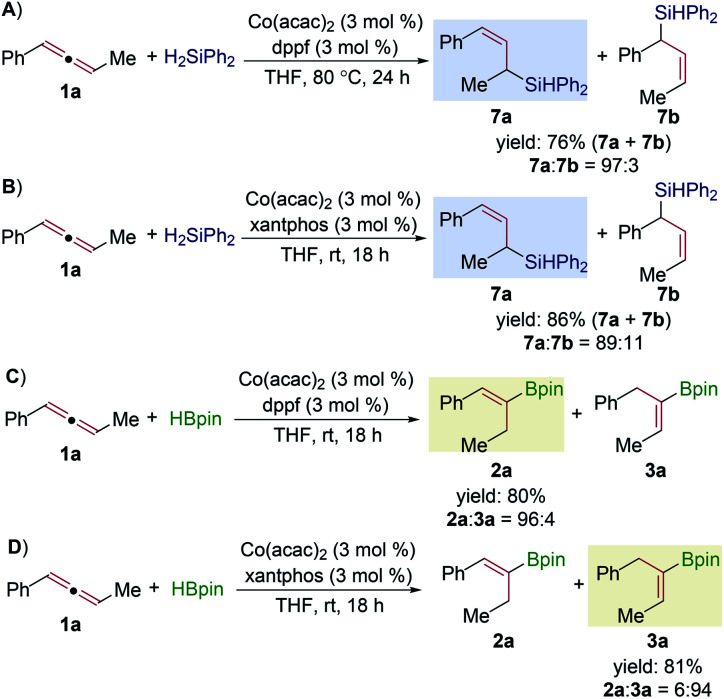
Comparison of regioselectivity in hydrosilylation and hydroboration of allene **1a**.

To reveal the cobalt intermediate in this Co-catalyzed allene hydroboration, we conducted the hydroboration and hydrosilylation reactions of the internal allene **1a** in the presence of Co(acac)_2_/dppf or Co(acac)_2_/xantphos and compared the regioselectivity of the corresponding reactions (Scheme 4). The hydrosilylation of **1a** with Ph_2_SiH_2_ conducted with both cobalt catalysts produced the allylsilanes **7a** and **7b** with high regioselectivity for **7a** (Scheme 4A and B). The results of these hydrosilylation reactions of internal allenes are consistent with our previous studies on cobalt-catalyzed hydrosilylation of terminal allenes, which occurs through a Co–H intermediate and produces allylsilanes as major products.^13^ If the hydroboration reaction of internal allenes occurs through the same Co–H intermediate as in the corresponding hydrosilylation reaction, the hydroboration reactions conducted with both cobalt catalysts will selectively yield allylboronate products. However, the hydroboration of **1a** with HBpin by both cobalt catalysts produced the alkenylboronates **2a** and **3a** as major products (Scheme 4C and D). These results suggest that this Co-catalyzed hydroboration of allenes occurs through a Co-Bpin intermediate.

**Scheme 5:**
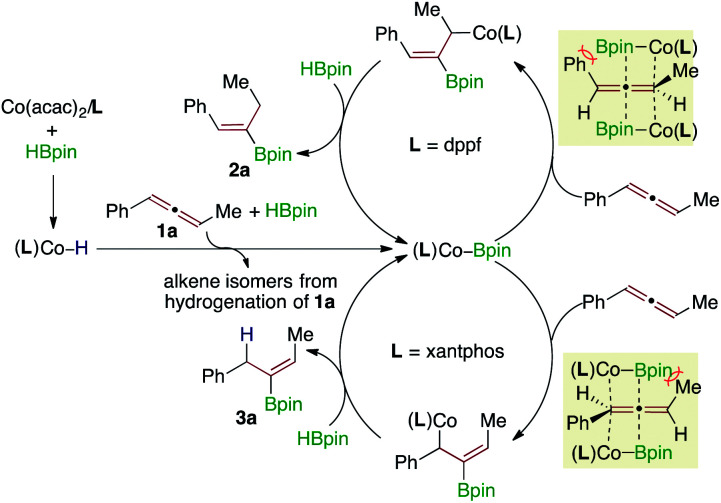
The proposed catalytic cycle for this cobalt-catalyzed regiodivergent hydroboration of aryl,alkyl-1,3-disubstituted allenes.

Based on the results of control experiments and ligand effects on the observed regioselectivity, a plausible catalytic pathway is depicted in Scheme 5 for this Co-catalyzed stereoselective regiodivergent hydrosilylation of aryl,alkyl-1,3-disubstituted allenes. The activation of Co(acac)_2_ with HBpin in the presence of a bisphsophine ligand generates a cobalt hydride species (**L**)Co–H,^5*c*^ which is then converted to a (**L**)Co-Bpin species by the reaction of the allene **1a** and HBpin with the concomitant hydrogenation of the allene.^10*a*^ Accordingly, the products from the hydrogenation of **1a** were detected by GC-MS analysis. Subsequently, the migratory insertion of allenes (for demonstration purposes, only the insertion of one enantiomer of allene **1a** is shown in Scheme 5) into this (**L**)Co-Bpin species produces the allylcobalt intermediates **I** (for the dppf ligand, steric control) or **II** (for the xantphos ligand, electronic control), which then react with HBpin to release the alkenylboronate products **2a** or **3a** and regenerate the catalytically active cobalt boryl intermediate (**L**)Co-Bpin.

**Scheme 6:**
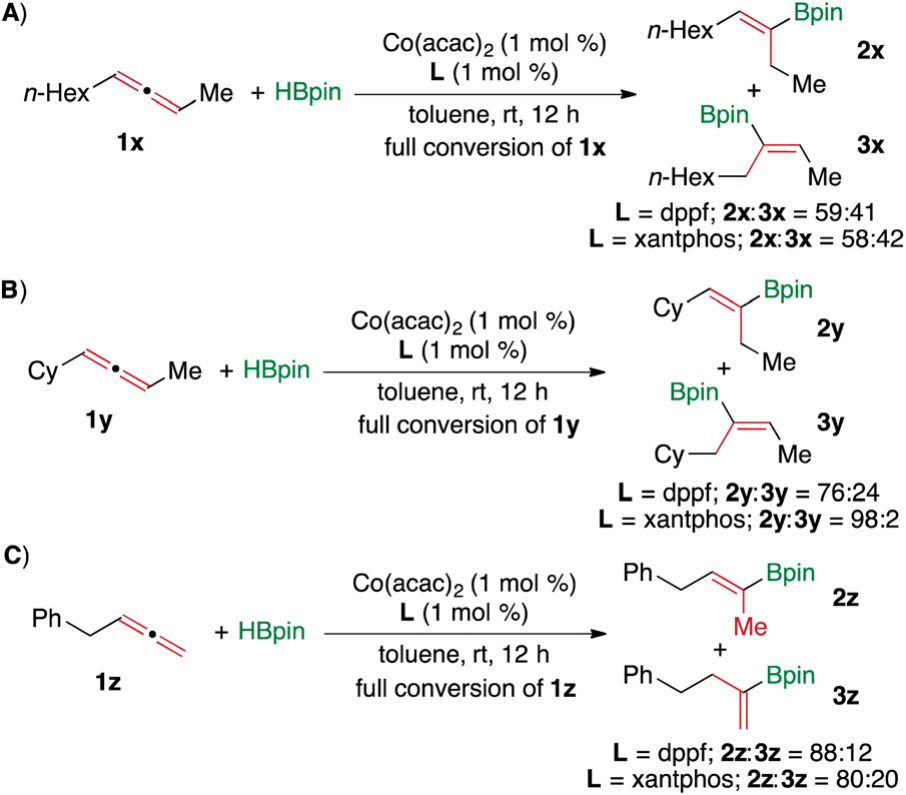
Hydroboration of alkyl,alkyl-1,3-disubstituted allenes and a terminal allene.

According to the proposed mechanism, it is anticipated that this Co-catalyzed regiodivergent hydroboration will not be achieved for alkyl,alkyl-1,3-disubstituted allenes because the two double bonds in these allenes are only different in terms of steric hindrance. Indeed, the hydroboration of *n*-hexyl, methyl-1,3-disubstituted allene **1x** yielded alkenylboronates **2x** and **3x** with similar regioselectivity for both cobalt catalysts (Scheme 6A). Furthermore, increasing the steric difference between the two alkyl substituents allows this hydroboration to proceed selectively on the less hindered double bond of allenes. For example, the hydroboration of cyclohexyl, methyl-1, 3-disubstituted allene **1y** took place in the presence of Co(acac)_2_/xantphos to afford alkenylboronate **2y** with 98% regioselectivity (Scheme 6B). In addition, we also tested a terminal allene for this Co-catalyzed hydroboration. Similarly, the hydroboration of terminal allene **1z** also occurred on the sterically more accessible bond, yielding alkenylboronate **2z** as the major product for both cobalt catalysts (Scheme 6C).

## Conclusions

In summary, we have developed an effective protocol for the synthesis of (*Z*)-alkenylboronates through a ligand-controlled Co-catalyzed stereoselective and regioselective hydroboration of internal allenes. The cobalt catalysts are generated *in situ* from bench stable Co(acac)_2_ and dppf or xantphos ligands and are activated by the reaction with HBpin. A variety of internal aryl,alkyl-substituted allenes underwent this hydroboration with HBpin added to the alkyl-substituted or aryl-substituted carbon–carbon double bond in the presence of Co(acac)_2_/dppf and Co(acac)_2_/xantphos, respectively. Preliminary mechanistic studies suggest that this Co-catalyzed allene hydroboration proceeds through a cobalt-boryl intermediate and that the regioselectivity of this allene hydroboration is controlled by the synergy between the substrates and cobalt catalysts. This Co-catalyzed allene hydroboration provides a useful approach to access synthetically versatile trisubstituted alkenylboronates with commercially available cobalt catalysts.

## Experimental details

### General procedures for this cobalt-catalyzed stereoselective regiodivergent hydroboration of internal allenes

In an Ar-filled glovebox, Co(acac)_2_ (12.0 μmol), dppf or xantphos (12 μmol), allene (0.400 mmol), HBpin (0.440 mmol), and toluene (1 mL) were added into a 4 mL screw-capped vial containing a magnetic stirring bar. The vial was sealed with a cap containing a PTFE septum and removed from the dry box. The reaction mixture was stirred at room temperature for 12 h, and the crude product was purified by column chromatography on silica gel with a mixture of hexane and ethyl acetate as the eluent. The conditions for flash chromatography and data for the characterization of the products are listed in the ESI.†

## Conflicts of interest

There are no conflicts to declare.

## Supplementary Material

SC-011-C9SC06136C-s001
