# Supplementary material for: Ligand-controlled cobalt-catalyzed regiodivergent hydroboration of aryl,alkyl-disubstituted internal allenes
Source: Chem Sci. 2020 Feb 5;11(10):2783–9. doi: 10.1039/c9sc06136c (PMC8157611; doi:10.1039/c9sc06136c)
Supplement: SC-011-C9SC06136C-s001 [file SC-011-C9SC06136C-s001.pdf]

Electronic Supplementary Material (ESI) for Chemical Science.
This journal is © The Royal Society of Chemistry 2020



# Ligand-controlled cobalt-catalyzed regiodivergent hydroboration of aryl,alkyl-disubstituted internal allenes

Caizhi Wu, and Shaozhong Ge*

Department of Chemistry, National University of Singapore, 3 Science Drive 3, 117543, Singapore

Email: chmgsh@nus.edu.sg

## Table of Content

**I. General Remarks …………………………………………………………… S2**

**II. General Procedure for the Co-catalyzed Hydroboration of Internal Allene with $Co(acac)_2$/dppf ……………………………………………………… S2**

**III. General Procedure for the Co-catalyzed Hydroboration of Internal Allene with $Co(acac)_2$/xantphos ……………………………………………………… S9**

**IV. Procedures for Gram-Scale Reaction ……………………………………… S14**

**V. Procedures of the Transformations of (*Z*)-Alkenylboronate 2k …………... S15**

**VI. The Procedure for the Co-catalyzed Hydrosilylation of 1,3-Disubstituted Allene with dppf as Ligand ………………………………………………... S16**

**VII. General Procedure for the Co-catalyzed Hydrosilylation of 1,3-Disubstituted Allene with Xantphos as Ligand ……………………………………….. S16**

**VIII. Procedures of Deuterium-labeling experiments ………………………… S17**

**IX. General procedure of the Preparation of Allene Substrates……………… S20**

**X. References ..……………………………………………………..……….. S25**

**XI. Copies of $^1$H NMR and $^{13}C\{^1H\}$ NMR Spectra ……………..………..……….. S26**

## I. General Remarks

All the manipulations were performed in an argon-filled glovebox, unless mentioned otherwise. THF was purified by passing the degassed solvents ($N_2$) through a column of activated alumina (solvent purification system purchased from Innovative Technologies, Newburyport, MA). The following chemicals were purchased and used as received: $Co(acac)_2$ (>99.0%, Sigma-Aldrich), pinacolborane (HBpin) (98.0%, Oakwoood), and phosphine ligand (Sigma-Aldrich). All other reagents and solvents were purchased from commercial sources and used without purification.

$^1H$ and $^{13}C\{^1H\}$ spectra were recorded using Bruker 400 MHz, or 500 MHz NMR spectrometers. $^1H$ NMR and $^{13}C\{^1H\}$ NMR spectra were referenced to resonances of the residual signals of the deuterated solvents. Multiplicities are recorded as: s = singlet, d = doublet, t = triplet, dd = doublet of doublets, dt = doublet of triplets, and m = multiplet. GC-MS analysis was performed on Shimadzu GC-2010 gas chromatograph coupled to a Shimadzu QP2010 mass selective detector. HR-MS analyses were performed using Thermo Scientific Exactive (APCI).

## II. General Procedure for the Co-catalyzed Hydroboration of Internal Allene with Co(acac)₂/dppf

In an Ar-filled glovebox, $Co(acac)_2$ (3.1 mg, 12.0 μmol), dppf (6.7 mg,12.0 μmol), allene (0.400 mmol), HBpin (56.3 mg, 0.440 mmol), and toluene (1 mL) were added to a 4-mL screw-capped vial containing a magnetic stirring bar. The vial was sealed with a cap containing a PTFE septum and removed from the dry box. The reaction mixture was stirred at room temperature for 12 h, and the crude product was purified by column chromatography silica gel with a mixture of hexane and ethyl acetate as eluent. The conditions for flash chromatography on silica and data for characterization of the (*Z*)-alkenylboronate products are listed below.

### (*Z*)-4,4,5,5-Tetramethyl-2-(1-phenylbut-1-en-2-yl)-1,3,2-dioxaborolane (2a)

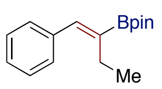


The title compound was isolated (82.8 mg, 81%) as colourless oil after chromatography (80:1 hexane/EtOAc). $^1H$ NMR (500 MHz, $C_6D_6$) δ 7.69 (s, 1H), 7.32 (d, *J* = 8.1 Hz, 2H), 7.11 (t, *J* = 6.9 Hz, 2H), 7.01 (t, *J* = 6.7 Hz, 1H), 2.64 (q, *J* = 7.5 Hz, 2H), 1.26 (t, *J* = 7.5 Hz, 3H), 1.10 (s, 12H). $^{13}C\{^1H\}$ NMR (126 MHz, $C_6D_6$) δ 142.3, 138.2, 129.1, 128.3, 127.2, 83.1, 24.7, 22.9, 14.8. The boron-bound carbon was not detected due to quadrupolar relaxation. IR: $\nu_{max}$(nujol) 3057, 3024, 2997, 1618, 1492. HR-MS (ESI) *m/z*: calcd for $C_{16}H_{23}BNaO_2$ ($[M+Na]^+$): 281.1686; Found: 281.1698. The seterochemistry of the double bond was confirmed by 1D selective NOE analysis.

### (*Z*)-4,4,5,5-Tetramethyl-2-(1-phenylhex-1-en-2-yl)-1,3,2-dioxaborolane (2b)

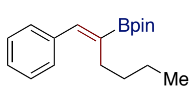


The title compound was isolated (80.1 mg, 70%) as colourless oil after chromatography (80:1 hexane/EtOAc). After purification, **2b**:**3b** = 100:8.5. $^1H$ NMR (500 MHz, $C_6D_6$) δ 7.76 (s, 1H), 7.36 (d, *J* = 8.2 Hz, 2H), 7.12 (t, *J* = 7.7 Hz, 2H), 7.01 (t, *J* = 7.4 Hz, 1H), 2.70–2.66 (m, 2H), 1.77–1.67 (m, 3H), 1.39–1.35 (m, 2H), 1.11 (s, 12H), 0.88 (t, *J* = 7.4 Hz, 3H). $^{13}C\{^1H\}$

NMR (126 MHz, $C_6D_6$) δ 143.7, 139.3, 130.0, 129.2, 128.0, 84.0, 33.5, 30.4, 25.6, 24.0, 14.9. The boron-bound carbon was not detected due to quadrupolar relaxation. IR: $\nu_{max}$(nujol) 3056, 3025, 2989, 1615, 1491, 1476. HR-MS (ESI) *m/z*: calcd for $C_{18}H_{27}BNaO_2$ ($[M+Na]^+$): 309.1996; Found: 309.2004.

**(*Z*)-4,4,5,5-Tetramethyl-2-(1-phenyloct-1-en-2-yl)-1,3,2-dioxaborolane (2c)**

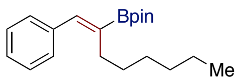


The title compound was isolated (85.5mg, 68%) as colourless oil after chromatography (80:1 hexane/EtOAc). $^1$H NMR (500 MHz, $C_6D_6$) δ 7.78 (s, 1H), 7.37 (d, *J* = 7.2 Hz, 2H), 7.12 (d, *J* = 7.8 Hz, 2H), 7.01 (t, *J* = 7.4 Hz, 1H), 2.70 (t, *J* = 7.9 Hz, 2H), 1.79–1.71 (m, 2H), 1.40–1.34 (m, 2H), 1.28–1.21 (m, 4H), 1.12 (s, 12H), 0.86–0.82 (m, 3H). $^{13}C\{^1H\}$ NMR (126 MHz, $C_6D_6$) δ 143.6, 139.3, 130.0, 129.2, 128.0, 84.0, 32.8, 31.3, 30.6, 30.6, 25.6, 23.7, 15.0. The boron-bound carbon was not detected due to quadrupolar relaxation. IR: $\nu_{max}$(nujol) 3055, 3025, 2989, 1614, 1488. HR-MS (ESI) m/z: calcd for $C_{20}H_{31}BNaO_2$ ($[M+Na]^+$): 337.2313; Found: 337.2322.

**(*Z*)-2-(4-Cyclopentyl-1-phenylbut-1-en-2-yl)-4,4,5,5-tetramethyl-1,3,2-dioxaborolane (2d)**

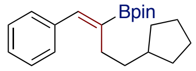


The title compound was isolated (96.5 mg, 74%) as colourless oil after chromatography (80:1 hexane/EtOAc). After purification, **2d**:**3d** = 100:6. $^1$H NMR (500 MHz, $C_6D_6$) δ 7.77 (s, 1H), 7.40 (d, *J* = 7.4 Hz, 2H), 7.17 (d, *J* = 7.6 Hz, 2H), 7.05 (t, *J* = 7.4 Hz, 1H), 2.73 (ddd, *J* = 8.7, 6.5, 1.2 Hz, 2H), 1.83–1.72 (m, 5H), 1.61–1.54 (m, 2H), 1.50–1.43 (m, 2H), 1.22–1.16 (m, 2H), 1.15 (s, 12H). $^{13}C\{^1H\}$ NMR (126 MHz, $C_6D_6$) δ 143.5, 139.3, 130.0, 129.2, 128.0, 84.0, 41.6, 37.6, 33.7, 29.9, 26.2, 25.6. The boron-bound carbon was not detected due to quadrupolar relaxation. HR-MS (APCI) *m/z*: calcd for $C_{21}H_{31}BNaO_2$ ($[M+Na]^+$): 349.2313; Found: 349.2315.

**(*Z*)-4,4,5,5-Tetramethyl-2-(1-(o-tolyl)but-1-en-2-yl)-1,3,2-dioxaborolane (2g)**

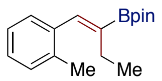


The title compound was isolated (88.2mg 81%) as colourless oil after chromatography (80:1 hexane/EtOAc). $^1$H NMR (400 MHz, $CDCl_3$) δ 7.23 (s, 1H), 7.17–7.14 (m, 3H), 7.09–7.05 (m, 1H), 2.26 (s, 3H), 2.20 (q, *J* = 7.8 Hz, 2H), 1.33 (s, 12H), 1.01 (t, *J* = 7.5 Hz, 3H). $^{13}C\{^1H\}$ NMR (101 MHz, $CDCl_3$) δ 141.1, 137.5, 136.1, 129.7, 128.7, 127.0, 125.2, 83.3, 24.8, 22.8, 20.0, 14.8. The boron-bound carbon was not detected due to quadrupolar relaxation. IR: $\nu_{max}$(nujol) 2989, 1618, 1484. HR-MS (APCI) *m/z*: calcd for $C_{17}H_{25}BNaO_2$ ($[M+Na]^+$): 295.1843; Found: 295.1848.

**(*Z*)-4,4,5,5-Tetramethyl-2-(1-(m-tolyl)but-1-en-2-yl)-1,3,2-dioxaborolane (2h)**

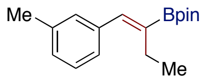


The title compound was isolated (82.7 mg, 76%) as colourless oil after chromatography (80:1 hexane/EtOAc). $^1$H NMR (500 MHz, $C_6D_6$) δ 7.72 (s, 1H), 7.26–7.20 (m, 1H), 7.15–7.10 (m, 1H), 7.08 (t, *J* = 7.6 Hz, 1H), 6.89–6.86 (m, 1H), 2.69 (qd, *J* = 7.5, 1.1 Hz, 2H), 2.08 (d, *J* = 0.8 Hz, 3H), 1.29 (t, *J* = 7.5 Hz, 3H), 1.12 (s, 12H). $^{13}C\{^1H\}$ NMR (126 MHz, $C_6D_6$) δ 143.5, 139.2, 138.5, 130.9, 129.1, 127.0, 84.0, 25.6, 23.9, 22.1, 15.7. The boron-bound

carbon was not detected due to quadrupolar relaxation. IR: ν$_{max}$(nujol) 2992, 1617, 1481. GC-MS (EI): calculated for $C_{17}H_{25}BO_2$: 272.19; found: 272.20.

**(*Z*)-4,4,5,5-Tetramethyl-2-(1-(p-tolyl)but-1-en-2-yl)-1,3,2-dioxaborolane (2i)**

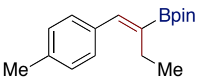


The title compound was isolated (83.8 mg, 77%) as colourless oil after chromatography (80:1 hexane/EtOAc). $^1$H NMR (500 MHz, $C_6D_6$) δ 7.69 (s, 1H), 7.27 (d, *J* = 7.9 Hz, 2H), 6.94 (d, *J* = 7.8 Hz, 2H), 2.68 (q, *J* = 7.4 Hz, 2H), 2.07 (s, 3H), 1.29 (t, *J* = 7.4 Hz, 3H), 1.11 (s, 12H). $^{13}$C{$^1$H} NMR (126 MHz, $C_6D_6$) δ 143.3, 137.6, 136.3, 130.1, 129.9, 84.0, 25.6, 23.9, 21.8, 15.7. The boron-bound carbon was not detected due to quadrupolar relaxation. IR: ν$_{max}$(nujol) 3086, 3064, 3027, 2997, 1633, 1603, 1491. HR-MS (ESI) m/z: calcd for $C_{17}H_{25}BNaO_2$ ([M+Na]$^+$): 295.1843; Found: 295.1841.

**(*Z*)-2-(1-(4-(tert-Butyl)phenyl)but-1-en-2-yl)-4,4,5,5-tetramethyl-1,3,2-dioxaborolane (2j)**

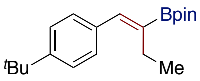


The title compound was isolated (98.1 mg, 78%) as colourless oil after chromatography (80:1 hexane/EtOAc). $^1$H NMR (500 MHz, $C_6D_6$) δ 7.78 (s, 1H), 7.37 (d, *J* = 8.1 Hz, 2H), 7.20 (d, *J* = 8.2 Hz, 2H), 2.74 (q, *J* = 7.5 Hz, 2H), 1.34 (t, *J* = 7.5 Hz, 3H), 1.18 (s, 9H), 1.11 (s, 12H). $^{13}$C{$^1$H} NMR (126 MHz, $C_6D_6$) δ 150.8, 143.2, 136.4, 130.0, 126.2, 84.0, 35.2, 32.0, 25.6, 23.9, 15.8. The boron-bound carbon was not detected due to quadrupolar relaxation. IR: ν$_{max}$(nujol) 3086, 3050, 3024, 2997, 1669, 1618, 1513, 1484. GC-MS (EI): calculated for $C_{20}H_{31}BO_2$: 314.24; found: 314.30.

**(*Z*)-2-(1-(4-Methoxyphenyl)but-1-en-2-yl)-4,4,5,5-tetramethyl-1,3,2-dioxaborolane (2k)**

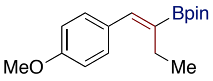


The title compound was isolated (89.9 mg, 78%) as colourless oil after chromatography (60:1 hexane/EtOAc). $^1$H NMR (500 MHz, $C_6D_6$) δ 7.70 (s, 1H), 7.32–7.29 (m, 2H), 6.71–6.68 (m, 2H), 3.26 (s, 3H), 2.72 (q, *J* = 7.5 Hz, 2H), 1.32 (t, *J* = 7.5 Hz, 3H), 1.13 (s, 12H). $^{13}$C{$^1$H} NMR (126 MHz, $C_6D_6$) δ 160.1, 143.0, 131.7, 131.5, 114.8, 83.9, 55.4, 25.6, 23.8, 15.6. The boron-bound carbon was not detected due to quadrupolar relaxation. IR: ν$_{max}$(nujol) 3063, 2997, 1610, 1573, 1514, 1481. HR-MS (APCI) *m/z*: calcd for $C_{17}H_{25}BO3$([M+Na]$^+$): 311.1792; Found: 311.1789.

**(*Z*)-4,4,5,5-Tetramethyl-2-(1-(4-(trifluoromethyl)phenyl)but-1-en-2-yl)-1,3,2-dioxaborolane (2l)**

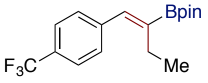


The title compound was isolated (102 mg, 78%) as colourless oil after chromatography (80:1 hexane/EtOAc). $^1$H NMR (400 MHz, $C_6D_6$) δ 7.50 (s, 1H), 7.25 (d, *J* = 8.1 Hz, 2H), 7.07 (d, *J* = 7.4 Hz, 2H), 2.48 (q, *J* = 7.2 Hz, 2H), 1.19 (t, *J* = 7.5 Hz, 3H), 1.11 (s, 12H). $^{13}$C{$^1$H} NMR (101 MHz, $C_6D_6$) δ 141.4, 140.3, 130.0, 128.7(q, *J* = 32.4 Hz), 125.0(q, *J* = 3.8 Hz), 124.6(q, *J* = 272.8 Hz), 83.2, 24.5, 22.7, 14.4. The boron-bound carbon was not detected due to quadrupolar relaxation. IR: ν$_{max}$(nujol) 2989, 1618, 1478. GC-MS (EI): calculated for $C_{22}H_{34}B_2O_4$: 384.26; Found: 326.15.

**(*Z*)-2-(1-(4-Fluorophenyl)but-1-en-2-yl)-4,4,5,5-tetramethyl-1,3,2-dioxaborolane (2m)**

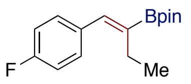


The title compound was isolated (86.2 mg, 78%) as colourless oil after chromatography (80:1 hexane/EtOAc). $^1$H NMR (500 MHz, $C_6D_6$) δ 7.55 (s, 1H), 7.11–7.04 (m, 2H), 6.77–6.68 (m, 2H), 2.55 (qd, *J* = 7.5, 1.2 Hz, 2H), 1.22 (t, *J* = 7.5 Hz, 3H), 1.11 (s, 12H). $^{13}$C{$^1$H} NMR (126 MHz, $C_6D_6$) δ 163.0(d, *J* = 246.9 Hz), 141.9, 135.1(d, *J* = 3.4 Hz), 131.7(d, *J* = 8.0 Hz), 116.0(d, *J* = 21.6 Hz), 84.1, 25.6, 23.7, 15.5. The boron-bound carbon was not detected due to quadrupolar relaxation. IR: $\nu_{max}$(nujol) 2992, 1621, 1617, 1486. GC-MS (EI): calculated for $C_{16}H_{22}BFO_2$: 276.17; Found: 276.15.

**(*Z*)-2-(1-(4-Chlorophenyl)but-1-en-2-yl)-4,4,5,5-tetramethyl-1,3,2-dioxaborolane (2n)**

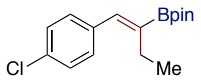


The title compound was isolated (96.0 mg, 82% as colourless oil after chromatography (80:1 hexane/EtOAc). $^1$H NMR (500 MHz, $C_6D_6$) δ 7.50 (s, 1H), 7.04–6.99 (m, 4H), 2.52 (q, *J* = 7.4 Hz, 2H), 1.20 (t, *J* = 7.5 Hz, 3H), 1.10 (s, 12H). $^{13}$C{$^1$H} NMR (126 MHz, $C_6D_6$) δ 141.7, 137.4, 133.9, 131.2, 129.4, 84.1, 25.6, 23.7, 15.5. The boron-bound carbon was not detected due to quadrupolar relaxation. IR: $\nu_{max}$(nujol) 3078, 2987, 1615, 1491. GC-MS (EI): calculated for $C_{16}H_{22}BClO_2$: 292.14; Found: 292.15.

**(*Z*)-2-(1-(4-Bromophenyl)but-1-en-2-yl)-4,4,5,5-tetramethyl-1,3,2-dioxaborolane (2o)**

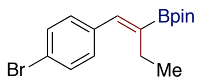


The title compound was isolated (105 mg, 78%) as colourless oil after chromatography (80:1 hexane/EtOAc). $^1$H NMR (500 MHz, $C_6D_6$) δ 7.47 (s, 1H), 7.19–7.16 (m, 2H), 6.94–6.90 (m, 2H), 2.51 (q, *J* = 7.4 Hz, 2H), 1.19 (t, *J* = 7.5 Hz, 3H), 1.10 (s, 12H). $^{13}$C{$^1$H} NMR (126 MHz, $C_6D_6$) δ 141.7, 137.8, 132.3, 131.5, 122.1, 84.1, 25.6, 23.7, 15.5. The boron-bound carbon was not detected due to quadrupolar relaxation. IR: $\nu_{max}$(nujol) 3083, 2989, 1615, 1484. GC-MS (EI): calculated for $C_{16}H_{22}BBrO_2$: 336.09; found: 336.10.

**(*Z*)-2-(1-(4-Iodophenyl)but-1-en-2-yl)-4,4,5,5-tetramethyl-1,3,2-dioxaborolane (2p)**

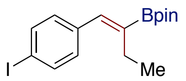


The title compound was isolated (115 mg, 75%) as colourless oil after chromatography (80:1 hexane/EtOAc). $^1$H NMR (500 MHz, $C_6D_6$) δ 7.46 (s, 1H), 7.39–7.34 (m, 2H), 6.81–6.77 (m, 2H), 2.51 (q, *J* = 7.5 Hz, 2H), 1.19 (t, *J* = 7.5 Hz, 3H), 1.10 (s, 12H). $^{13}$C{$^1$H} NMR (126 MHz, $C_6D_6$) δ 141.9, 138.3 (two peaks overlap), 131.7, 93.7, 84.1, 25.6, 23.7, 15.5. The boron-bound carbon was not detected due to quadrupolar relaxation. IR: $\nu_{max}$(nujol) 3071, 2992, 1621, 1486. HR-MS (APCI) *m/z*: calcd for $C_{16}H_{22}BINaO_2$ ([M+Na]$^+$): 407.0653; Found: 407.0550.

**(*Z*)-Trimethyl(4-(2-(4,4,5,5-tetramethyl-1,3,2-dioxaborolan-2-yl)but-1-en-1-yl)phenyl)silane (2q)**

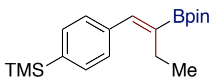

The title compound was isolated (111 mg, 84%) as colourless oil after chromatography (80:1 hexane/EtOAc). $^{1}H$ NMR (500 MHz, $C_6D_6$) δ 7.77 (s, 1H), 7.39 (s, 4H), 2.71 (q, *J* = 7.5 Hz, 2H), 1.32 (t, *J* = 7.5 Hz, 3H), 1.12 (s, 12H), 0.18 (s, 10H). $^{13}C\{^{1}H\}$ NMR (126 MHz, $C_6D_6$) δ 143.3, 139.9, 139.6, 134.4, 129.4, 84.1, 25.6, 24.0, 15.8, -0.4. The boron-bound carbon was not detected due to quadrupolar relaxation. IR: $\nu_{max}$(nujol) 3060, 2998, 1618, 1596, 1483. HR-MS (ACPI) m/z: calcd for $C_{19}H_{31}BO_2Si$ $[M]^+$: 330.2185; Found: 330.2182.

**(*Z*)-4-(2-(4,4,5,5-Tetramethyl-1,3,2-dioxaborolan-2-yl)but-1-en-1-yl)benzonitrile (2r)**

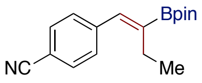

The title compound was isolated (89.4 mg, 79%) as colourless oil after chromatography (15:1 hexane/EtOAc). $^{1}H$ NMR (500 MHz, $C_6D_6$) δ 7.35 (s, 1H), 6.94 (d, *J* = 8.3 Hz, 2H), 6.86 (d, *J* = 8.5 Hz, 2H), 2.39 (q, *J* = 7.5 Hz, 2H), 1.13 (t, *J* = 7.5 Hz, 3H), 1.10 (s, 12H). $^{13}C\{^{1}H\}$ NMR (126 MHz, $C_6D_6$) δ 142.8, 140.9, 132.6, 130.0, 119.5, 111.9, 84.4, 25.5, 23.8, 15.3. The boron-bound carbon was not detected due to quadrupolar relaxation. IR: $\nu_{max}$(nujol) 3004, 2229, 1602, 1499, 1484. HR-MS (ESI) m/z: calcd for $C_{17}H_{22}BNNaO_2$ ($[M+Na]^+$): 306.1639; Found: 306.1650.

**(*Z*)-4,4,5,5-Tetramethyl-2-(4-(2-(4,4,5,5-tetramethyl-1,3,2-dioxaborolan-2-yl)but-1-en-1-yl)phenyl)-1,3,2-dioxaborolane (2s)**

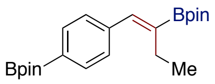

The title compound was isolated (131 mg, 85%) as colourless oil after chromatography (10:1 hexane/EtOAc). $^{1}H$ NMR (500 MHz, $C_6D_6$) δ 8.09 (d, *J* = 8.0 Hz, 2H), 7.64 (s, 1H), 7.35 (d, *J* = 8.1 Hz, 2H), 2.60 (q, *J* = 7.4 Hz, 2H), 1.20 (t, *J* = 7.5 Hz, 3H), 1.11 (s, 12H), 1.10 (s, 12H). $^{13}C\{^{1}H\}$ NMR (126 MHz, $C_6D_6$) δ 142.3, 141.1, 135.2, 128.4, 83.4, 83.1, 24.7 (two peaks overlap), 22.9, 14.6. The boron-bound carbon was not detected due to quadrupolar relaxation. IR: $\nu_{max}$(nujol) 2982, 1610, 1468. GC-MS (EI): calculated for $C_{22}H_{34}B_2O_4$: 384.26; Found: 384.25.

**(Z)-4,4,5,5-Tetramethyl-2-(1-(4-(methylthio)phenyl)but-1-en-2-yl)-1,3,2-dioxaborolane (2t)**

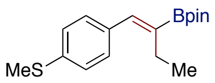

The title compound was isolated (66.9 mg, 55%) as colourless oil after chromatography (60:1 hexane/EtOAc). $^{1}H$ NMR (500 MHz, $C_6D_6$) δ 7.32–7.29 (m, 2H), 7.13–7.09 (m, 2H), 2.76 (q, *J* = 7.8, 7.4 Hz, 2H), 2.06 (s, 3H), 1.39 (t, *J* = 7.5 Hz, 3H), 1.23 (s, 12H). $^{13}C\{^{1}H\}$ NMR (126 MHz, $C_6D_6$) δ 142.7, 139.0, 135.7, 130.5, 127.1, 84.1, 25.6, 23.9, 15.8, 15.6. The boron-bound carbon was not detected due to quadrupolar relaxation. HR-MS (APCI) *m/z*: calcd for $C_{17}H_{26}BO_2S$ ($[M+H]^+$): 305.1744; Found: 305.1739.

**Methyl-(Z)-4-(2-(4,4,5,5-tetramethyl-1,3,2-dioxaborolan-2-yl)but-1-en-1-yl)benzoate (2u)**

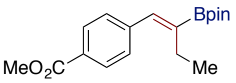


The title compound was isolated (108 mg, 82%) as colourless oil after chromatography (15:1 hexane/EtOAc). $^1$H NMR (500 MHz, $C_6D_6$) δ 8.04–8.01 (m, 2H), 7.54 (d, $J$ = 3.8 Hz, 1H), 7.21 (d, $J$ = 8.2 Hz, 2H), 3.49 (s, 3H), 2.56–2.48 (m, 2H), 1.18 (td, $J$ = 7.5, 2.2 Hz, 3H), 1.10 (s, 12H). $^{13}$C{$^1$H} NMR (126 MHz, $C_6D_6$) δ 166.2, 142.5, 141.0, 129.7, 129.1, 128.9, 83.3, 51.3, 24.6, 23.0, 14.6. The boron-bound carbon was not detected due to quadrupolar relaxation. IR: $\nu_{max}$(nujol) 3081, 2997, 1728, 1610, 1564, 1503, 1485. HR-MS (APCI) *m/z*: calcd for $C_{18}H_{25}BNaO_4$ ([M+Na]$^+$): 339.1741; Found: 339.1743.

**(*Z*)-4,4,5,5-tetramethyl-2-(1-(thiophen-3-yl)but-1-en-2-yl)-1,3,2-dioxaborolane (2v)**

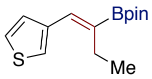


The title compound was isolated (77.1 mg, 73%) as colourless oil after chromatography (80:1 hexane/EtOAc). $^1$H NMR (500 MHz, $C_6D_6$) δ 7.60 (s, 1H), 7.05–7.00 (m, 2H), 6.82–6.78 (m, 1H), 2.65 (q, $J$ = 7.5 Hz, 2H), 1.24 (d, $J$ = 7.5 Hz, 3H), 1.10 (s, 12H). $^{13}$C NMR (126 MHz, $C_6D_6$) δ 140.3, 137.0, 129.8, 126.0, 125.7, 84.0, 25.6, 24.2, 15.2. The boron-bound carbon was not detected due to quadrupolar relaxation. IR: $\nu_{max}$(nujol) 3103, 2997, 1728, 1610, 1564, 1503, 1485. HR-MS (APCI) m/z: calcd for $C_{14}H_{22}BO_2S$ ([M+H]$^+$): 265.1431; Found: 265.1432.

**(*Z*)-2-(1-(5-(methoxymethyl)furan-3-yl)but-1-en-2-yl)-4,4,5,5-tetramethyl-1,3,2-dioxaborolane (2w)**

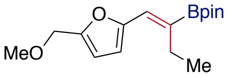


The title compound was isolated (83.0 mg, 71%,) as colourless oil after chromatography (30:1 hexane/EtOAc). $^1$H NMR (500 MHz, $C_6D_6$) δ 7.42 (s, 1H), 6.19 (d, $J$ = 3.3 Hz, 1H), 6.05 (d, $J$ = 3.3 Hz, 1H), 4.10 (s, 2H), 3.06 (s, 3H), 2.87 (q, $J$ = 7.5 Hz, 2H), 1.31 (t, $J$ = 7.5 Hz, 3H), 1.08 (s, 12H). $^{13}$C{$^1$H} NMR (126 MHz, $C_6D_6$) δ 154.8, 153.4, 130.1, 113.4, 111.7, 84.1, 67.2, 58.2, 25.6, 24.6, 15.1. The boron-bound carbon was not detected due to quadrupolar relaxation. IR: $\nu_{max}$(nujol) 2996, 1784, 1684, 1620, 1511, 1481. GC-MS (EI): calculated for $C_{16}H_{25}BO_4$ ([M]$^+$): 292.18; Found: 292.20.

**(*Z*)-2-(dec-3-en-3-yl)-4,4,5,5-tetramethyl-1,3,2-dioxaborolane (2x)**

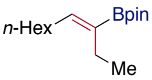


The title compound was isolated (84.1 mg, 79%, mixture of **2x** and **3x**) as colourless oil after chromatography (80:1 hexane/EtOAc).

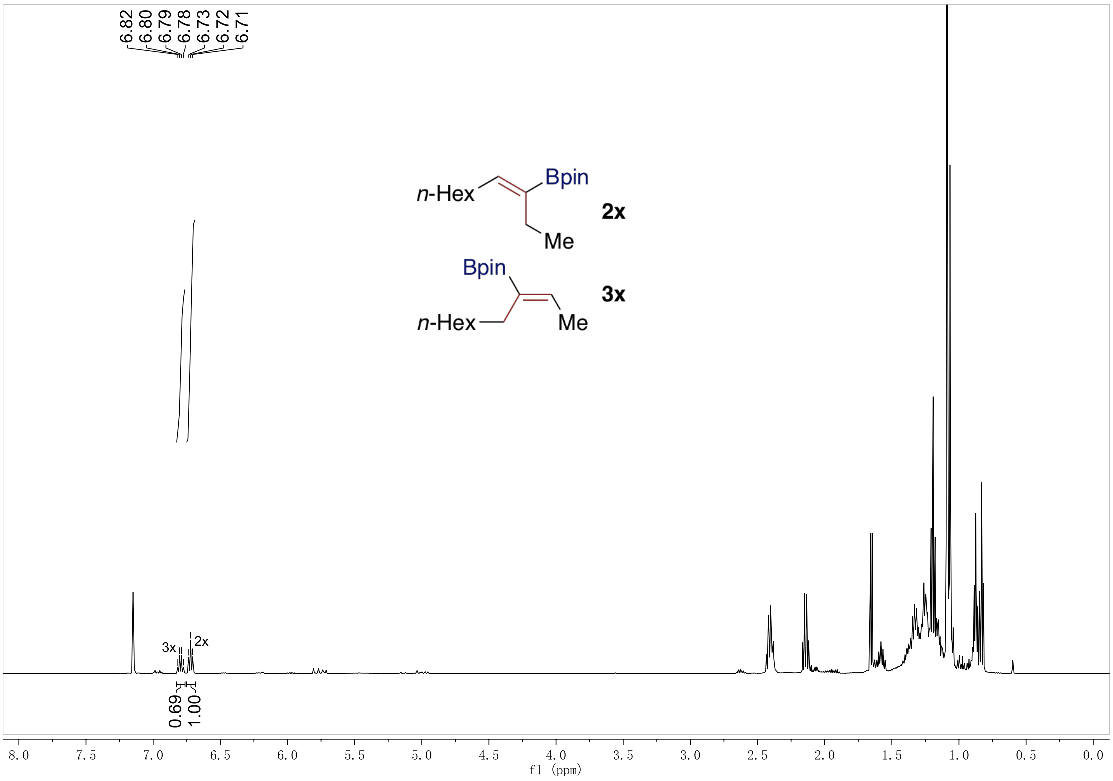


**(*Z*)-2-(1-cyclohexylbut-1-en-2-yl)-4,4,5,5-tetramethyl-1,3,2-dioxaborolane (2y)**

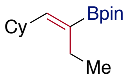


The title compound was isolated (78.2 mg, 74%, mixture of **2y** and **3y**) as colourless oil after chromatography (30:1 hexane/EtOAc).

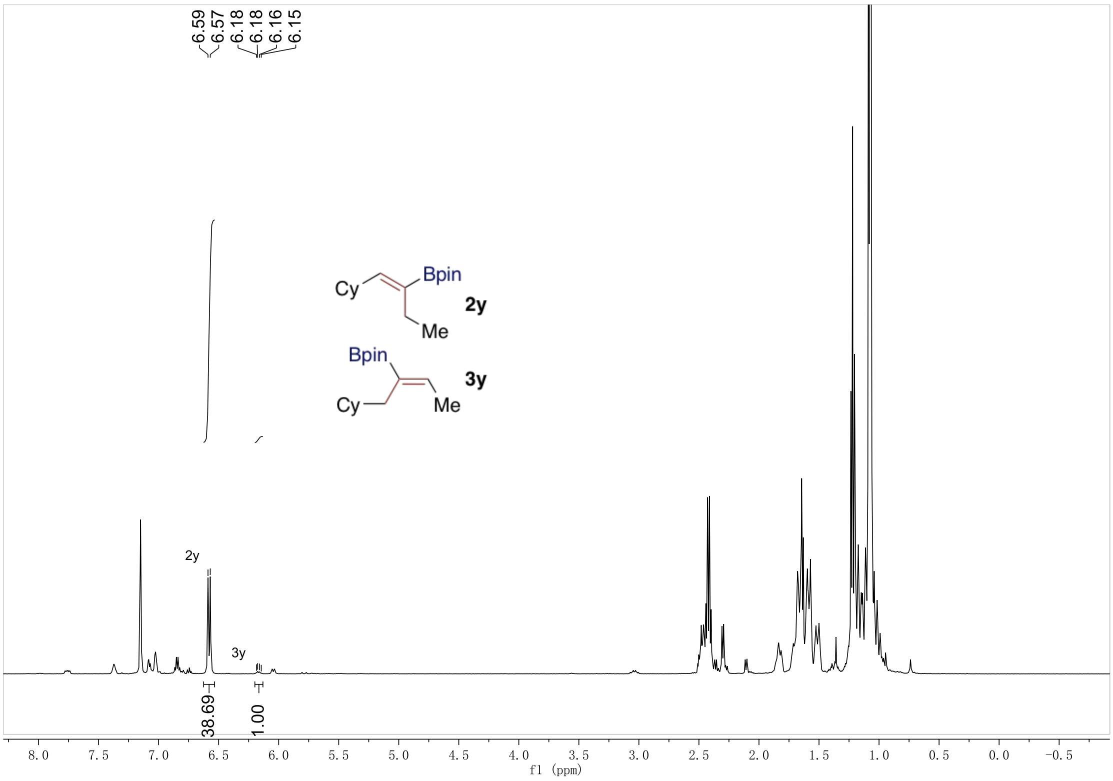

**(*Z*)-4,4,5,5-tetramethyl-2-(4-phenylbut-2-en-2-yl)-1,3,2-dioxaborolane (2z)**

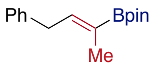


The title compound was isolated (83.0 mg, 71%,) as colourless oil after chromatography (30:1 hexane/EtOAc). $^1$H NMR (500 MHz, $C_6D_6$) δ 7.27 (s, 1H), 7.22–7.18 (m, 3H), 7.16–7.14 (m, 1H), 7.04–7.01 (m, 1H), 3.49 (d, *J* = 7.1 Hz, 2H), 2.09 (s, 3H), 1.18 (s, 12H). $^{13}$C NMR (126 MHz, $C_6D_6$) δ 145.8, 141.4, 129.5, 129.4, 126.7, 83.8, 36.00, 25.6, 14.9. GC-MS (EI): calculated for $C_{16}H_{23}BO_2$ ([M]$^+$): 258.18; Found: 258.20.

## III. General Procedure for the Co-catalyzed Hydroboration of Internal Allene with Co(acac)$_2$/xantphos

In an Argon-filled glovebox, Co(acac)$_2$ (3.1 mg, 12.0 μmol), xantphos (6.9 mg,12.0 μmol), allene (0.400 mmol), HBpin (56.3 mg, 0.44 mmol), and toluene (1 mL) were added to a 4-mL screw-capped vial containing a magnetic stirring bar. The vial was sealed with a cap containing a PTFE septum and removed from the dry box. The reaction mixture was stirred at room temperature for 12 h, and the crude product was purified by column chromatography on silica gel with a mixture of hexane and ethyl acetate as eluent. The conditions for flash chromatography and data for characterization of the (*Z*)-alkenylboronate products are listed below.

**(*Z*)-4,4,5,5-Tetramethyl-2-(1-phenylbut-2-en-2-yl)-1,3,2-dioxaborolane (3a)**

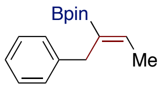


The title compound was isolated (82.6 mg, 80%,) as colourless oil after chromatography (80:1 hexane/EtOAc). $^1$H NMR (500 MHz, $C_6D_6$) δ 7.43 (d, *J* = 7.5 Hz, 2H), 7.29 (t, *J* = 7.6 Hz, 3H), 7.16 (dd, *J* = 10.5, 4.2 Hz, 1H), 6.96 (q, *J* = 6.1 Hz, 1H), 3.77 (s, 2H), 1.72 (d, *J* = 6.8 Hz, 3H), 1.13 (s, 12H). $^{13}$C{$^1$H} NMR (126 MHz, $C_6D_6$) δ 142.1, 141.6, 128.7, 128.2, 125.5, 82.9, 34.0, 24.6, 14.5. The boron-bound carbon was not detected due to quadrupolar relaxation. IR: $\nu_{max}$(nujol) 3088, 3063, 3028, 2989, 1633, 1604, 1493. HR-MS (ESI) *m/z*: calcd for $C_{16}H_{23}BNaO_2$ ([M+Na]$^+$): 281.1686; Found: 281.1687. The seterochemistry of the double bond was confirmed by 1D selective NOE analysis.

**(*Z*)-4,4,5,5-Tetramethyl-2-(1-phenylhex-2-en-2-yl)-1,3,2-dioxaborolane (3b)**

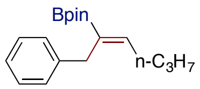


The title compound was isolated (92.7 mg, 81%) as colourless oil after chromatography (80:1 hexane/EtOAc). $^1$H NMR (500 MHz, $C_6D_6$) δ 7.36 (d, *J* = 7.1 Hz, 2H), 7.20 (t, *J* = 7.6 Hz, 2H), 7.06 (t, *J* = 7.4 Hz, 1H), 6.81 (t, *J* = 7.1 Hz, 1H), 3.70 (s, 2H), 2.11 (q, *J* = 7.3 Hz, 2H), 1.29 (h, *J* = 7.4 Hz, 2H), 1.02 (s, 12H), 0.77 (t, *J* = 7.4 Hz, 3H). $^{13}$C{$^1$H} NMR (126 MHz, $C_6D_6$) δ 148.3, 143.1, 129.7, 129.1, 126.4, 83.9, 35.4, 31.9, 25.5, 23.3, 14.8. The boron-bound carbon was not detected due to quadrupolar relaxation. IR: $\nu_{max}$(nujol) 3061, 3025, 2989, 1630, 1604, 1486. HR-MS (ESI) *m/z*: calcd for $C_{18}H_{27}BNaO_2$ ([M+Na]$^+$): 309.1996; Found: 309.2009.

**(*Z*)-4,4,5,5-Tetramethyl-2-(1-phenyloct-2-en-2-yl)-1,3,2-dioxaborolane (3c)**

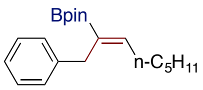


The title compound was isolated (95.5 mg, 76%) as colourless oil after chromatography (80:1 hexane/EtOAc). $^1$H NMR (500 MHz,

$C_6D_6$) δ 7.36 (d, *J* = 7.2 Hz, 2H), 7.19 (t, *J* = 7.7 Hz, 2H), 7.06 (t, *J* = 7.4 Hz, 1H), 6.82 (t, *J* = 7.1 Hz, 1H), 3.70 (s, 2H), 2.15 (q, *J* = 7.3 Hz, 2H), 1.32–1.22 (m, 3H), 1.16–1.13 (m, 3H), 1.02 (s, 12H), 0.80–0.74 (m, 3H). $^{13}C\{^1H\}$ NMR (126 MHz, $C_6D_6$) δ 148.6, 143.2, 129.7, 129.1, 126.4, 83.9, 35.4, 32.6, 29.9, 29.8, 25.5, 23.6, 14.8. The boron-bound carbon was not detected due to quadrupolar relaxation. IR: $\nu_{max}$(nujol) 3025, 2996, 1614, 1511, 1481. HR-MS (ESI) m/z: calcd for $C_{20}H_{31}BNaO_2$ ([M+Na]$^+$): 337.2313; Found: 337.2322.

**(*Z*)-2-(4-Cyclopentyl-1-phenylbut-2-en-2-yl)-4,4,5,5-tetramethyl-1,3,2-dioxaborolane (3d)**

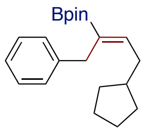


The title compound was isolated (107 mg, 82%) as colourless oil after chromatography (80:1 hexane/EtOAc). $^1H$ NMR (500 MHz, $C_6D_6$) δ 7.39 (d, *J* = 7.1 Hz, 2H), 7.21 (t, *J* = 7.7 Hz, 2H), 7.07 (t, *J* = 7.4 Hz, 1H), 6.89 (t, *J* = 7.2 Hz, 1H), 3.74 (s, 2H), 2.21 (t, *J* = 7.1 Hz, 2H), 1.82–1.72 (m, 1H), 1.68–1.59 (m, 2H), 1.51–1.42 (m, 2H), 1.41–1.31 (m, 2H), 1.07–1.00 (m, 14H). $^{13}C\{^1H\}$ NMR (126 MHz, $C_6D_6$) δ 147.8, 143.2, 129.7, 129.1, 126.5, 83.9, 40.9, 36.0, 35.5, 33.4, 26.0, 25.5. The boron-bound carbon was not detected due to quadrupolar relaxation. IR: $\nu_{max}$(nujol) 3086, 3063, 2997, 1632, 1491. HR-MS (APCI) *m/z*: calcd for $C_{21}H_{31}BNaO_2$ ([M+Na]$^+$): 349.2313; Found: 349.2309.

**(*Z*)-2-(1-Cyclohexyl-3-phenylprop-1-en-2-yl)-4,4,5,5-tetramethyl-1,3,2-dioxaborolane (3e)**

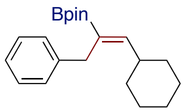


The title compound was isolated (112 mg, 86%) as colourless oil after chromatography (80:1 hexane/EtOAc). $^1H$ NMR (500 MHz, $C_6D_6$) δ 7.40 (d, *J* = 7.2 Hz, 2H), 7.21 (t, *J* = 7.6 Hz, 2H), 7.07 (t, *J* = 7.4 Hz, 1H), 6.70 (d, *J* = 9.6 Hz, 1H), 3.74 (s, 2H), 2.60–2.50 (m, 1H), 1.65–1.59 (m, 2H), 1.57–1.52 (m, 2H), 1.52–1.46 (m, 1H), 1.18–1.03 (m, 5H), 1.00 (s, 12H). $^{13}C\{^1H\}$ NMR (126 MHz, $C_6D_6$) δ 153.5, 143.4, 129.7, 129.1, 126.5, 83.9, 38.7, 35.6, 33.5, 26.9, 26.7, 25.5. The boron-bound carbon was not detected due to quadrupolar relaxation. IR: $\nu_{max}$(nujol) 3063, 3025, 2991, 1630, 1601, 1491. HR-MS (ESI) m/z: calcd for $C_{21}H_{31}BNaO_2$ ([M+Na]$^+$): 349.2313; Found: 349.2313.

**(*Z*)-4,4,5,5-Tetramethyl-2-(4-methyl-1-phenylpent-2-en-2-yl)-1,3,2-dioxaborolane (3f)**

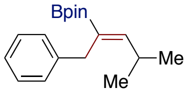


The title compound was isolated (96.2 mg, 84%) as colourless oil after chromatography (80:1 hexane/EtOAc). $^1H$ NMR (500 MHz, $C_6D_6$) δ 7.36 (d, *J* = 6.9 Hz, 2H), 7.20 (t, *J* = 7.6 Hz, 2H), 7.07 (t, *J* = 7.4 Hz, 1H), 6.63 (d, *J* = 9.7 Hz, 1H), 3.69 (s, 2H), 2.80–2.68 (m, 1H), 1.01 (s, 12H), 0.87 (d, *J* = 6.7 Hz, 6H). $^{13}C\{^1H\}$ NMR (126 MHz, $C_6D_6$) δ 155.0, 143.3, 129.7, 129.1, 126.5, 83.9, 35.4, 28.7, 25.5, 23.2. The boron-bound carbon was not detected due to quadrupolar relaxation. IR: $\nu_{max}$(nujol) 3086, 3064, 3027, 2996, 1664, 1635, 1491. HR-MS (ESI) m/z: calcd for $C_{18}H_{27}BNaO_2$ ([M+Na]$^+$): 309.1996; Found: 309.1997.

**(*Z*)-4,4,5,5-Tetramethyl-2-(1-(o-tolyl)but-2-en-2-yl)-1,3,2-dioxaborolane (3g)**

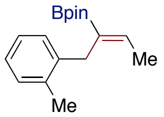


The title compound was isolated (87.1 mg, 80%) as colourless oil after chromatography (80:1 hexane/EtOAc). $^{1}$H NMR (500 MHz, $CDCl_3$) δ 7.11–7.04 (m, 4H), 6.60 (q, $J$ = 6.8 Hz, 1H), 3.45 (s, 2H), 2.33 (s, 3H), 1.72 (d, $J$ = 7.0 Hz, 3H), 1.17 (s, 12H). $^{13}$C{$^{1}$H} NMR (126 MHz, $CDCl_3$) δ 141.6, 139.4, 136.4, 129.6, 128.1, 125.5, 125.4, 83.1, 31.0, 24.6, 19.8, 14.7. The boron-bound carbon was not detected due to quadrupolar relaxation. IR: $\nu_{max}$(nujol) 2989, 1633, 1607, 1476. HR-MS (APCI) *m/z*: calcd for $C_{17}H_{25}BNaO_2$ ([M+Na]$^+$): 295.1483; Found: 295.1846.

**(*Z*)-4,4,5,5-Tetramethyl-2-(1-(m-tolyl)but-2-en-2-yl)-1,3,2-dioxaborolane (3h)**

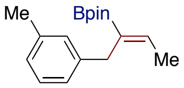


The title compound was isolated (88.2 mg, 81%) as colourless oil after chromatography (80:1 hexane/EtOAc). $^{1}$H NMR (500 MHz, $C_6D_6$) δ 7.20–7.15 (m, 2H), 7.16–7.11 (m, 3H), 6.93–6.82 (m, 2H), 3.67 (s, 2H), 2.15 (s, 3H), 1.63 (d, $J$ = 6.8 Hz, 3H), 1.02 (s, 12H). $^{13}$C{$^{1}$H} NMR (126 MHz, $C_6D_6$) δ 142.9, 142.4, 138.3, 130.6, 129.1, 127.2, 126.7, 83.8, 34.9, 25.5, 22.2, 15.4. The boron-bound carbon was not detected due to quadrupolar relaxation. IR: $\nu_{max}$(nujol) 3018, 2986, 1633, 1614, 1483. GC-MS (EI): calculated for $C_{17}H_{25}BO_2$: 272.19; found: 289.20.

**(*Z*)-4,4,5,5-Tetramethyl-2-(1-(p-tolyl)but-2-en-2-yl)-1,3,2-dioxaborolane (3i)**

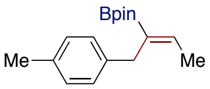


The title compound was isolated (81.7 mg, 75%) as colourless oil after chromatography (80:1 hexane/EtOAc). $^{1}$H NMR (500 MHz, $C_6D_6$) δ 7.26 (d, $J$ = 8.0 Hz, 2H), 7.02 (d, $J$ = 7.9 Hz, 2H), 6.85 (q, $J$ = 6.8 Hz, 1H), 3.66 (s, 2H), 2.13 (s, 4H), 1.63 (d, $J$ = 6.8 Hz, 3H), 1.03 (s, 13H). $^{13}$C{$^{1}$H} NMR (126 MHz, $C_6D_6$) δ 142.3, 139.9, 135.4, 129.9, 129.6, 83.8, 34.5, 25.5, 21.7, 15.4. The boron-bound carbon was not detected due to quadrupolar relaxation. IR: $\nu_{max}$(nujol) 3086, 3050, 3020, 2996, 1630, 1516, 1483. HR-MS (ESI) m/z: calcd for $C_{17}H_{25}BNaO_2$ ([M+Na]$^+$): 295.1843; Found: 295.1849.

**(*Z*)-2-(1-(4-(tert-Butyl)phenyl)but-2-en-2-yl)-4,4,5,5-tetramethyl-1,3,2-dioxaborol ane (3j)**

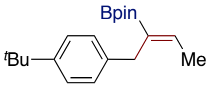


The title compound was isolated (91.8 mg, 73%) as colourless oil after chromatography (80:1 hexane/EtOAc). $^{1}$H NMR (500 MHz, $C_6D_6$) δ 7.34 (d, $J$ = 8.2 Hz, 2H), 7.29 (d, $J$ = 8.3 Hz, 2H), 6.90 (q, $J$ = 6.8 Hz, 1H), 3.72 (s, 2H), 1.65 (d, $J$ = 6.8 Hz, 3H), 1.23 (s, 9H), 1.04 (s, 12H). $^{13}$C{$^{1}$H} NMR (126 MHz, $C_6D_6$) δ 148.8, 142.5, 139.9, 129.3, 126.1, 83.8, 35.0, 34.4, 32.3, 25.5, 15.4. The boron-bound carbon was not detected due to quadrupolar relaxation. IR: $\nu_{max}$(nujol) 3088, 3050, 3023, 2991, 1634, 1516, 1481. HR-MS (ESI) m/z: calcd for $C_{20}H_{31}BNaO_2$ ([M+Na]$^+$): 337.2313; Found: 337.2315.

**(*Z*)-2-(1-(4-Fluorophenyl)but-2-en-2-yl)-4,4,5,5-tetramethyl-1,3,2-dioxaborolane (3m)**

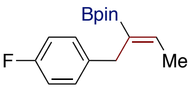


The title compound was isolated (69.6 mg, 63%) as colourless oil after chromatography (80:1 hexane/EtOAc). After purification,

**2m**:**3m** = 18:100. $^{1}$H NMR (500 MHz, $C_6D_6$) δ 7.12–7.04 (m, 2H), 6.86–6.78 (m, 4H), 3.52 (s, 2H), 1.56 (d, *J* = 6.9 Hz, 3H), 1.01 (s, 12H). $^{13}$C NMR (126 MHz, $C_6D_6$) δ 162.3(d, *J* = 243.0 Hz), 142.5, 138.6 (d, *J* = 3.2 Hz), 131.0 (d, *J* = 7.5 Hz), 115.7 (d, *J* = 21.0 Hz), 83.9, 34.0, 25.5, 15.3. The boron-bound carbon was not detected due to quadrupolar relaxation. IR: $\nu_{max}$(nujol) 3035, 2989, 1632, 1603, 1506, 1492. GC-MS (EI): calculated for $C_{16}H_{22}BFO_2$: 276.17; Found: 276.15.

**(*Z*)-Trimethyl(4-(2-(4,4,5,5-tetramethyl-1,3,2-dioxaborolan-2-yl)but-2-en-1-yl)phenyl)silane (3q)**

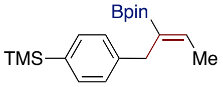


The title compound was isolated (108 mg, 82%) as colourless oil after chromatography (80:1 hexane/EtOAc). $^{1}$H NMR (500 MHz, $C_6D_6$) δ 7.47 (d, *J* = 8.0 Hz, 2H), 7.39 (d, *J* = 7.8 Hz, 2H), 6.90 (q, *J* = 6.9 Hz, 1H), 3.71 (s, 2H), 1.63 (d, *J* = 6.8 Hz, 3H), 1.03 (s, 12H), 0.22 (s, 9H). $^{13}$C{$^{1}$H} NMR (126 MHz, $C_6D_6$) δ 143.7, 142.8, 137.4, 134.4, 129.2, 83.9, 34.9, 25.5, 15.5, -0.3. The boron-bound carbon was not detected due to quadrupolar relaxation. IR: $\nu_{max}$(nujol) 3063, 3025, 2991, 1630, 1601, 1492. HR-MS (APCI) m/z: calcd for $C_{19}H_{31}BO_2Si$ ($[M]^+$): 330.2185; Found: 330.2172.

**(*Z*)-4,4,5,5-tetramethyl-2-(4-(2-(4,4,5,5-tetramethyl-1,3,2-dioxaborolan-2-yl)but-2-en-1-yl)phenyl)-1,3,2-dioxaborolane (3s)**

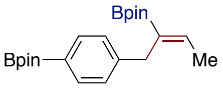


The title compound was isolated (123 mg, 80%) as colourless oil after chromatography (30:1 hexane/EtOAc). $^{1}$H NMR (500 MHz, $C_6D_6$) δ 8.12 (d, *J* = 8.1 Hz, 2H), 7.37 (d, *J* = 8.3 Hz, 2H), 6.79 (qt, *J* = 6.9, 1.1 Hz, 1H), 3.61 (s, 2H), 1.53 (d, *J* = 6.9 Hz, 3H), 1.08 (s, 12H), 0.96 (s, 12H). $^{13}$C{$^{1}$H} NMR (126 MHz, $C_6D_6$) δ 146.6, 142.6, 136.2, 129.3, 84.2, 83.9, 35.2, 25.7, 25.5, 15.4. IR: $\nu_{max}$(nujol) 2989, 1630, 1610, 1514, 1476. The boron-bound carbon was not detected due to quadrupolar relaxation. GC-MS (EI): calculated for $C_{22}H_{34}B_2O_4$: 384.26; found: 384.25.

**(*Z*)-4,4,5,5-tetramethyl-2-(1-(4-(methylthio)phenyl)but-2-en-2-yl)-1,3,2-dioxaborolane (3t)**

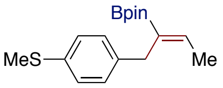


The title compound was isolated (87.6 mg, 72%) as colourless oil after chromatography (60:1 hexane/EtOAc). $^{1}$H NMR (500 MHz, $C_6D_6$) δ 7.21 (d, *J* = 8.2 Hz, 2H), 7.17 (d, *J* = 8.4 Hz, 2H), 6.87 (q, *J* = 6.8 Hz, 1H), 3.61 (s, 2H), 2.02 (s, 3H), 1.60 (d, *J* = 6.9 Hz, 3H), 1.03 (s, 12H). $^{13}$C{$^{1}$H} NMR (126 MHz, $C_6D_6$) δ 142.6, 140.1, 136.4, 130.2, 128.2, 83.9, 34.4, 25.5, 16.7, 15.4. The boron-bound carbon was not detected due to quadrupolar relaxation. IR: $\nu_{max}$(nujol) 3071, 3018, 2994, 1632, 1494. HR-MS (APCI) *m/z*: calcd for $C_{17}H_{26}BO_2S$ ($[M+H]^+$): 305.1744; Found: 305.1745.

**(*Z*)-4,4,5,5-Tetramethyl-2-(1-(thiophen-3-yl)but-2-en-2-yl)-1,3,2-dioxaborolane (3v)**

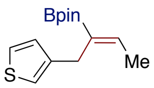


The title compound was isolated (69.7 mg, 66%,) as colourless oil after chromatography (80:1 hexane/EtOAc). $^{1}$H NMR (500 MHz, $C_6D_6$) δ 6.98 – 6.91 (m, 2H), 6.86–6.83 (m, 1H), 6.80 (q, *J* = 6.8 Hz, 1H), 3.59

(s, 2H), 1.57 (d, $J$ = 6.9 Hz, 3H), 1.03 (s, 13H). $^{13}C\{^{1}H\}$ NMR (126 MHz, $C_6D_6$) δ 143.0, δ 142.4, 129.5, 125.8, 121.1, 83.9, 29.7, 25.5, 15.1. The boron-bound carbon was not detected due to quadrupolar relaxation. IR: $\nu_{max}$(nujol) 2989, 1632, 1531, 1476. HR-MS (APCI) m/z: calcd for $C_{14}H_{22}BO_2S$ ([M+H]$^+$): 265.1431; Found: 265.1434.

### (*Z*)-2-(dec-2-en-3-yl)-4,4,5,5-tetramethyl-1,3,2-dioxaborolane (3x)

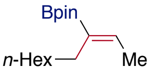


The title compound was isolated (77.7 mg, 73%, mixture of **2x** and **3x**) as colourless oil after chromatography (80:1 hexane/EtOAc).

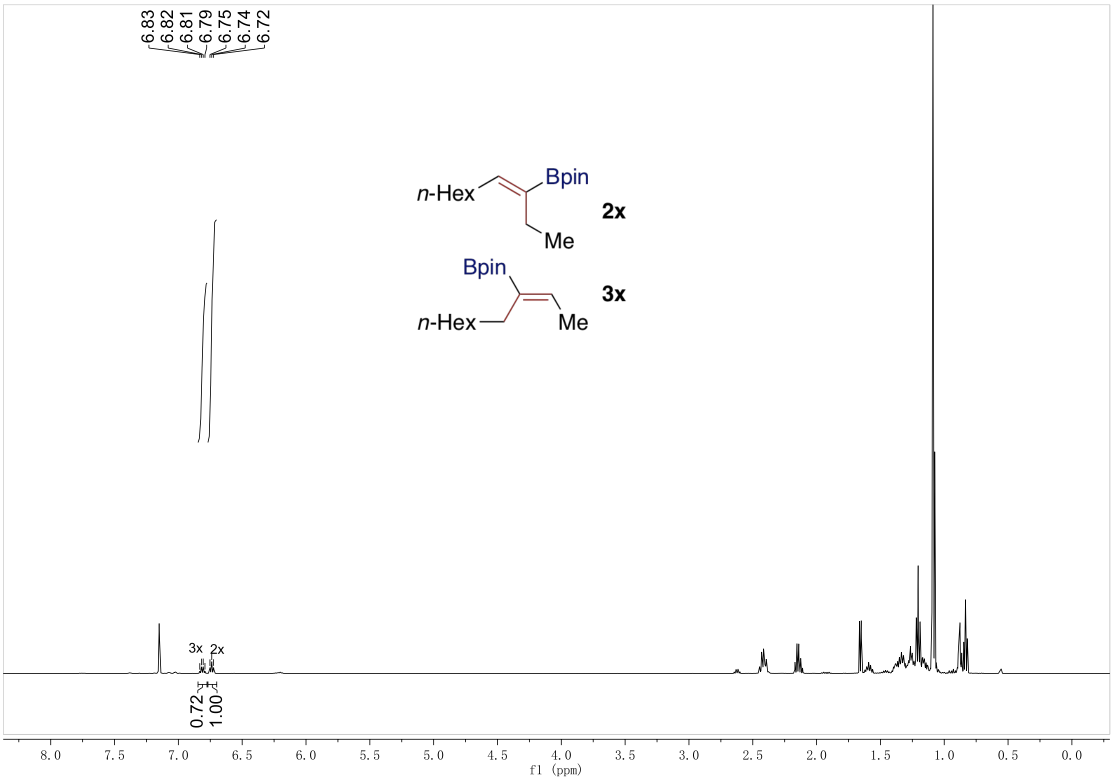


### (*Z*)-2-(1-cyclohexylbut-2-en-2-yl)-4,4,5,5-tetramethyl-1,3,2-dioxaborolane (3y)

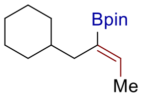


The title compound was isolated (82.4 mg, 78%, mixture of **2y** and **3y**) as colourless oil after chromatography (80:1 hexane/EtOAc).

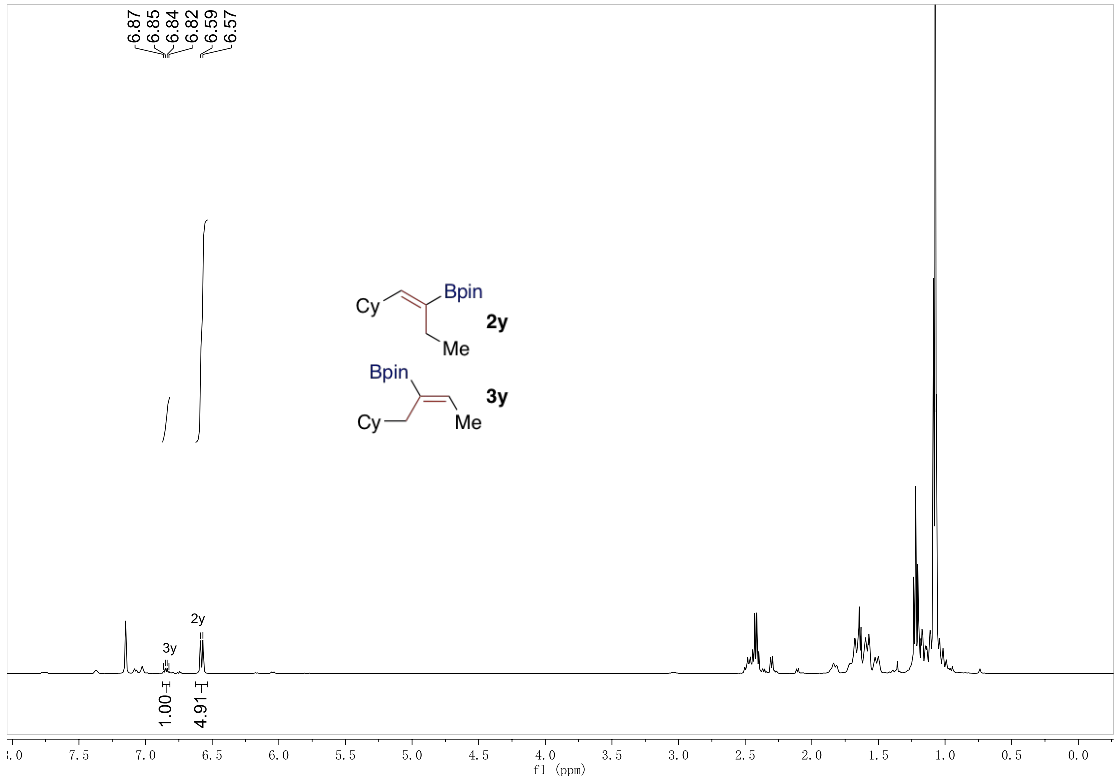


## IV. Procedures for Gram-Scale Reaction

(**A**) $Co(acac)_2$ (20.6 mg, 80.0 μmol) and dppf (44.4 mg, 80.0 μmol) weighed in air and added to a 50 mL round Schlenk flask containing a stirring bar. The air in the flask was replaced with $N_2$ by a sequential vacuum and $N_2$-refill thrice. Then degassed toluene (12 mL) were added and the mixture was stirred for 5 minutes. Allene **1m** (1.19 g, 8.00 mmol) and HBpin (1.13 g, 8.80 mmol) were injected successively into the mixture using a syringe. The reaction mixture was stirred at room temperature for 12 h. The crude mixture was purified by column chromatography silica gel with a mixture of hexane and ethyl acetate (80:1), yielding the product **2m** (1.81 g, 82%) as a colorless oil.

(B) $Co(acac)_2$ (20.6 mg, 80.0 μmol) and xantphos (46.3 mg, 80.0 μmol) weighed in air and added to a 50 mL round Schlenk flask containing a stirring bar. The air in the flask was replaced with $N_2$ by a sequential vacuum and $N_2$-refill thrice. Then degassed toluene (12 mL) were added and the mixture was stirred for 5 minutes. Allene **1f** (1.27 g, 8.00 mmol) and HBpin (1.13 g, 8.80 mmol) were injected successively into the mixture using a syringe. The reaction mixture was stirred at room temperature for 12 h. The crude mixture was purified by column chromatography silica gel with a mixture of hexane and ethyl acetate (80:1), yielding the product **3f** (1.81 g, 82%) as a colorless oil.

## V. Procedures of the Transformations of (*Z*)-Alkenylboronate 2k

### Procedure of Suzuki-Miyaura Coupling[1]

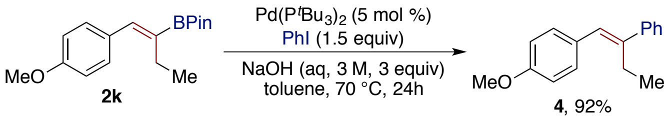


To a 25 mL Schlenk flask charged with a magnetic stirring bar, were added the (*Z*)-alkenylboronate **2k** (115 mg, 0.400 mmol), PhI (122 mg, 0.600 mmol), Pd(P$^t$Bu$_3$)$_2$ (10.2 mg, 20.0 µmol), 2 mL of dry THF, and 0.4 mL of NaOH (3M, 1.2 mmol) aqueous solution. The flask was sealed with a PTFE septum, and allowed to react at 70 °C for 24 h. The crude residue of the reaction was purified by column chromatography on silica gel with a mixture of hexane and ethyl acetate (80:1), yielding the product **4** (87.7 mg, 92%) as a colorless oil. $^1$H NMR (400 MHz, $CDCl_3$) δ 7.52 7.44 (m, 2H), 7.43–7.34 (m, 2H), 7.34–7.25 (m, 3H), 6.98–6.89 (m, 2H), 6.66 (s, 1H), 3.85 (s, 3H), 2.77 (q, *J* = 7.5 Hz, 2H), 1.10 (t, *J* = 7.5 Hz, 3H). $^{13}C\{^1H\}$ NMR (101 MHz, $CDCl_3$) δ 158.3, 143.1, 142.9, 130.9, 129.9, 128.3, 127.1, 126.9, 126.6, 113.7, 55.2, 23.2, 13.5. IR: $\nu_{max}$(nujol) 3060, 3024, 2998, 1606, 1572, 1537, 1514, 1476. GC-MS (EI): calcd for $C_{17}H_{18}O$: 238.14; Found: 238.20.

### Procedure of Deborohydrogenation[2]

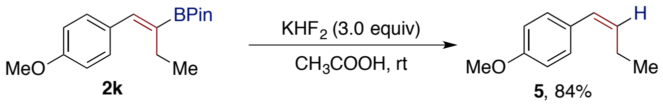


To a solution of **2k** (115 mg, 0.400 mmol) in HOAc (5 mL) was added $KHF_2$ (93.7 mg, 1.200 mmol) under air, the reaction mixture was stirred at room temperature for 6 h. Then the reaction was quenched with saturated $K_2CO_3$ solution, and resulting mixture was extracted with diethyl ether. The combined organic layers were washed with brine, and dried over anhydrous sodium sulfate. The volatiles were removed under reduced pressure and the residue was purified by column chromatography on silica gel with a mixture of hexane and ethyl acetate (80:1), yielding the product **5** (54.5 mg, 84%) as a colourless oil. $^1$H NMR (400 MHz, $CDCl_3$) δ 7.32–7.26 (m, 2H), 6.96–6.91 (m, 2H), 6.39 (dt, *J* = 11.5, 1.9 Hz, 1H), 5.63 (dt, *J* = 11.6, 7.2 Hz, 1H), 3.86 (s, 3H), 2.41 (pd, *J* = 7.4, 1.8 Hz, 2H), 1.14 (t, *J* = 7.5 Hz, 3H). $^{13}C\{^1H\}$ NMR (101 MHz, $CDCl_3$) δ 158.2, 133.2, 130.5, 129.9, 127.7, 113.6, 55.2, 22.0, 14.6. IR: $\nu_{max}$(nujol) 3093, 3057, 3004, 2982, 1614, 1514, 1466, 1446. GC-MS (EI): calculated for $C_{11}H_{14}O$: 162.10; found: 162.15.

### Procedure of Iodination[3]

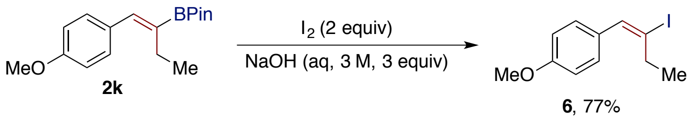


To a solution of **2k** (115 mg, 0.400 mmol) in THF (2.0 mL) was added 0.400 mL of NaOH (aq. 3M, 1.200 mmol). After strring for 10 minutes at room temperature, 0.400

mL of solution of $I_2$ (2M, 0.800 mmol) in THF was added into the reaction mixture, and the resulting mixture was stirred for another 1 h. The mixture was then quenched with saturated aqueous $Na_2S_2O_4$ (5.0 mL) and extracted with diethyl ether for three times. The combined organic layers were washed with saturated aqueous $NHCO_3$ solution and brine, then dried over $Na_2SO_4$. The volatiles were removed under reduced pressure and the residue was purified by column chromatography on silica gel with a mixture of hexane and ethyl acetate (80:1), yielding the product **6** (88.7mg, 77%) as a colorless oil. $^1$H NMR (400 MHz, $C_6D_6$) δ 7.23 (s, 1H), 6.90–6.85 (m, 2H), 6.68–6.60 (m, 2H), 3.25 (s, 3H), 2.43 (qd, $J$ = 7.3, 1.0 Hz, 2H), 1.01 (t, $J$ = 7.3 Hz, 3H). $^{13}$C{$^1$H} NMR (101 MHz, $C_6D_6$) δ 160.0, 140.7, 131.3, 130.2, 114.8, 110.2, 55.4, 34.2, 15.6. IR: $\nu_{max}$(nujol) 2997, 1610, 1574, 1513. GC-MS (EI): calculated for $C_{11}H_{13}IO$: 288.00; found: 288.05.

## VI. The Procedure for the Co-catalyzed Hydrosilylation of 1,3-Disubstituted Allene with dppf as Ligand

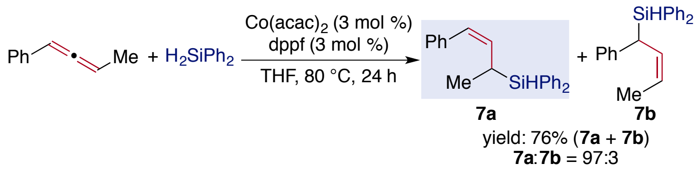


In an Argon-filled glovebox, Co(acac)$_2$ (3.1 mg, 12.0 μmol), dppf (6.7 mg,12.0 μmol), allene **1a** (52.1 mg, 0.400 mmol), $H_2SiPh_2$ (81.1mg, 0.440 mmol), and toluene (1 mL) were added to a 4-mL screw-capped vial containing a magnetic stirring bar. The vial was sealed with a cap containing a PTFE septum, removed from the dry box and heated at 80 °C for 24 h. The crude product was purified by column chromatography on silica gel with hexane as eluent, yielding the allylsilanes **7a** and **7b** (95.6 mg, 76%, **7a**:**7b** = 97:3) as a colorless oil.

## VII. General Procedure for the Co-catalyzed Hydrosilylation of 1,3-Disubstituted Allene with Xantphos as Ligand

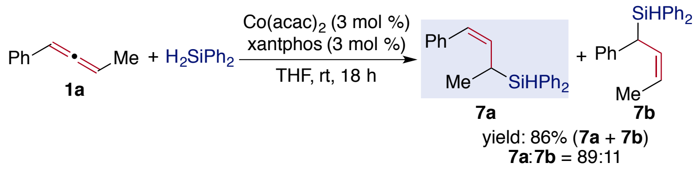


In an Argon-filled glovebox, Co(acac)$_2$ (3.1 mg, 12.0 μmol), xantphos (6.9 mg,12.0 μmol), allene **1a** (52.1 mg, 0.400 mmol), $H_2SiPh_2$ (81.1mg, 0.440 mmol), and toluene (1 mL) were added to a 4-mL screw-capped vial containing a magnetic stirring bar. The vial was sealed with a cap containing a PTFE septum, removed from the dry box and stirred for 18 h. The crude product was purified by column chromatography on silica gel with hexane as eluent, yielding product **7a** and **7b** (108 mg, 86%, **7a**:**7b** = 89:11) as a colorless oil. **7a**: $^1$H NMR (500 MHz, $CDCl_3$) δ 7.70–7.63 (m, 4H), 7.49–7.38 (m, 6H), 7.34–7.28 (m, 4H), 7.25–7.19 (m, 1H), 6.41 (dd, $J$ = 15.9, 7.5 Hz,

1H), 6.30 (d, *J* = 16.0 Hz, 1H), 4.88 (d, *J* = 2.8 Hz, 1H), 2.56 (p, *J* = 7.2 Hz, 1H), 1.38 (d, *J* = 7.2 Hz, 3H).

### Reactions with Other Boron Source

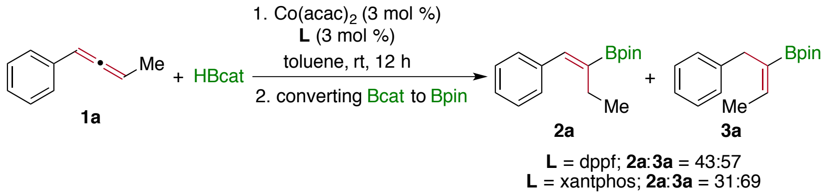


The reaction of **1a** with HBcat is not as selective as the reaction with HBpin. Because alkenylBcat products are too unstable to isolate, we converted alkenylBcat to Bpin esters for the convenience of isolation. As these reactions proceed through a Co-Boryl intermediate, the boryl group on the cobalt catalyst does influence the regioselectivity for these allene hydroboration reactions. Overall, the regioselectivity of these reactions are controlled by the synergy among the allene substrate, ligand, and the boryl group on the cobalt catalyst.

The reaction of **1a** with 9-BBN is even less selective and this might be due to the un-catalyzed background reactions of allene **1a** with active 9-BBN.

## VIII. Procedures of Deuterium-labeling experiments

### (A) Procedure of Deuterium-Labeling Experiment with dppf as Ligand

In an Argon-filled glovebox, $Co(acac)_2$ (3.1 mg, 12.0 μmol), dppf (6.7 mg,12.0 μmol), allene **1q** (102 mg, 0.400 mmol), DBpin (56.3mg, 0.440 mmol), and toluene (1 mL) were added to a 4-mL screw-capped vial containing a magnetic stirring bar. The vial was sealed with a cap containing a PTFE septum and removed from the dry box. The reaction mixture was stirred at room temperature for 12 h, and the crude product was purified by column chromatography on silica gel with a mixture of hexane and ethyl acetate (10:1) as eluent, yielding the product **2q-*d*$_1$** (128 mg, 83%) as a colorless oil.

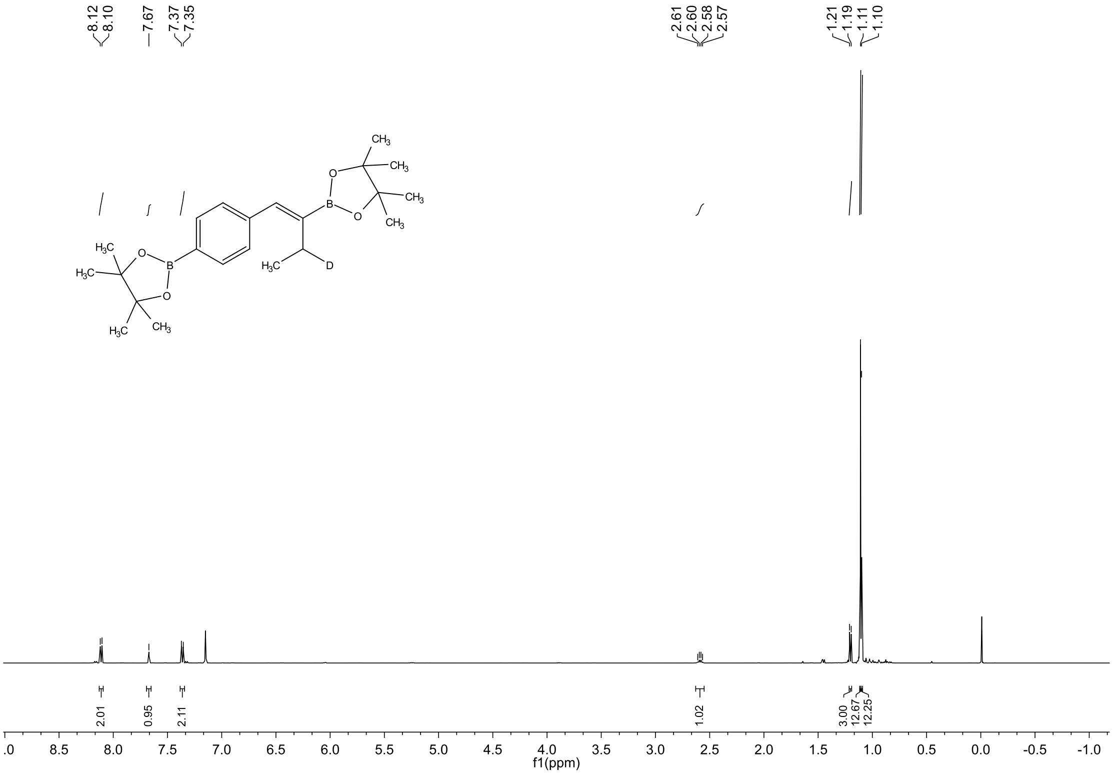


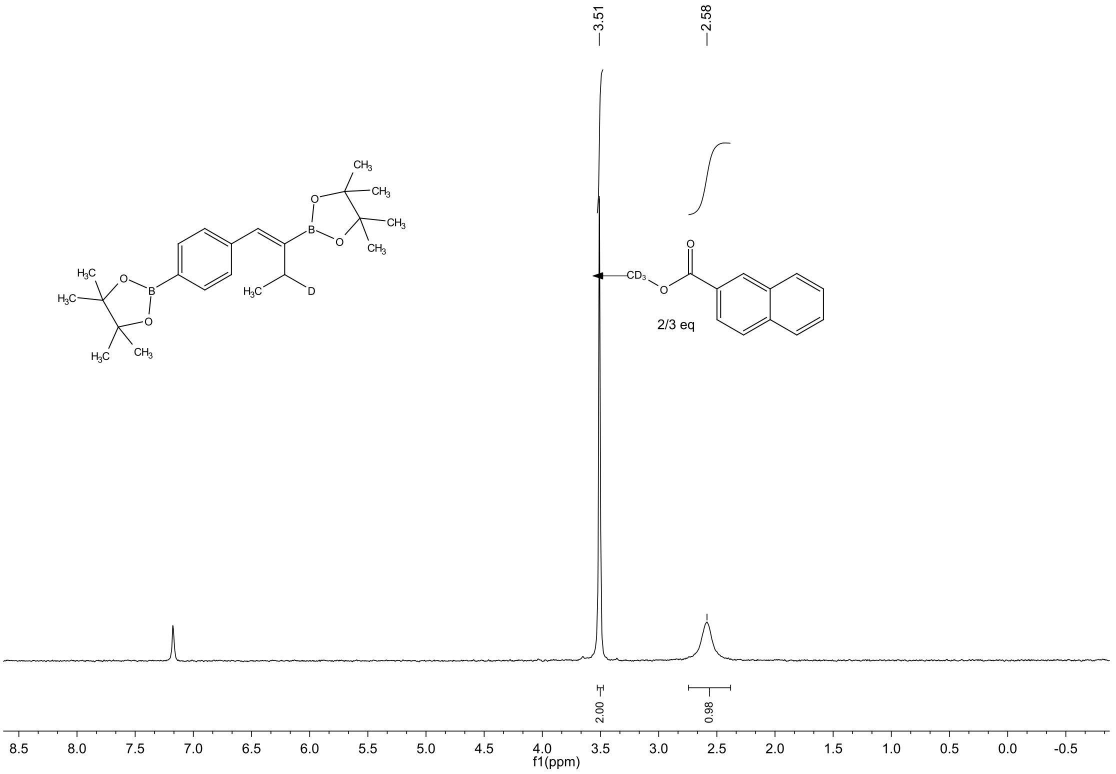


## (B) Procedure of Deuterium-labeling experiment with Xantphos as Ligand

In an Argon-filled glovebox, $Co(acac)_2$ (3.1 mg, 12.0 μmol), xantphos (6.9 mg,12.0 μmol), allene **1q** (102 mg, 0.400 mmol), DBpin (56.3mg, 0.440 mmol), and toluene (1 mL) were added to a 4-mL screw-capped vial containing a magnetic stirring bar. The vial was sealed with a cap containing a PTFE septum and removed from the dry box.

The reaction mixture was stirred at room temperature for 12 h, and the crude product was purified by column chromatography on silica gel with a mixture of hexane and ethyl acetate (10:1) as eluent, yielding product **3q-*d*₁** (132 mg, 86%) as a colorless oil.

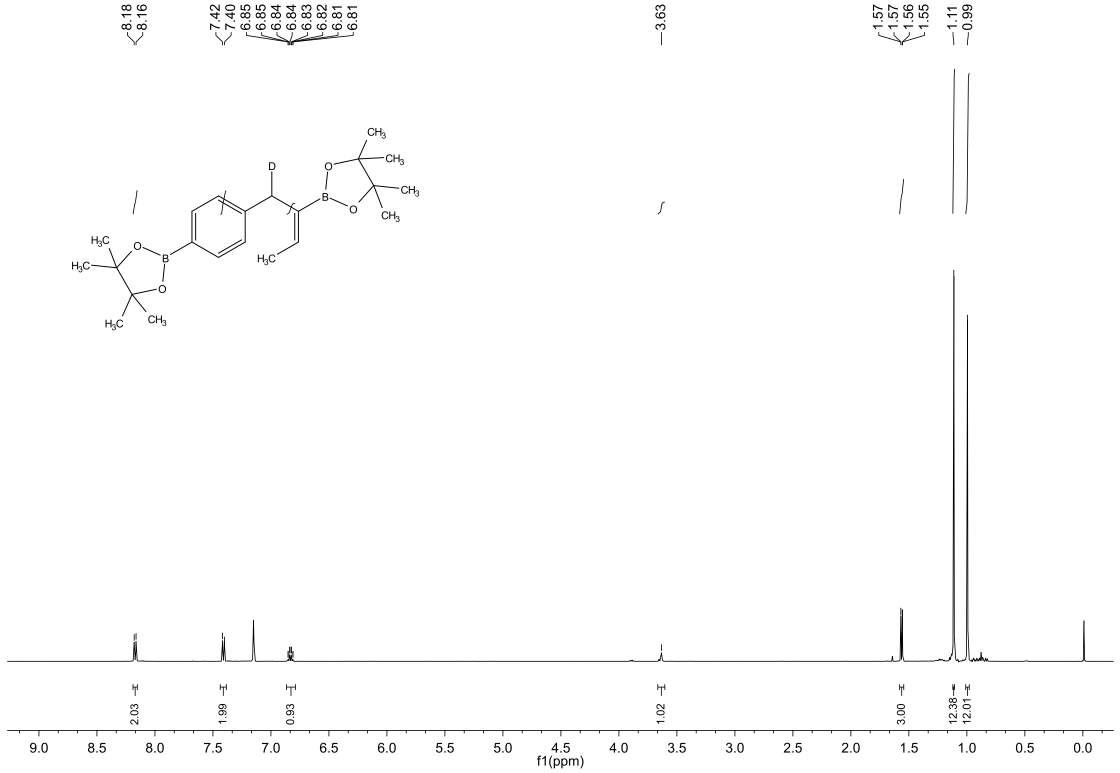


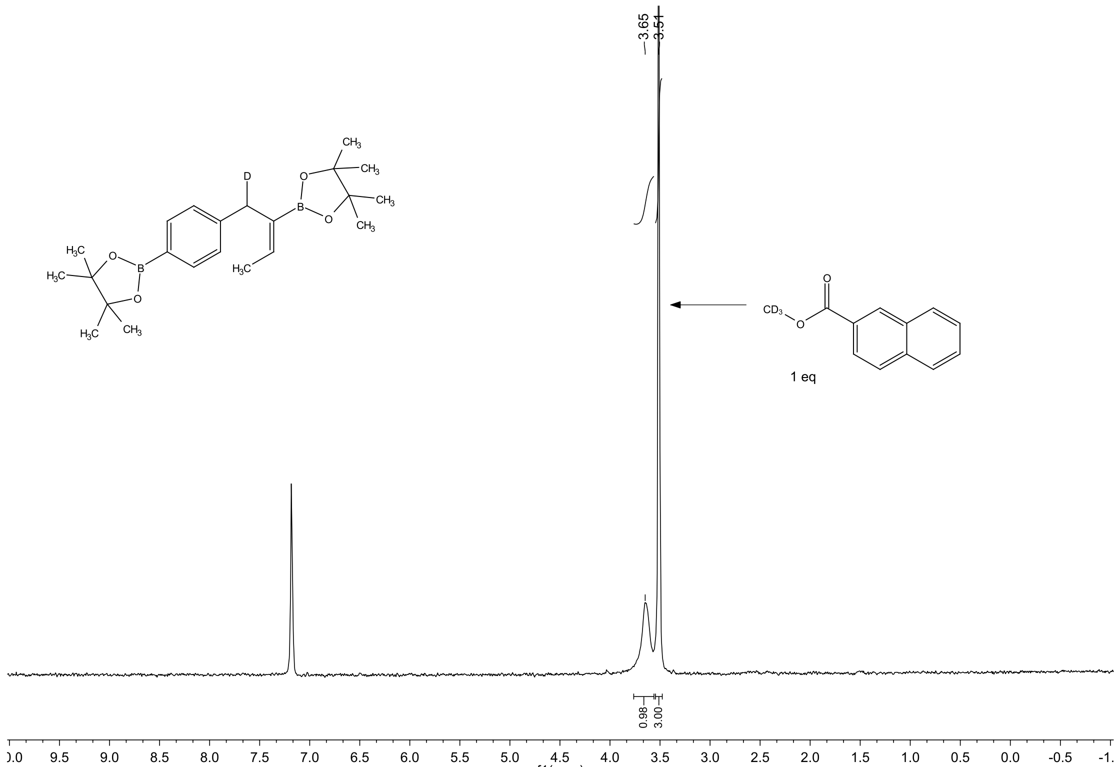

## IX. General procedure of the Preparation of Allene Substrates[4]

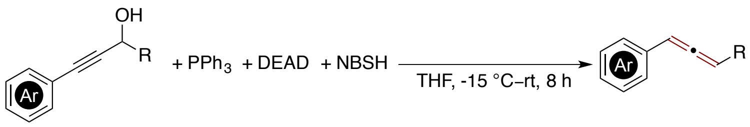


To a solution of triphenylphosphine (3.28g, 12.50 mmol) in THF at -15 °C was added 5.5 mL of diethyl azodicarboxylate solution (DEAD, 2.2 M in toluene, 12.00 mmol) dropwise over 1 minute. The reaction mixture was stirred at -15 °C for additional 10 minutes, then propargyl alcohol solution (10.00 mmol in THF) was added dropwise over 10 min. After 5 minutes, 2-nitrobenxylsulfonylhydrazine (NBSH) in THF (2.33g, 12.50 mmol) was added into the reaction mixture. The reaction was stirred at -15 °C for 40 minutes and then allowed to warm to room temperature for 6.5 h. After that, the reaction was quenched with saturated aqueous solution of $NH_4Cl$, and extracted with diethyl ether thrice. The combined organic layers were washed with saturated aqueous $NaHCO_3$ solution and brine, then dried over $Na_2SO_4$. All the volatiles were removed under reduced pressure and the residue was purified by column chromatography on silica gel.

**Buta-1,2-dien-1-ylbenzene (1a)**

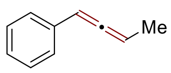


$^1$H NMR (500 MHz, $CDCl_3$) δ 7.34–7.27 (m, 4H), 7.22–7.15 (m, 1H), 6.10 (dq, *J* = 6.4, 3.2 Hz, 1H), 5.54 (p, *J* = 7.0 Hz, 1H), 1.79 (dd, *J* = 7.1, 3.2 Hz, 3H). $^{13}$C{$^1$H} NMR (126 MHz, $CDCl_3$) δ 206.0, 135.0, 128.5, 126.6, 94.0, 89.6, 14.1. GC-MS (EI): calculated for $C_{10}H_{10}$: 130.08; found: 130.10. The NMR data of this compound was in accordance with reported literature.[5]

**Hexa-1,2-dien-1-ylbenzene (1b)**

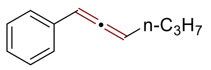


$^1$H NMR (400 MHz, $CDCl_3$) δ 7.29 (d, *J* = 4.4 Hz, 4H), 7.18 (h, *J* = 3.9 Hz, 1H), 6.12 (dt, *J* = 6.2, 3.0 Hz, 1H), 5.57 (q, *J* = 6.7 Hz, 1H), 2.17–2.07 (m, 2H), 1.51 (dt, *J* = 14.7, 7.4 Hz, 2H), 0.97 (t, *J* = 7.4 Hz, 3H). $^{13}$C{$^1$H} NMR (101 MHz, $CDCl_3$) δ 205.2, 135.1, 128.5, 126.6(two peaks overlap), 94.9, 94.5, 30.8, 22.4, 13.7. GC-MS (EI): calculated for $C_{12}H_{14}$: 158.11; found: 158.15. The NMR data of this compound was in accordance with reported literature.[6]

**Octa-1,2-dien-1-ylbenzene (1c)**

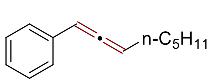


$^1$H NMR (400 MHz, $CDCl_3$) δ 7.32–7.28 (m, 4H), 7.22–7.16 (m, 1H), 6.13 (dt, *J* = 6.2, 3.0 Hz, 1H), 5.57 (q, *J* = 6.7 Hz, 1H), 2.17–2.10 (m, 2H), 1.54–1.46 (m, 2H), 1.39–1.30 (m, 4H), 0.90 (t, *J* = 7.2 Hz, 3H). $^{13}$C{$^1$H} NMR (101 MHz, $CDCl_3$) δ 205.2, 135.2, 128.5, 126.6 (two peaks overlap), 95.1, 94.5, 31.4, 28.9, 28.7, 22.5, 14.1. GC-MS (EI): calculated for $C_{14}H_{18}$: 186.14; found: 186.15. The NMR data of this compound was in accordance with reported literature.[7]

**(4-Cyclopentylbuta-1,2-dien-1-yl)benzene (1d)**

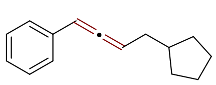

$^1$H NMR (400 MHz, $CDCl_3$) δ 7.32–7.27 (m, 4H), 7.21–7.15 (m, 1H), 6.11 (dt, *J* = 5.9, 2.9 Hz, 1H), 5.56 (q, *J* = 6.8 Hz, 1H), 2.15 (ddd, *J* = 9.9, 7.0, 2.9 Hz, 2H), 1.98 (hept, *J* = 7.6 Hz, 1H), 1.89–1.75 (m, 2H), 1.65–1.57 (m, 2H), 1.56–1.48 (m, 2H), 1.26–1.15 (m, 2H). $^{13}C\{^1H\}$ NMR (101 MHz, $CDCl_3$) δ 205.5, 135.2, 128.5, 126.6, 126.5, 94.4, 94.1, 39.8, 35.3, 32.5, 32.4, 25.3(two peaks overlap). GC-MS (EI): calculated for $C_{15}H_{18}$: 198.14; found: 198.10. The NMR data of this compound was in accordance with reported literature.[8]

**(3-Cyclohexylpropa-1,2-dien-1-yl)benzene (1e)**

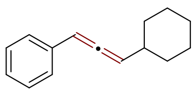

$^1$H NMR (400 MHz, $CDCl_3$) δ 7.33–7.29 (m, 4H), δ 7.23–7.17 (m, 1H), 6.18 (dd, *J* = 6.4, 3.0 Hz, 1H), 5.59 (t, *J* = 6.2 Hz, 1H), 2.20–2.11(m, 1H), 1.91–1.82 (m, 2H), 1.78–1.74 (m, 2H), 1.70–1.63 (m, 1H), 1.34–1.17 (m, 5H). $^{13}C\{^1H\}$ NMR (101 MHz, $CDCl_3$) δ 204.1, 135.2, 128.5, 126.6, 126.4, 101.1, 95.4, 37.6, 33.2, 33.1, 26.1(two peaks overlap), 26.0. GC-MS (EI): calculated for $C_{15}H_{18}$: 198.14; found: 198.15. The NMR data of this compound was in accordance with reported literature.[9]

**(4-Methylpenta-1,2-dien-1-yl)benzene (1f)**

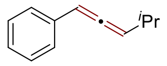


$^1$H NMR (400 MHz, $CDCl_3$) δ 7.31–7.27 (m, 4H), 7.21–7.16 (m, 1H), 6.18 (dd, *J* = 6.4, 3.1 Hz, 1H), 5.60 (t, *J* = 6.1 Hz, 1H), 2.50–2.41 (m, 1H), 1.10 (dd, *J* = 6.8, 3.1 Hz, 6H). $^{13}C\{^1H\}$ NMR (101 MHz, $CDCl_3$) δ 203.6, 135.2, 128.5, 126.6, 126.5, 102.5, 95.7, 28.4, 22.6, 22.5. GC-MS (EI): calculated for $C_{12}H_{14}$: 158.11; found: 158.15. The NMR data of this compound was in accordance with reported literature.[10]

**1-(Buta-1,2-dien-1-yl)-2-methylbenzene (1g)**

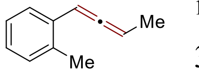


$^1$H NMR (400 MHz, CDCl3) δ 7.38 (d, J = 7.5 Hz, 1H), 7.24–7.05 (m, 3H), 6.33–6.25 (m, 1H), 5.52 (p, J = 6.8 Hz, 1H), 2.38 (s, 3H), 1.85–1.77 (m, 3H). $^{13}C\{^1H\}$ NMR (101 MHz, CDCl3) δ 206.8, 134.9, 133.2, 130.4, 127.2, 126.6, 126.0, 91.3, 88.56, 19.8, 14.2. GC-MS (EI): calculated for $C_{12}H_{12}O_2$: 144.09, found: 144.15. The NMR data of this compound was in accordance with reported literature.[6]

**1-(Buta-1,2-dien-1-yl)-3-methylbenzene (1h)**

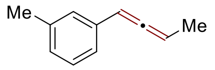


$^1$H NMR (400 MHz, $CDCl_3$) δ 7.19 (t, *J* = 7.5 Hz, 1H), 7.12–7.07 (m, 2H), 7.00 (d, *J* = 7.4 Hz, 1H), 6.07 (dq, *J* = 6.4, 3.2 Hz, 1H), 5.53 (p, *J* = 7.0 Hz, 1H), 2.34 (s, 3H), 1.79 (dd, *J* = 7.1, 3.2 Hz, 3H). $^{13}C\{^1H\}$ NMR (101 MHz, $CDCl_3$) δ 206.0, 138.1, 134.9, 128.4, 127.5, 127.3, 123.8, 94.0, 89.4, 21.4, 14.1. GC-MS (EI): calculated for $C_{11}H_{12}$: 144.09; found: 144.15. The NMR data of this compound was in accordance with reported literature.[6]

**1-(Buta-1,2-dien-1-yl)-4-methylbenzene (1i)**

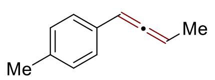


$^1$H NMR (400 MHz, $CDCl_3$) δ 7.19 (d, *J* = 8.1 Hz, 2H), 7.11 (d, *J* = 7.9 Hz, 2H), 6.07 (dq, *J* = 6.4, 3.2 Hz, 1H), 5.52 (p, *J* = 6.9 Hz, 1H),

2.33 (s, 3H), 1.78 (dd, *J* = 7.1, 3.2 Hz, 3H). $^{13}C\{^1H\}$ NMR (101 MHz, $CDCl_3$) δ 205.7, 136.4, 132.1, 129.3, 126.5, 93.8, 89.4, 21.2, 14.2. GC-MS (EI): calculated for $C_{11}H_{12}$: 144.09; found: 144.15. The NMR data of this compound was in accordance with reported literature.[6]

**1-(Buta-1,2-dien-1-yl)-4-(tert-butyl)benzene (1j)**

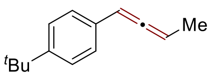


$^1H$ NMR (400 MHz, $CDCl_3$) δ 7.35–7.30 (m, 2H), 7.25–7.20 (m, 2H), 6.08 (dq, *J* = 6.4, 3.2 Hz, 1H), 5.51 (p, *J* = 7.0 Hz, 1H), 1.77 (dd, *J* = 7.1, 3.2 Hz, 3H), 1.31 (s, 9H). $^{13}C\{^1H\}$ NMR (101 MHz, $CDCl_3$) δ 205.9, 149.7, 132.1, 126.3, 125.5, 93.6, 89.4, 34.5, 31.3, 14.2. GC-MS (EI): calculated for $C_{14}H_{18}$: 186.14; found: 186.15.

**1-(Buta-1,2-dien-1-yl)-4-methoxybenzene (1k)**

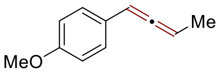


$^1H$ NMR (400 MHz, $CDCl_3$) δ 7.26–7.17 (m, 2H), 6.89–6.80 (m, 2H), 6.06 (dq, *J* = 6.4, 3.2 Hz, 1H), 5.51 (p, *J* = 7.0 Hz, 1H), 3.80 (s, 3H), 1.77 (dd, *J* = 7.1, 3.2 Hz, 3H). $^{13}C\{^1H\}$ NMR (101 MHz, $CDCl_3$) δ 205.4, 158.6, 127.7, 127.4, 114.1, 93.4, 89.5, 55.3, 14.3. GC-MS (EI): calculated for $C_{11}H_{12}O$: 160.09; found: 160.15. The NMR data of this compound was in accordance with reported literature.[11]

**1-(Buta-1,2-dien-1-yl)-4-(trifluoromethyl)benzene (1l)**

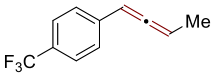


$^1H$ NMR (400 MHz, $CDCl_3$) δ 7.53 (d, *J* = 8.0 Hz, 2H), 7.37 (d, *J* = 8.0 Hz, 2H), 6.12 (dq, *J* = 6.4, 3.2 Hz, 1H), 5.60 (p, *J* = 6.9 Hz, 1H), 1.81 (dd, *J* = 7.2, 3.2 Hz, 3H). $^{13}C\{^1H\}$ NMR (101 MHz, $CDCl_3$) δ 206.9, 139.0, 128.5 (q, *J* = 32.3 Hz), 126.7, 125.4 (q, *J* = 3.9 Hz), 124.3 (q, *J* = 272.7 Hz), 93.2, 90.2, 13.8. $^{19}F\{^1H\}$ NMR (377 MHz, $CDCl_3$) δ -62.4. GC-MS (EI): calculated for $C_{11}H_9F_3$: 198.07; found: 198.05. The NMR data of this compound was in accordance with reported literature.[6]

**1-(Buta-1,2-dien-1-yl)-4-fluorobenzene (1m)**

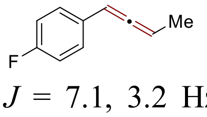


$^1H$ NMR (400 MHz, $CDCl_3$) δ 7.28–7.19 (m, 3H), 7.02–6.95 (m, 2H), 6.06 (dq, *J* = 6.4, 3.2 Hz, 1H), 5.54 (p, *J* = 7.1 Hz, 1H), 1.78 (dd, *J* = 7.1, 3.2 Hz, 3H). $^{13}C\{^1H\}$ NMR (101 MHz, $CDCl_3$) δ 205.8(d, *J* = 2.3 Hz), 161.8(d, *J* = 246.3 Hz), 131.0(d, *J* = 3.3 Hz), 128.0(d, *J* = 8.0), 115.4(d, *J* = 21.9), 93.0, 89.8, 14.1. $^{19}F\{^1H\}$ NMR (377 MHz, $CDCl_3$) δ -116.0. GC-MS (EI): calculated for $C_{10}H_9F$: 148.07; found: 148.10. The NMR data of this compound was in accordance with reported literature.[6]

**1-(Buta-1,2-dien-1-yl)-4-chlorobenzene (1n)**

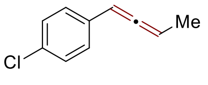


$^1H$ NMR (400 MHz, $CDCl_3$) δ 7.28–7.23 (m, 2H), 7.22–7.18 (m, 2H), 6.05 (dq, *J* = 6.4, 3.2 Hz, 1H), 5.55 (p, *J* = 7.0 Hz, 1H), 1.79 (dd, *J* = 7.1, 3.2 Hz, 3H). $^{13}C\{^1H\}$ NMR (101 MHz, $CDCl_3$) δ 206.1, 133.6, 132.2, 128.7, 127.8, 93.1, 90.0, 14.0. GC-MS (EI): calculated for $C_{10}H_9Cl$: 164.04; found: 164.05. The NMR data of this compound was in accordance with reported literature.[6]

**1-Bromo-4-(buta-1,2-dien-1-yl)benzene (1o)**

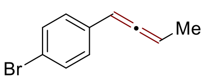


$^1$H NMR (400 MHz, $CDCl_3$) δ 7.43–7.38 (m, 2H), 7.16–7.12 (m, 2H), 6.03 (dq, *J* = 6.4, 3.2 Hz, 1H), 5.53 (p, *J* = 7.0 Hz, 1H), 1.78 (dd, *J* = 7.1, 3.2 Hz, 3H). $^{13}C\{^1H\}$ NMR (101 MHz, $CDCl_3$) δ 206.1, 134.1, 131.6, 128.2, 120.2, 93.2, 90.1, 13.9. GC-MS (EI): calculated for $C_{10}H_9Br$: 207.99; found: 208.00. The NMR data of this compound was in accordance with reported literature.[6]

**1-(Buta-1,2-dien-1-yl)-4-iodobenzene (1p)**

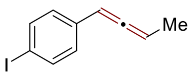


$^1$H NMR (400 MHz, $CDCl_3$) δ 7.62–7.58 (m, 2H), 7.04–6.99 (m, 2H), 6.01 (dq, *J* = 6.4, 3.2 Hz, 1H), 5.54 (p, *J* = 7.1 Hz, 1H), 1.78 (dd, *J* = 7.1, 3.2 Hz, 3H). $^{13}C\{^1H\}$ NMR (101 MHz, $CDCl_3$) δ 206.2, 137.6, 134.8, 128.5, 93.3, 91.5, 90.1, 13.9. GC-MS (EI): calculated for $C_{10}H_9I$: 255.97; found: 255.95.

**(4-(Buta-1,2-dien-1-yl)phenyl)trimethylsilane (1q)**

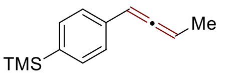


$^1$H NMR (400 MHz, $CDCl_3$) δ 7.46 (d, *J* = 8.1 Hz, 2H), 7.28 (d, *J* = 8.0 Hz, 2H), 6.09 (dq, *J* = 6.4, 3.2 Hz, 1H), 5.54 (p, *J* = 7.0 Hz, 1H), 1.79 (dd, *J* = 7.1, 3.2 Hz, 3H), 0.26 (d, *J* = 0.6 Hz, 9H). $^{13}C\{^1H\}$ NMR (101 MHz, $CDCl_3$) δ 206.2, 138.7, 135.6, 133.6, 126.0, 94.0, 89.5, 14.1, -1.1. GC-MS (EI): calculated for $C_{13}H_{18}Si$: 202.12, found: 202.10. The NMR data of this compound was in accordance with reported literature.[6]

**4-(Buta-1,2-dien-1-yl)benzonitrile (1r)**

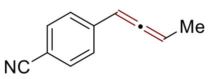


$^1$H NMR (400 MHz, $CDCl_3$) δ 7.59–7.54 (m, 2H), 7.37–7.33 (m, 2H), 6.10 (dq, *J* = 6.3, 3.2 Hz, 1H), 5.63 (p, *J* = 7.1 Hz, 1H), 1.81 (dd, *J* = 7.2, 3.2 Hz, 3H). $^{13}C\{^1H\}$ NMR (101 MHz, $CDCl_3$) δ 207.4, 140.4, 132.3, 127.1, 119.1, 109.8, 93.4, 90.6, 13.7. GC-MS (EI): calculated for $C_{11}H_9N$: 155.07; found: 155.10. The NMR data of this compound was in accordance with reported literature.[12]

**2-(4-(Buta-1,2-dien-1-yl)phenyl)-4,4,5,5-tetramethyl-1,3,2-dioxaborolane (1s)**

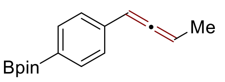


$^1$H NMR (400 MHz, $CDCl_3$) δ 7.75 (d, *J* = 8.0 Hz, 2H), 7.30 (d, *J* = 8.0 Hz, 2H), 6.12 (dq, *J* = 6.4, 3.2 Hz, 1H), 5.57 (p, *J* = 7.0 Hz, 1H), 1.81 (dd, *J* = 7.1, 3.2 Hz, 3H), 1.36 (s, 13H). $^{13}C\{^1H\}$ NMR (101 MHz, $CDCl_3$) δ 206.6, 138.1, 135.0, 126.0, 94.2, 89.6, 83.7, 24.9, 24.9, 14.0. GC-MS (EI): calculated for $C_{16}H_{21}BO_2$: 256.16; found: 256.20.

**(4-(Buta-1,2-dien-1-yl)phenyl)(methyl)sulfane (1t)**

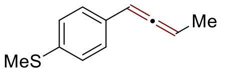


$^1$H NMR (400 MHz, $CDCl_3$) δ 7.20 (s, 4H), 6.05 (dq, *J* = 6.4, 3.2 Hz, 1H), 5.53 (p, *J* = 7.0 Hz, 1H), 2.47 (s, 3H), 1.78 (dd, *J* = 7.1, 3.2 Hz, 3H). $^{13}C\{^1H\}$ NMR (101 MHz, $CDCl_3$) δ 205.9, 136.5, 132.1, 127.1, 127.0, 93.5, 89.8, 16.2, 14.1. GC-MS (EI): calculated for $C_{11}H_{12}S$: 176.07; found: 176.10.

**Methyl 4-(buta-1,2-dien-1-yl)benzoate (1u)**

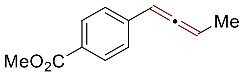


$^1$H NMR (400 MHz, $CDCl_3$) δ 8.00–7.91 (m, 2H), 7.37–7.29 (m, 2H), 6.12 (dq, *J* = 6.4, 3.2 Hz, 1H), 5.59 (p, *J* = 7.0 Hz, 1H), 3.90 (s, 3H), 1.80 (dd, *J* = 7.2, 3.2 Hz, 3H). $^{13}C\{^1H\}$ NMR (101 MHz, $CDCl_3$) δ 207.1,

167.0, 140.2, 129.9, 128.2, 126.5, 93.7, 90.1, 52.0, 13.8. GC-MS (EI): calculated for $C_{12}H_{12}O_2$: 188.08; found: 188.05. The NMR data of this compound was in accordance with reported literature.[12]

**3-(Buta-1,2-dien-1-yl)thiophene (1v)**

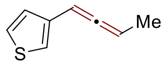

$^1$H NMR (400 MHz, $CDCl_3$) δ 7.27 – 7.24 (m, 1H), 7.07 (dd, *J* = 5.0, 1.2 Hz, 1H), 7.05 (d, *J* = 3.0 Hz, 1H), 6.17 (dq, *J* = 6.4, 3.2 Hz, 1H), 5.51 – 5.42 (m, 1H), 1.77 (dd, *J* = 7.1, 3.2 Hz, 3H). $^{13}$C{$^1$H} NMR (101 MHz, $CDCl_3$) δ 206.2, 136.4, 126.3, 125.7, 120.2, 88.8, 88.6, 14.2. GC-MS (EI): calculated for $C_8H_8S$: 136.03, found: 136.10. The NMR data of this compound was in accordance with reported literature.[6]

**2-(Buta-1,2-dien-1-yl)-5-(methoxymethyl)furan (1w)**

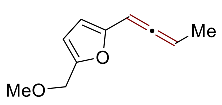

$^1$H NMR (400 MHz, $CDCl_3$) δ 6.22 (d, *J* = 3.2 Hz, 1H), 6.06 (d, *J* = 3.2 Hz, 1H), 5.96 (dq, *J* = 6.6, 3.3 Hz, 1H), 5.52–5.44 (m, 1H), 4.29 (s, 2H), 3.28 (s, 3H), 1.69 (dd, *J* = 7.2, 3.3 Hz, 3H). $^{13}$C{$^1$H} NMR (101 MHz, $CDCl_3$) δ 205.2, 150.8, 149.3, 111.2, 107.3, 90.2, 84.7, 66.3, 57.7, 14.2. GC-MS (EI): calculated for $C_{10}H_{12}O_2$: 164.08, found: 164.10.

**Deca-2,3-diene (1x)**

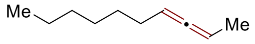

$^1$H NMR (500 MHz, $CDCl_3$) δ 5.07–5.00 (m, 2H), 2.01–1.93 (m, 2H), 1.67–1.61 (m, 3H), 1.42-1.36 (m, 2H), 1.35–1.22 (m, 6H), 0.89 (t, J = 6.8 Hz, 3H). $^{13}$C{$^1$H} NMR (126 MHz, $CDCl_3$) δ 204.7, 90.4, 85.3, 31.7, 29.1, 28.9, 28.7, 22.7, 14.6, 14.1. GC-MS (EI): calculated for $C_{10}H_{18}$: 138.14; found: 138.20. The NMR data of this compound was in accordance with reported literature.[13]

**Buta-1,2-dien-1-ylcyclohexane (1y)**

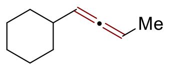

$^1$H NMR (500 MHz, $CDCl_3$) δ 5.10–5.06 (m, 1H), 5.05–5.01 (m, 1H), 1.98–1.91 (m, 1H), 1.77–1.69 (m, 4H), 1.66–1.64 (m, 3H), 1.32–1.23 (m, 3H), 1.21–1.15 (m, 1H), 1.12–1.04 (m, 2H). $^{13}$C{$^1$H}NMR (126 MHz, $CDCl_3$) δ 203.5, 96.5, 86.2, 37.2, 33.1, 33.1, 26.2, 26.0, 26.0, 14.8. GC-MS (EI): calculated for $C_{10}H_{16}$: 136.13; found: 136.20. The NMR data of this compound was in accordance with reported literature.[14]

## X. References:

(1) K. Endo, M. Hirokami, T. Shibata, T. *J. Org. Chem.* 2010, **75**, 3469-3472.

(2) M. Xiong, X. Xie, Y. Liu, *Org. Lett.* **2017**, *19*, 3398-3401.

(3) A. Noble, S. Roesner, V. K. Aggarwal, *Angew. Chem. Int. Ed.* **2016**, *55*, 15920-15924.

(4) E. E. Schultz, R. Sarpong, *J. Am. Chem. Soc*. **2013**, *135*, 4696-4699.

(5) Huang, C.-W.; Shanmugasundaram, M.; Chang, H.-M.; Cheng, C.-H., *Tetrahedron* **2003**, *59*, 3635-3641.

(6) Khrakovsky, D. A.; Tao, C.; Johnson, M. W.; Thornbury, R. T.; Shevick, S. L.; Toste, F. D., *Angew. Chem. Int. Ed.* **2016**, *55*, 6079-6083.

(7) Jiang, G.-J.; Zheng, Q.-H.; Dou, M.; Zhuo, L.-G.; Meng, W.; Yu, Z.-X., *J. Org. Chem.* **2013**, *78*, 11783-11793

(8) Lustosa, D. M.; Clemens, S.; Rudolph, M.; Hashmi, A. S. K., *Adv. Synth. Catal.* **2019**, *361*, 5050-5056.

(9) Bolte, B.; Odabachian, Y.; Gagosz, F., *J. Am. Chem. Soc.* **2010**, *132*, 7294-7296.

(10) Zhang, H.; Fu, X.; Chen, J.; Wang, E.; Liu, Y.; Li, Y., *J. Org. Chem.* **2009**, *74*, 9351-9358.

(11) Kidonakis, M.; Stratakis, M., *Org. L*ett. **2015**, *17*, 4538-4541.

(12) Harris, R. J.; Nakafuku, K.; Widenhoefer, R. A., *Chem. - Eur. J.* **2014**, *20*, 12245-12254.

(13) Konoike, T.; Araki, Y., *Tetrahedron Lett.* **1992**, *33*, 5093-5096.

(14) Ng, S.-S.; Jamison, T. F., *Tetrahedron* **2005**, *61*, 11405-11417.

# XI. Copies of ¹H NMR and $^{13}C\{^1H\}$ NMR Spectra

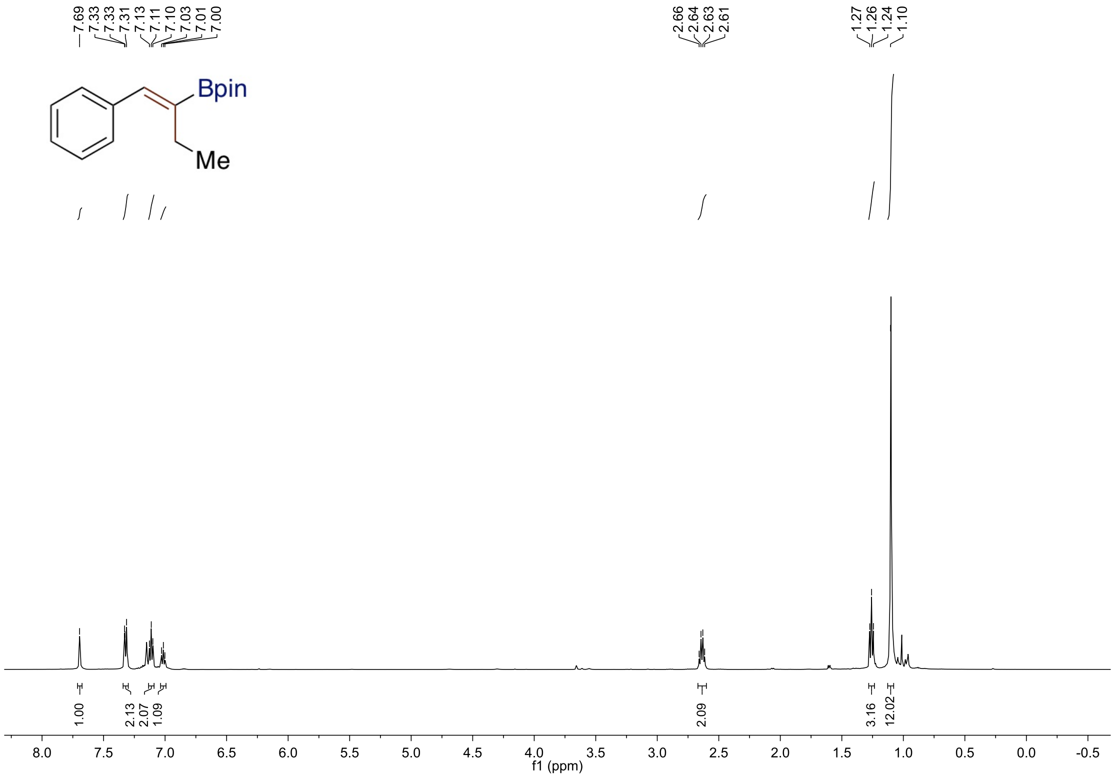


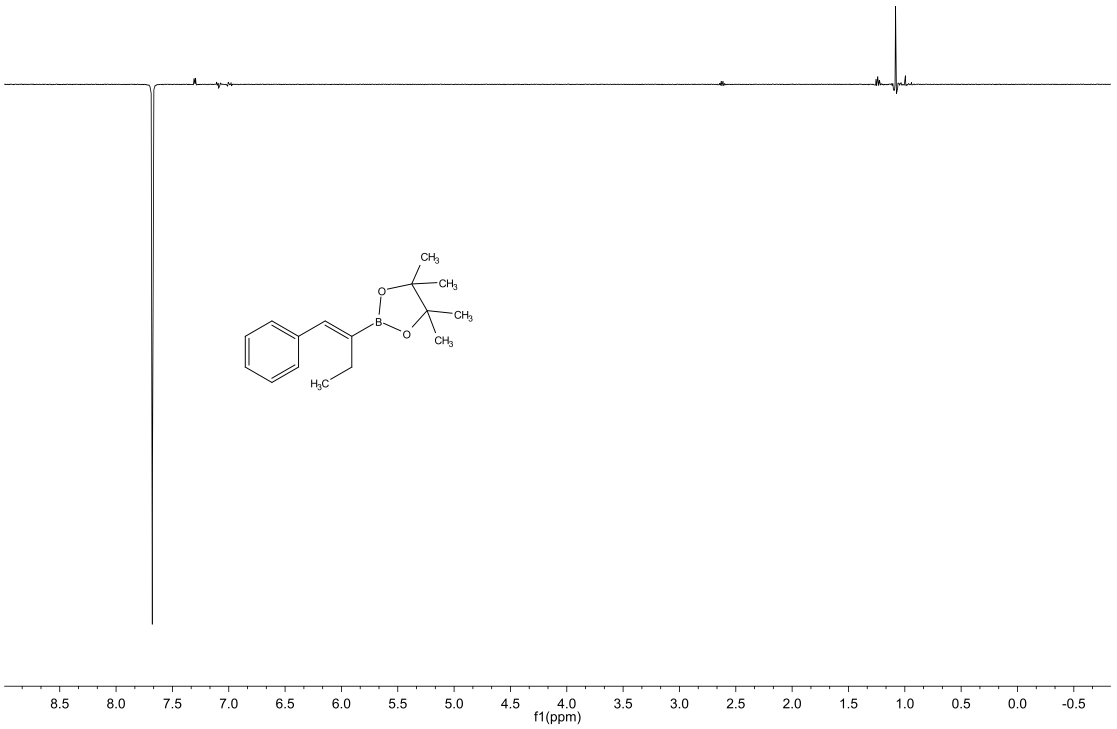

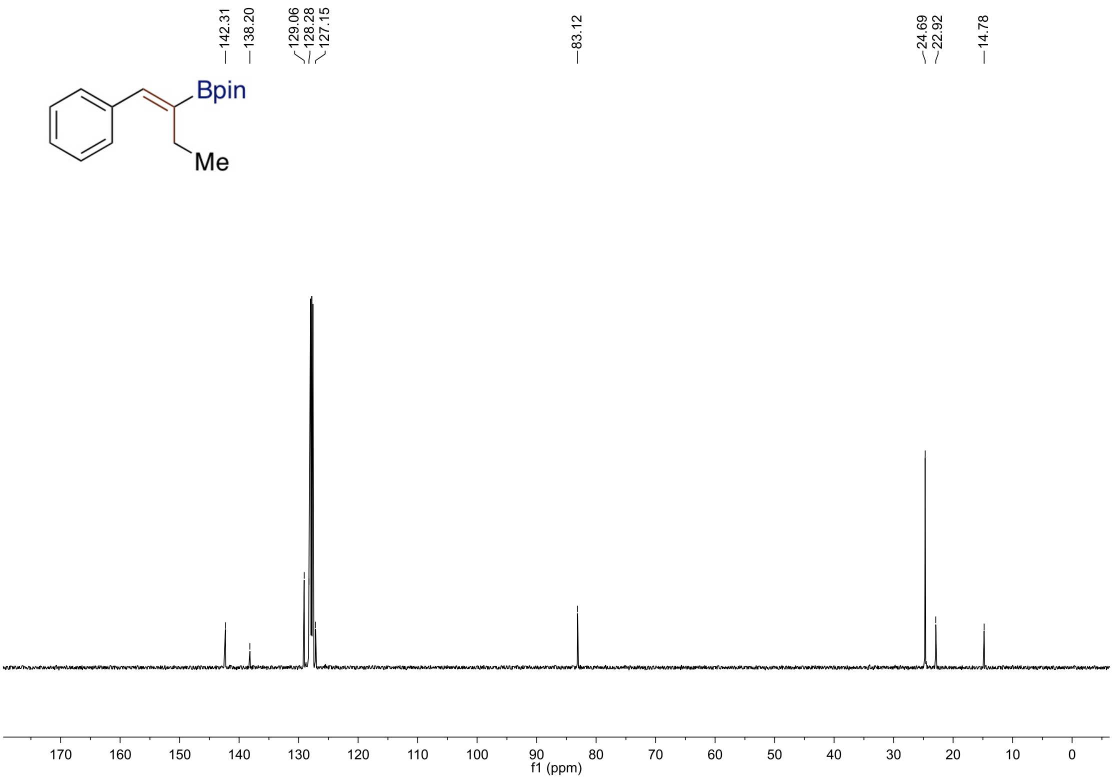
142.31
138.20
129.06
128.28
127.15
83.12
24.69
22.92
14.78
Bpin
Me
170
160
150
140
130
120
110
100
90
80
70
60
50
40
30
20
10
0
f1 (ppm)

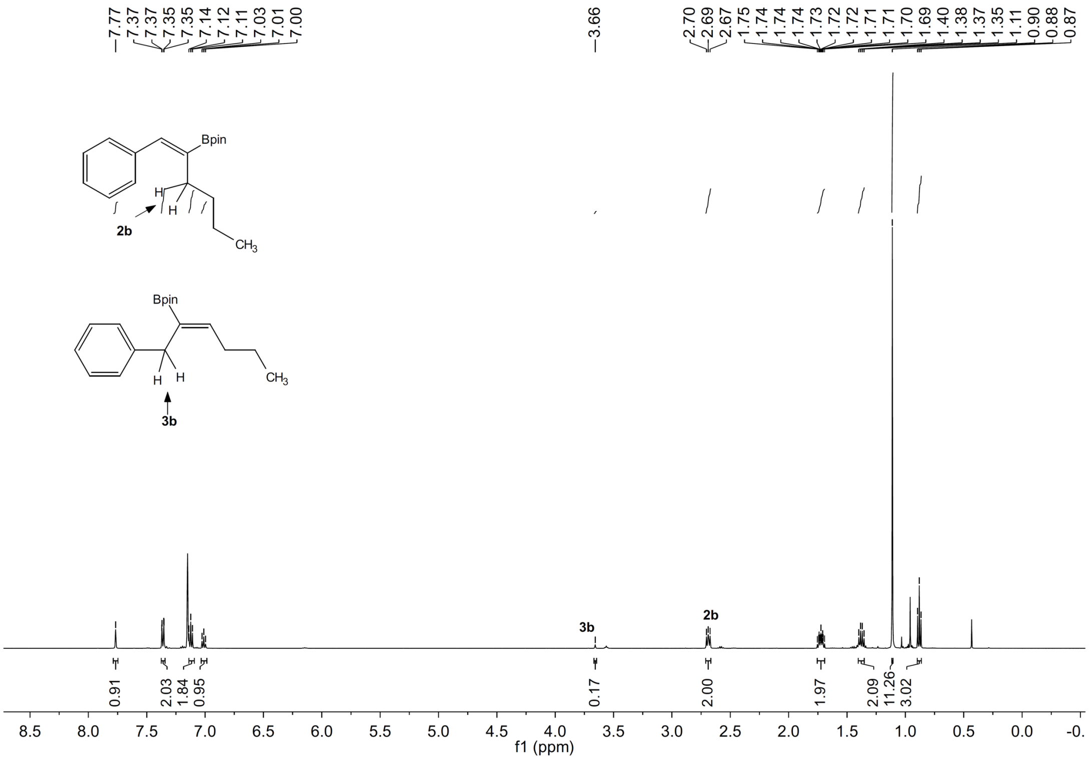

7.77 7.37 7.37 7.35 7.35 7.14 7.12 7.11 7.03 7.01 7.00
3.66
2.70 2.69 2.67 1.75 1.74 1.74 1.74 1.73 1.72 1.72 1.71 1.71 1.70 1.69 1.40 1.38 1.37 1.35 1.11 0.90 0.88 0.87
Bpin
H
H
2b
CH3
Bpin
H H
CH3
3b
3b
2b
0.91 2.03 1.84 0.95 0.17 2.00 1.97 2.09 11.26 3.02
8.5 8.0 7.5 7.0 6.5 6.0 5.5 5.0 4.5 4.0 3.5 3.0 2.5 2.0 1.5 1.0 0.5 0.0 -0.
f1 (ppm)


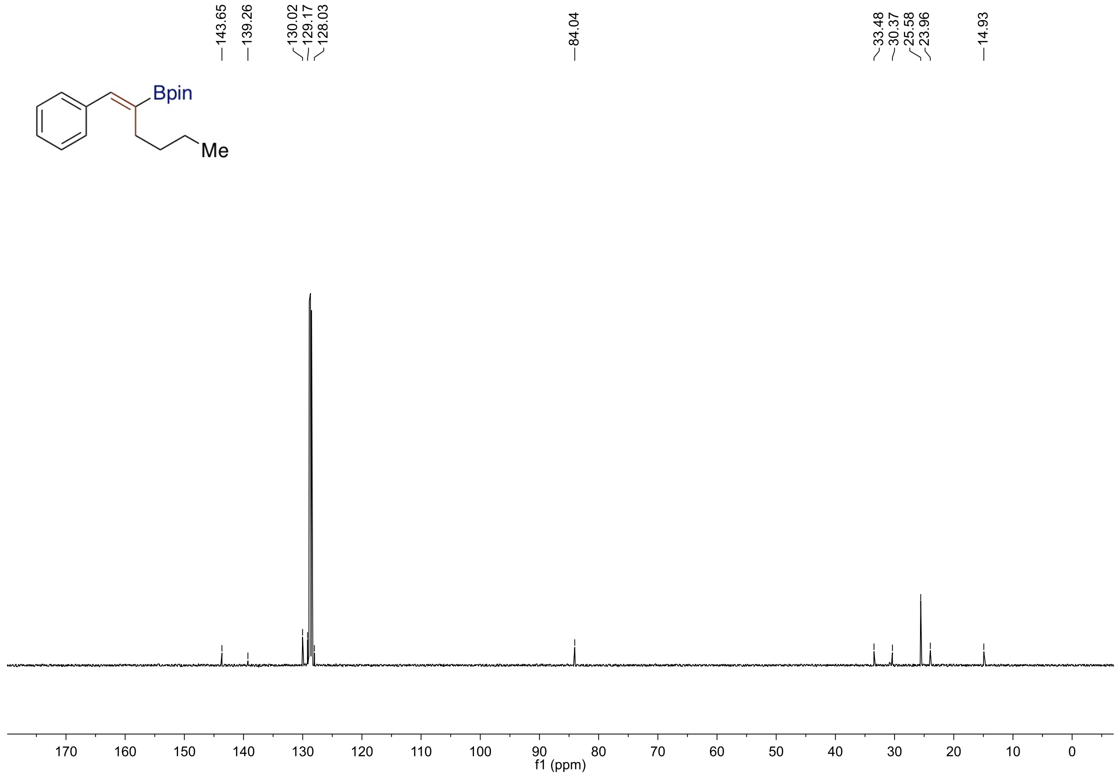

143.65 139.26 130.02 129.17 128.03
84.04
33.48 30.37 25.58 23.96 14.93
Bpin
Me
170 160 150 140 130 120 110 100 90 80 70 60 50 40 30 20 10 0
f1 (ppm)

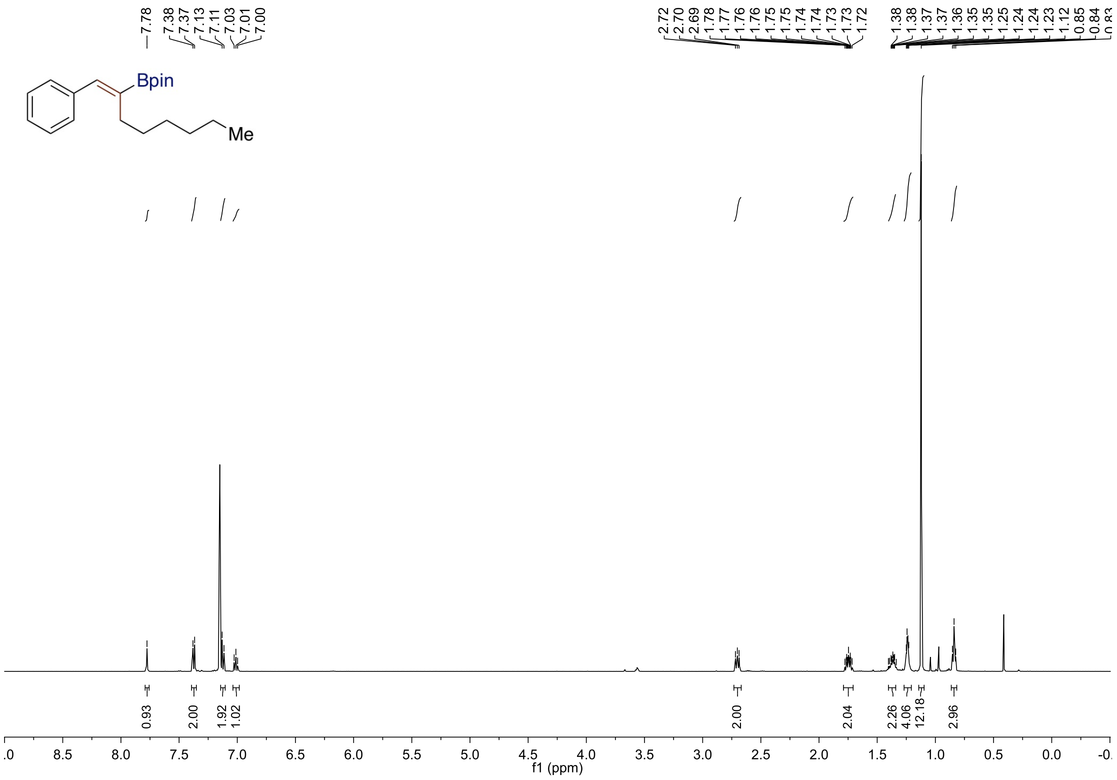

7.78
7.38 7.37 7.13 7.11 7.03 7.01 7.00
2.72 2.70 2.69 1.78 1.77 1.76 1.76 1.75 1.75 1.74 1.74 1.73 1.73 1.72
1.38 1.38 1.37 1.37 1.36 1.35 1.25 1.24 1.24 1.23 1.12 0.85 0.84
Bpin
Me
0.93 2.00 1.92 1.02 2.00 2.04 2.26 4.06 12.18 2.96
f1 (ppm)


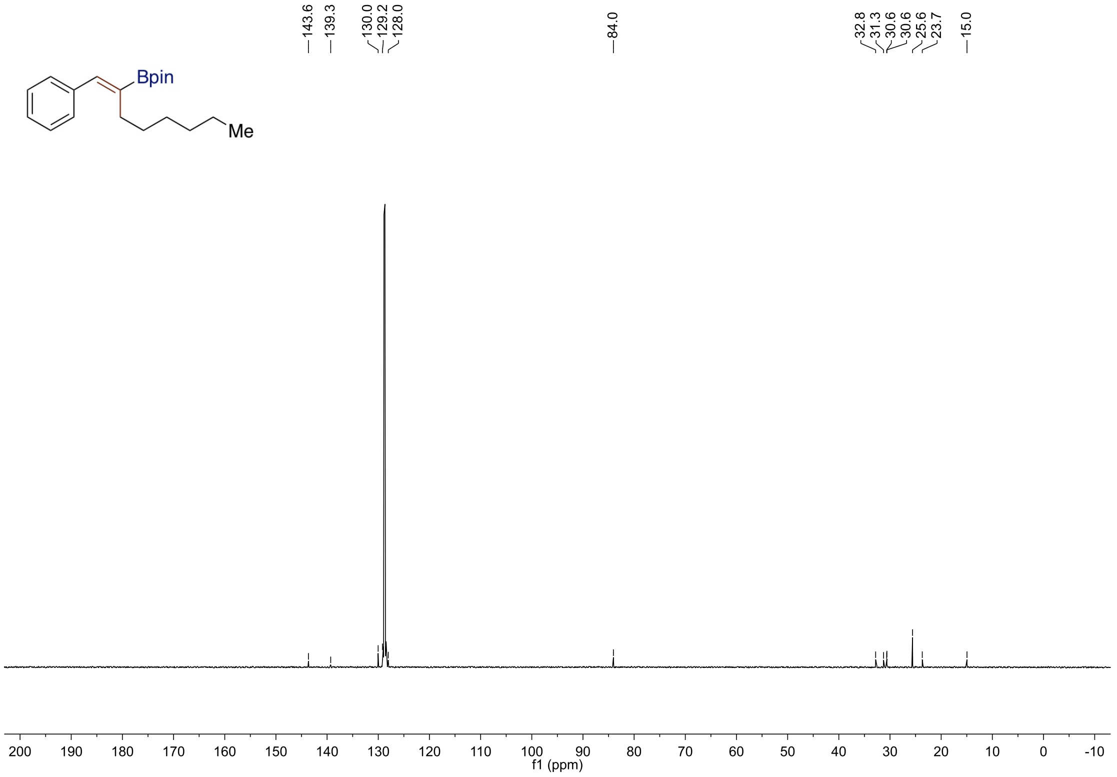

143.6 139.3 130.0 129.2 128.0
84.0
32.8 31.3 30.6 30.6 25.6 23.7 15.0
Bpin
Me
f1 (ppm)

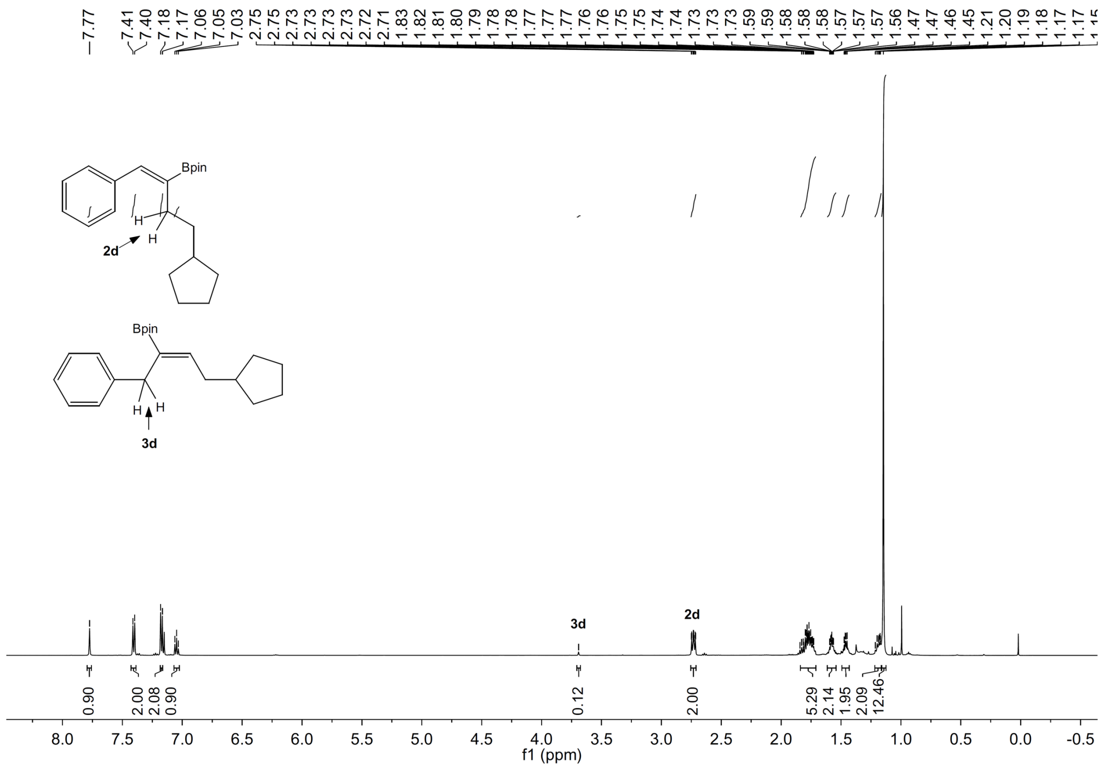
7.77
7.41
7.40
7.18
7.17
7.06
7.05
7.03
2.75
2.75
2.73
2.73
2.73
2.73
2.72
2.71
1.83
1.82
1.81
1.80
1.79
1.78
1.78
1.77
1.77
1.77
1.76
1.75
1.75
1.74
1.74
1.73
1.73
1.73
1.59
1.58
1.58
1.58
1.57
1.57
1.57
1.56
1.47
1.47
1.46
1.45
1.21
1.20
1.19
1.18
1.17
1.17
Bpin
H
H
2d
Bpin
H
H
3d
3d
2d
0.90
2.00
2.08
0.90
0.12
2.00
5.29
2.14
1.95
2.09
12.46
8.0
7.5
7.0
6.5
6.0
5.5
5.0
4.5
4.0
3.5
3.0
2.5
2.0
1.5
1.0
0.5
0.0
-0.5
f1 (ppm)

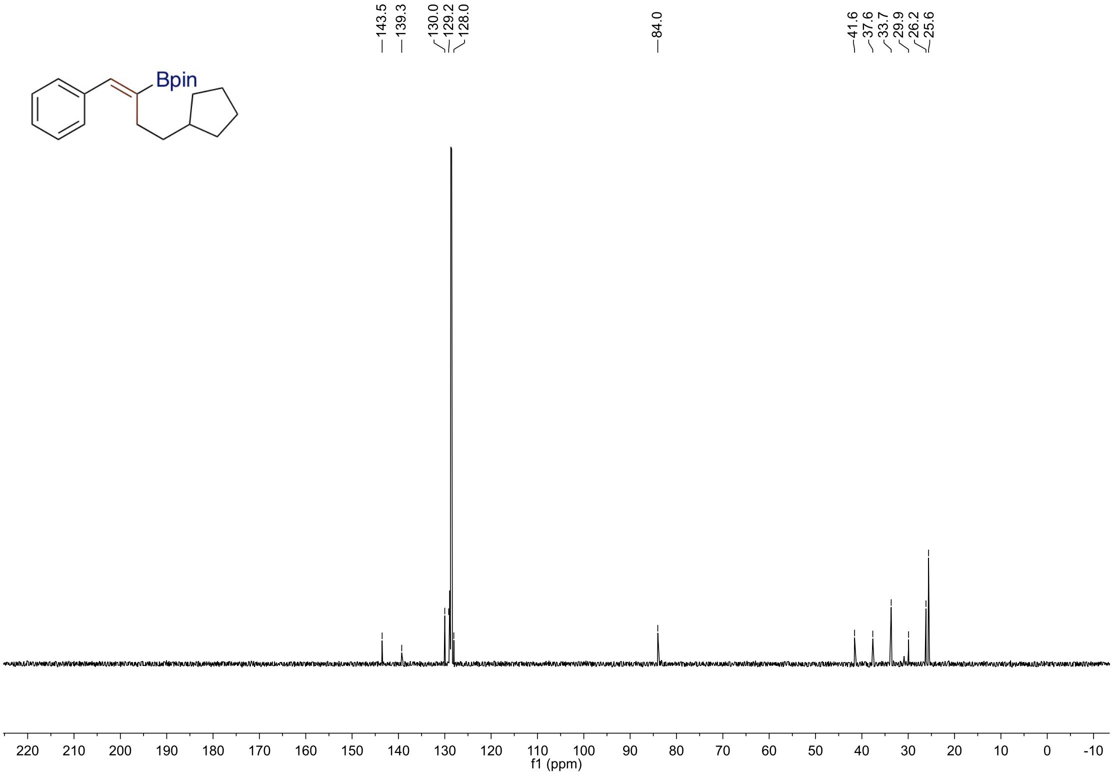
143.5
139.3
130.0
129.2
128.0
84.0
41.6
37.6
33.7
29.9
26.2
25.6
Bpin
220
210
200
190
180
170
160
150
140
130
120
110
100
90
80
70
60
50
40
30
20
10
0
-10
f1 (ppm)

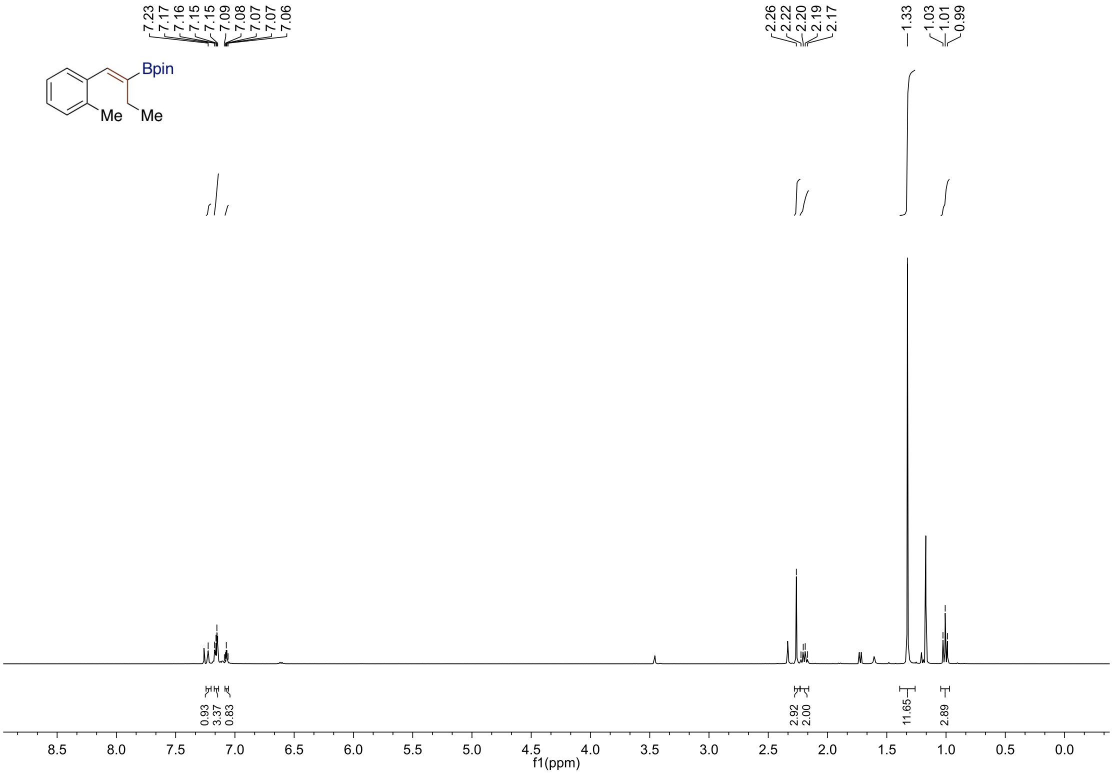
7.23
7.17
7.16
7.15
7.15
7.09
7.08
7.07
7.07
7.06
2.26
2.22
2.20
2.19
2.17
1.33
1.03
1.01
0.99
Bpin
Me
Me
0.93
3.37
0.83
2.92
2.00
11.65
2.89
8.5
8.0
7.5
7.0
6.5
6.0
5.5
5.0
4.5
4.0
3.5
3.0
2.5
2.0
1.5
1.0
0.5
0.0
f1(ppm)

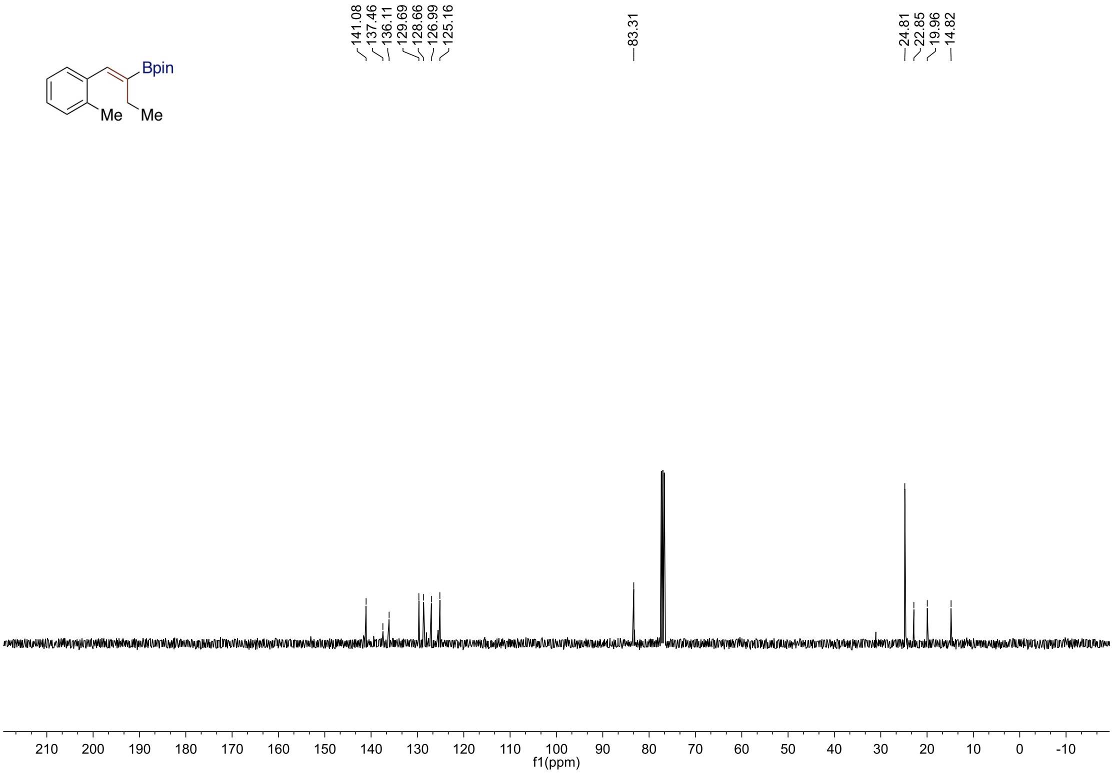
141.08
137.46
136.11
129.69
128.66
126.99
125.16
83.31
24.81
22.85
19.96
14.82
Bpin
Me
Me
210
200
190
180
170
160
150
140
130
120
110
100
90
80
70
60
50
40
30
20
10
0
-10
f1(ppm)

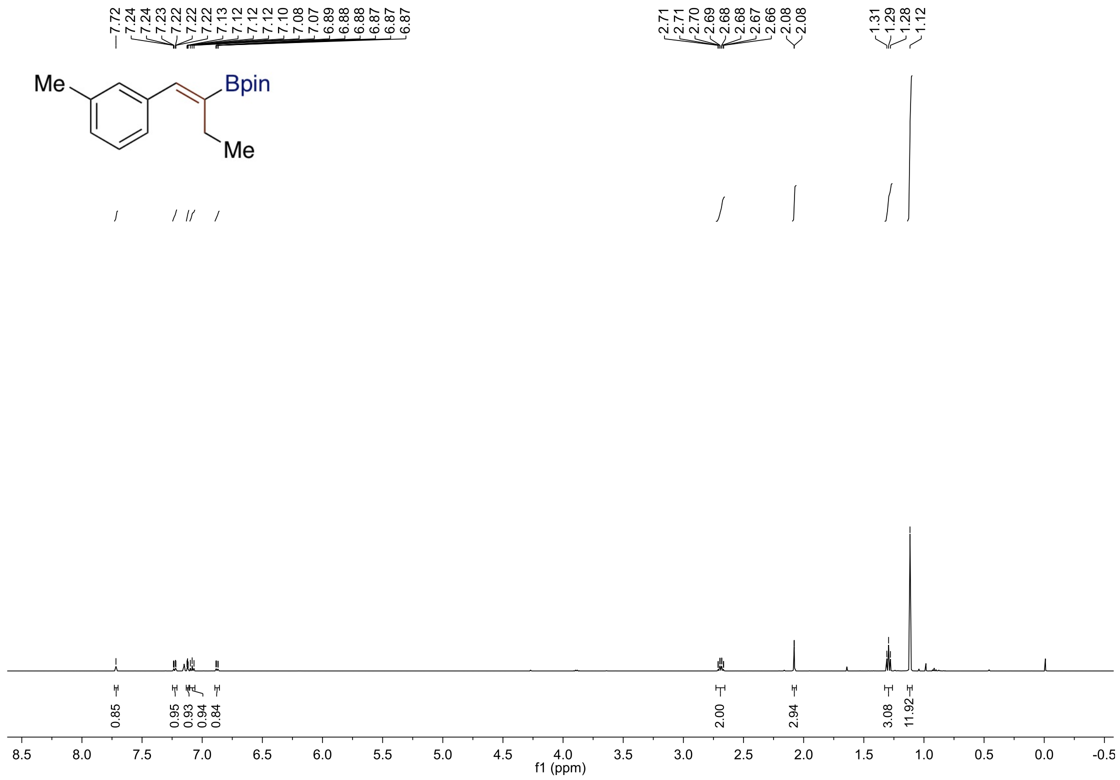

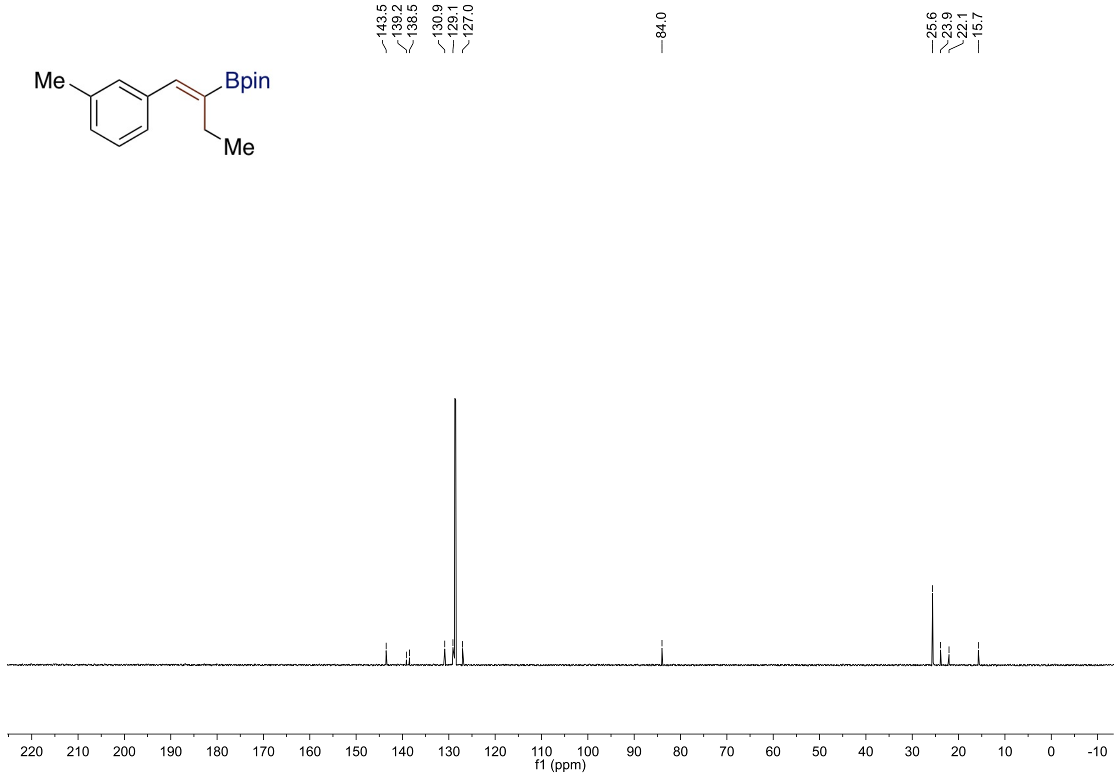

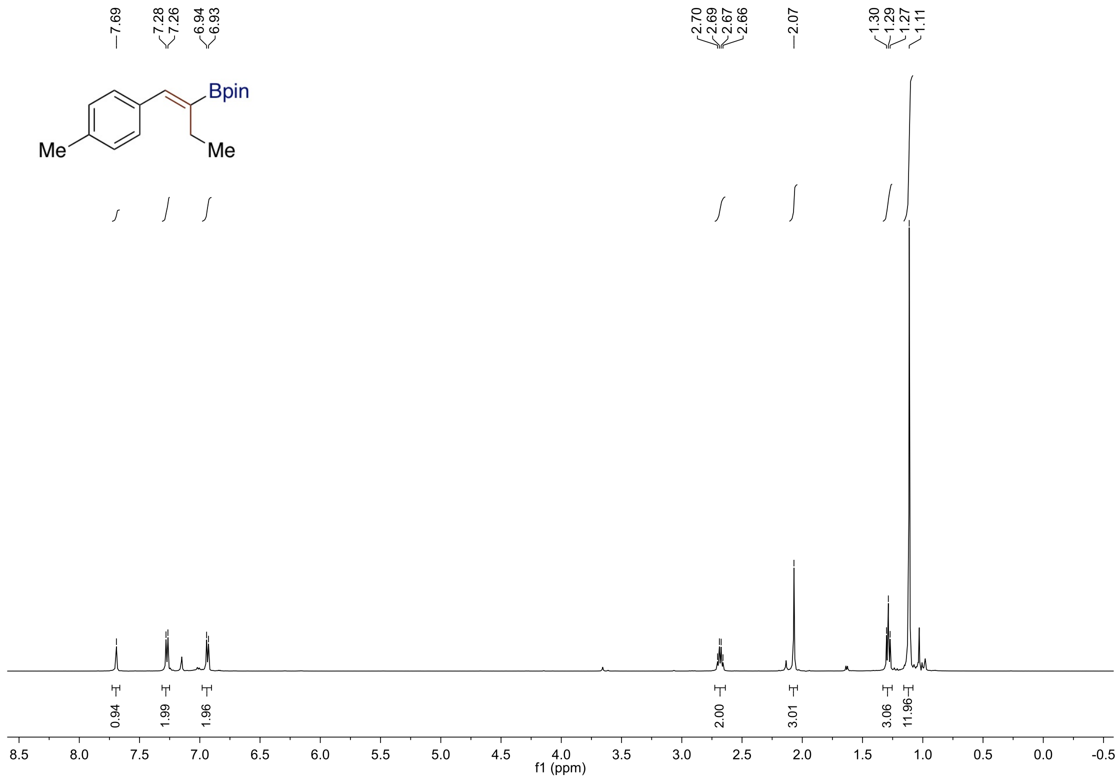
7.69
7.28
7.26
6.94
6.93
2.70
2.69
2.67
2.66
2.07
1.30
1.29
1.27
1.11
Bpin
Me
Me
0.94
1.99
1.96
2.00
3.01
3.06
11.96
f1 (ppm)

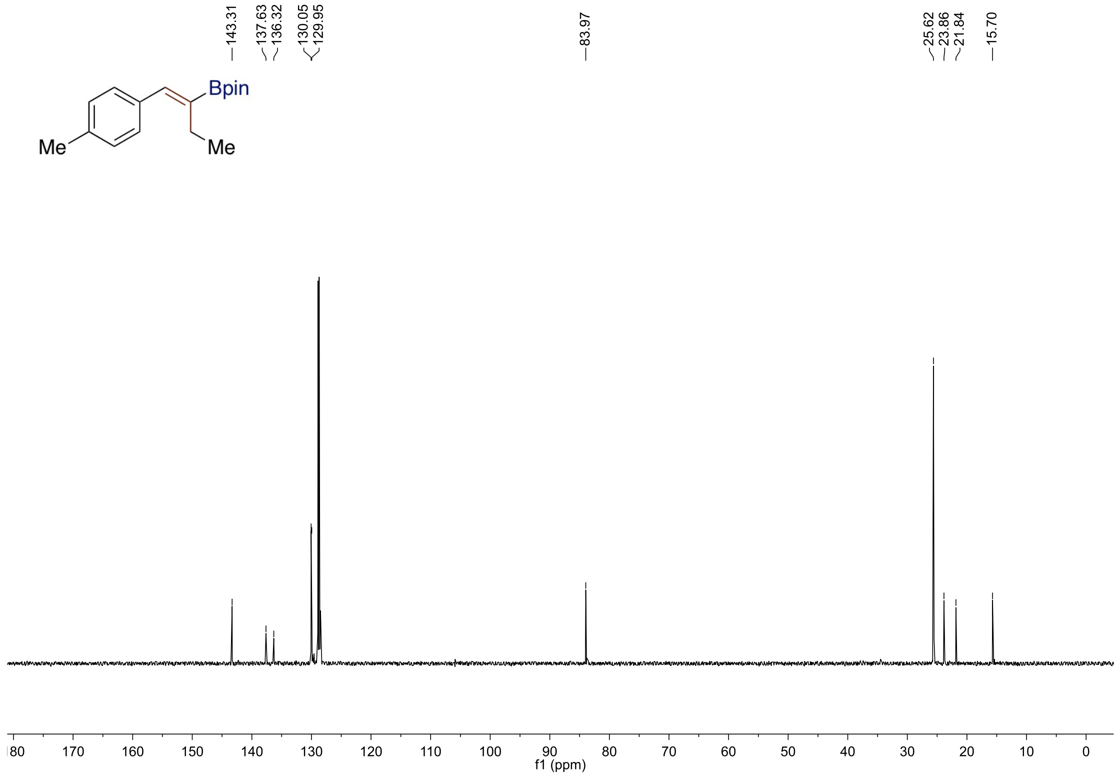
143.31
137.63
136.32
130.05
129.95
83.97
25.62
23.86
21.84
15.70
Bpin
Me
Me
f1 (ppm)

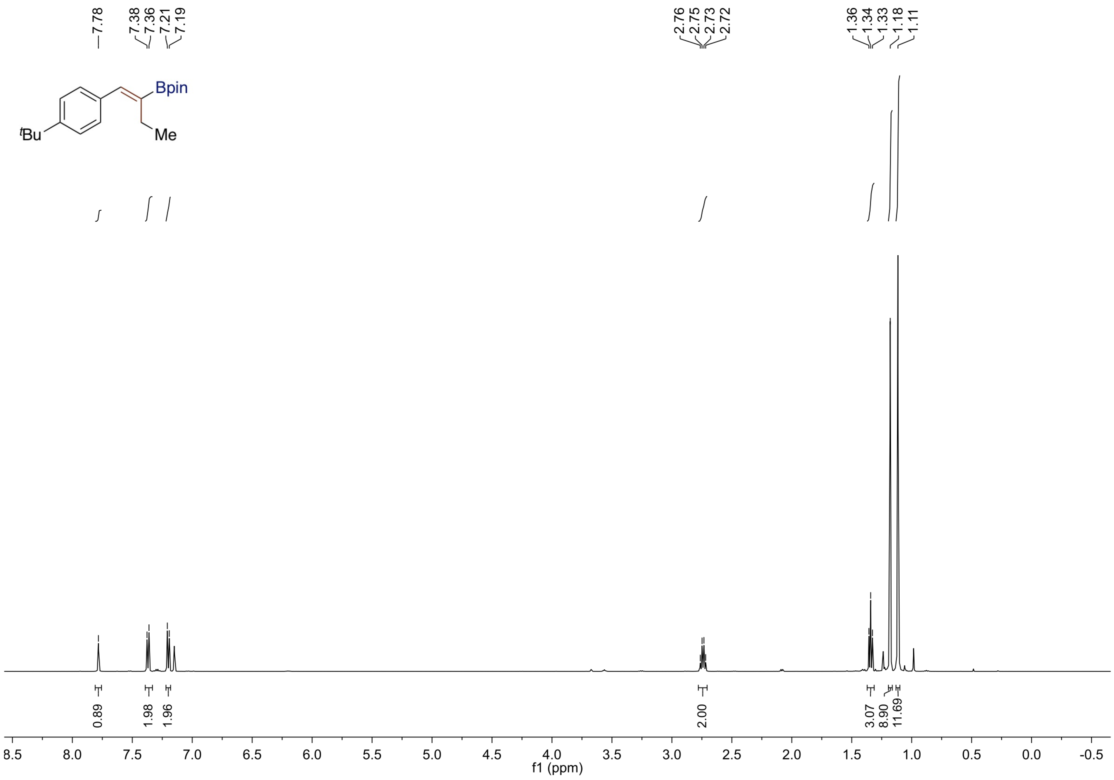
7.78
7.38
7.36
7.21
7.19
2.76
2.75
2.73
2.72
1.36
1.34
1.33
1.18
1.11
Bpin
ᵗBu
Me
0.89
1.98
1.96
2.00
3.07
8.90
11.69
8.5 8.0 7.5 7.0 6.5 6.0 5.5 5.0 4.5 4.0 3.5 3.0 2.5 2.0 1.5 1.0 0.5 0.0 -0.5
f1 (ppm)

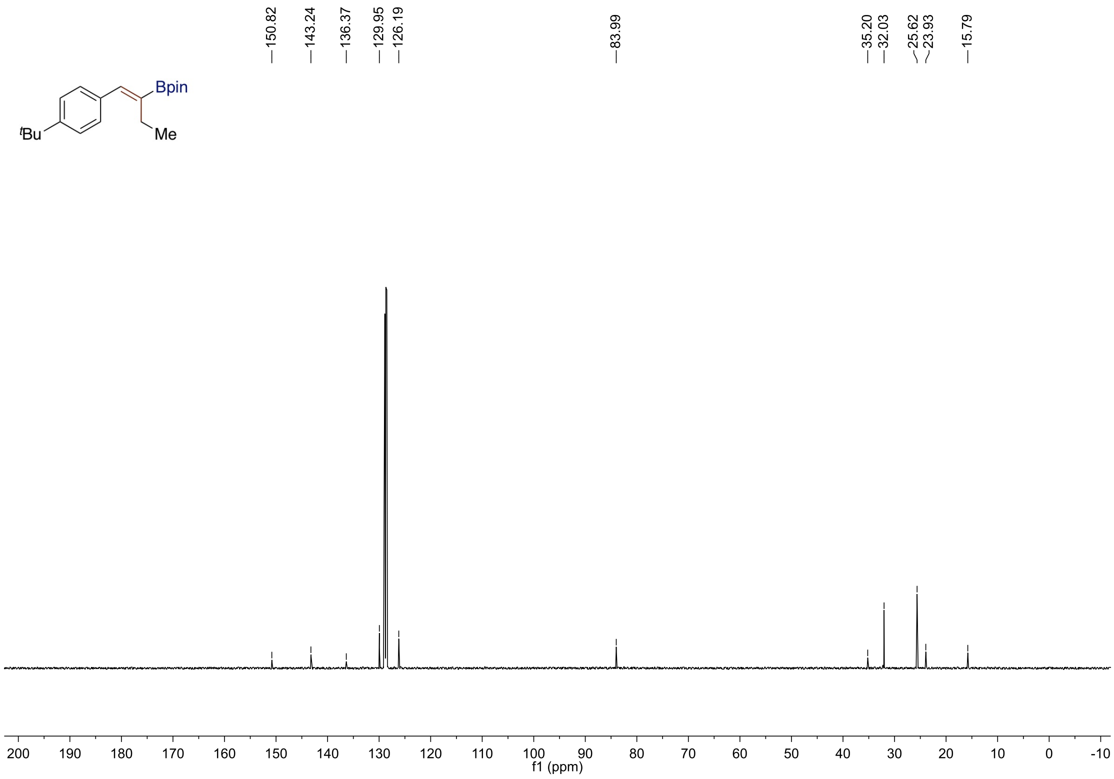
150.82
143.24
136.37
129.95
126.19
83.99
35.20
32.03
25.62
23.93
15.79
Bpin
ᵗBu
Me
200 190 180 170 160 150 140 130 120 110 100 90 80 70 60 50 40 30 20 10 0 -10
f1 (ppm)

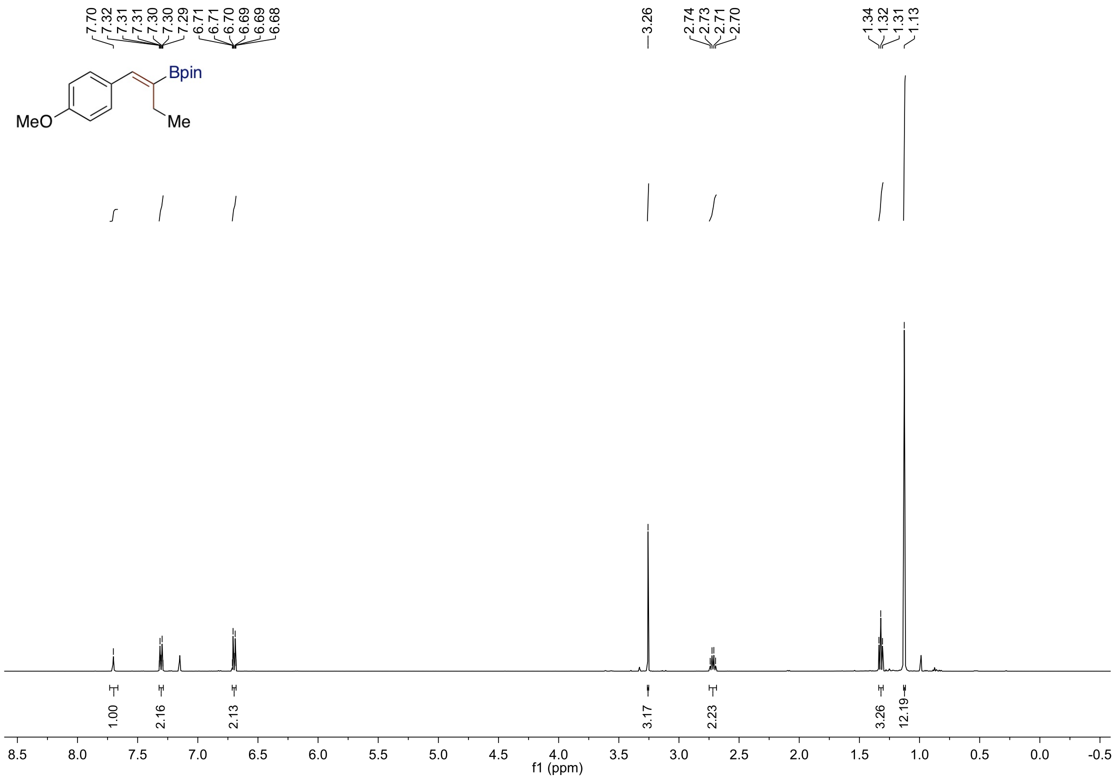
7.70
7.32
7.31
7.31
7.30
7.30
7.29
6.71
6.71
6.70
6.69
6.69
6.68
3.26
2.74
2.73
2.71
2.70
1.34
1.32
1.31
1.13
Bpin
MeO
Me
1.00
2.16
2.13
3.17
2.23
3.26
12.19
8.5 8.0 7.5 7.0 6.5 6.0 5.5 5.0 4.5 4.0 3.5 3.0 2.5 2.0 1.5 1.0 0.5 0.0 -0.5
f1 (ppm)

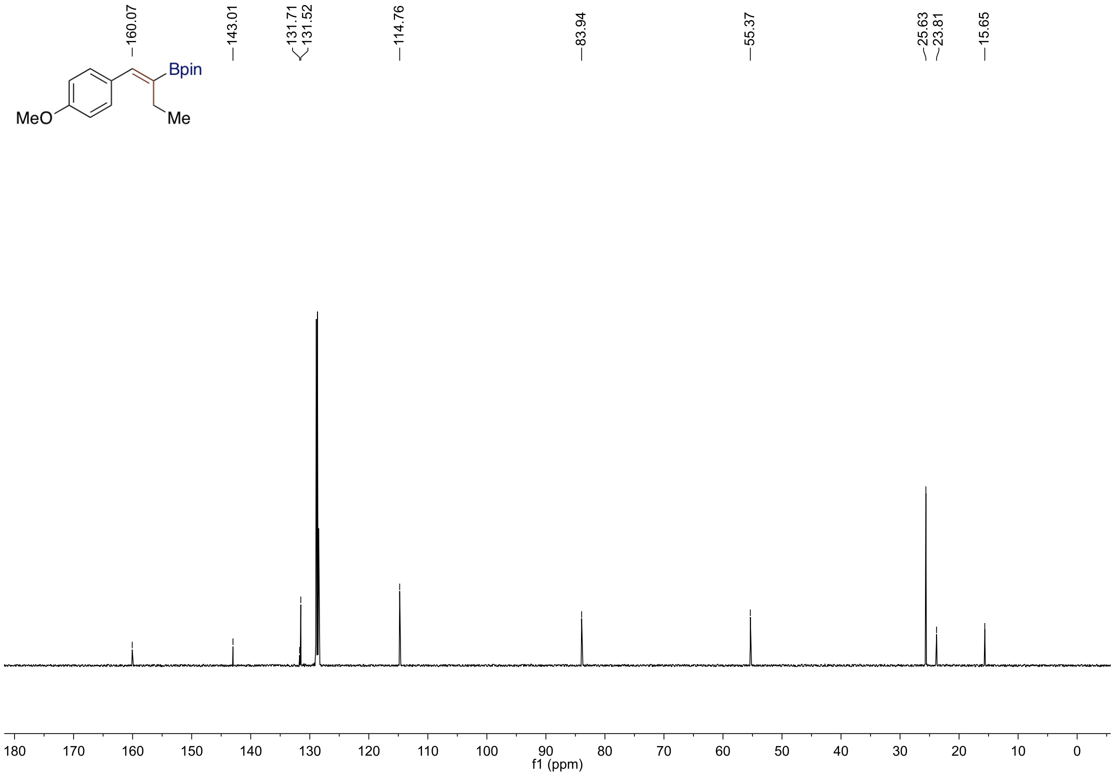
160.07
143.01
131.71
131.52
114.76
83.94
55.37
25.63
23.81
15.65
Bpin
MeO
Me
180 170 160 150 140 130 120 110 100 90 80 70 60 50 40 30 20 10 0
f1 (ppm)

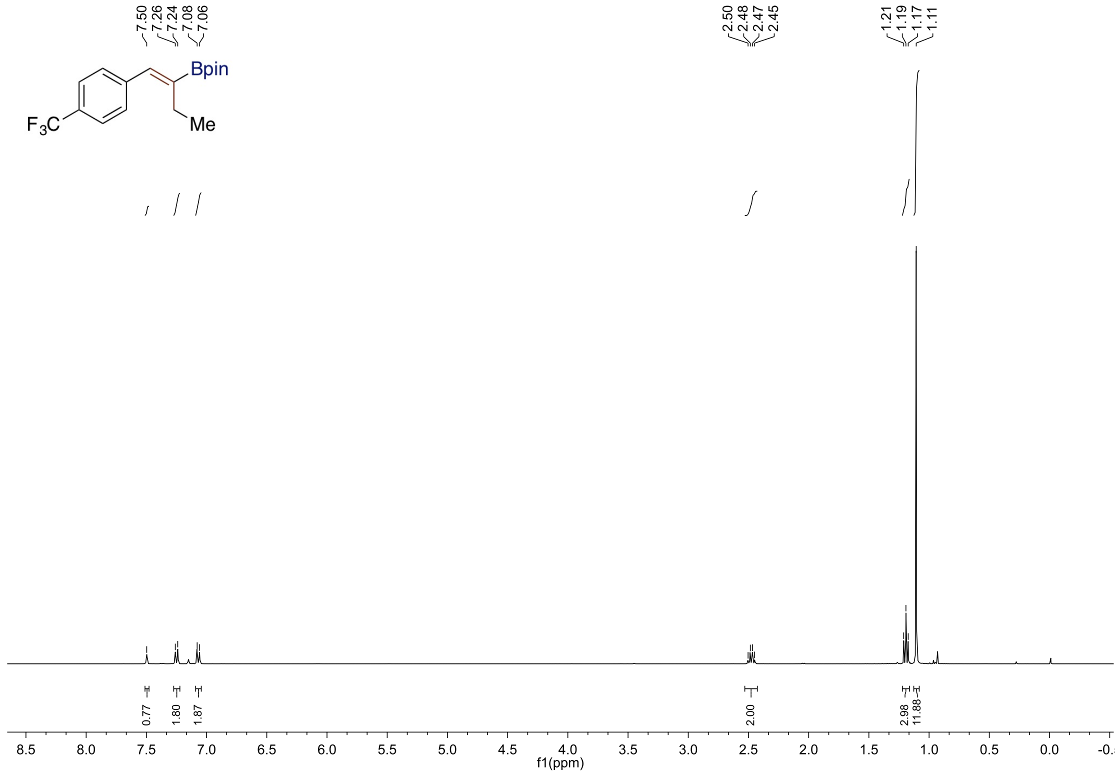
7.50
7.26
7.24
7.08
7.06
2.50
2.48
2.47
2.45
1.21
1.19
1.17
1.11
Bpin
F3C
Me
0.77
1.80
1.87
2.00
2.98
11.88
8.5 8.0 7.5 7.0 6.5 6.0 5.5 5.0 4.5 4.0 3.5 3.0 2.5 2.0 1.5 1.0 0.5 0.0
f1(ppm)

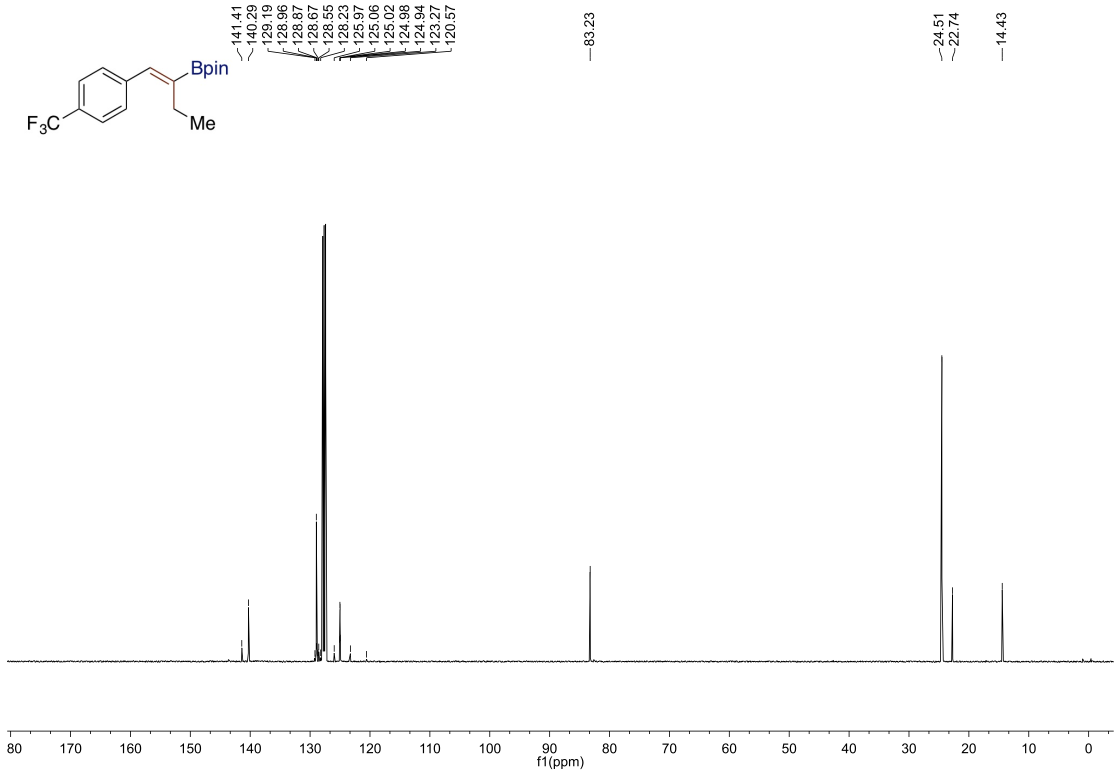
141.41
140.29
129.19
128.96
128.87
128.67
128.55
128.23
125.97
125.06
125.02
124.98
124.94
123.27
120.57
83.23
24.51
22.74
14.43
Bpin
F3C
Me
170 160 150 140 130 120 110 100 90 80 70 60 50 40 30 20 10 0
f1(ppm)

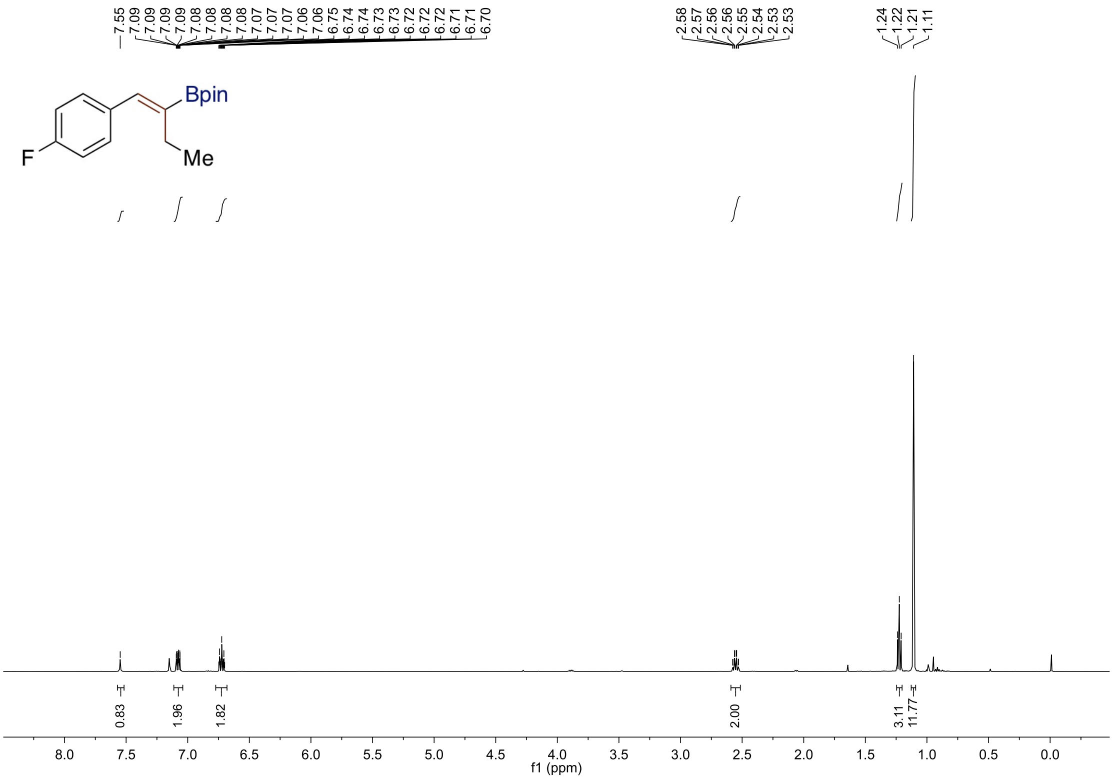

7.55
7.09
7.09
7.09
7.09
7.08
7.08
7.08
7.07
7.07
7.07
7.06
7.06
6.75
6.74
6.74
6.73
6.73
6.72
6.72
6.72
6.71
6.71
6.70
2.58
2.57
2.56
2.56
2.55
2.54
2.53
2.53
1.24
1.22
1.21
1.11
Bpin
F
Me
0.83
1.96
1.82
2.00
3.11
11.77
8.0
7.5
7.0
6.5
6.0
5.5
5.0
4.5
4.0
3.5
3.0
2.5
2.0
1.5
1.0
0.5
0.0
f1 (ppm)


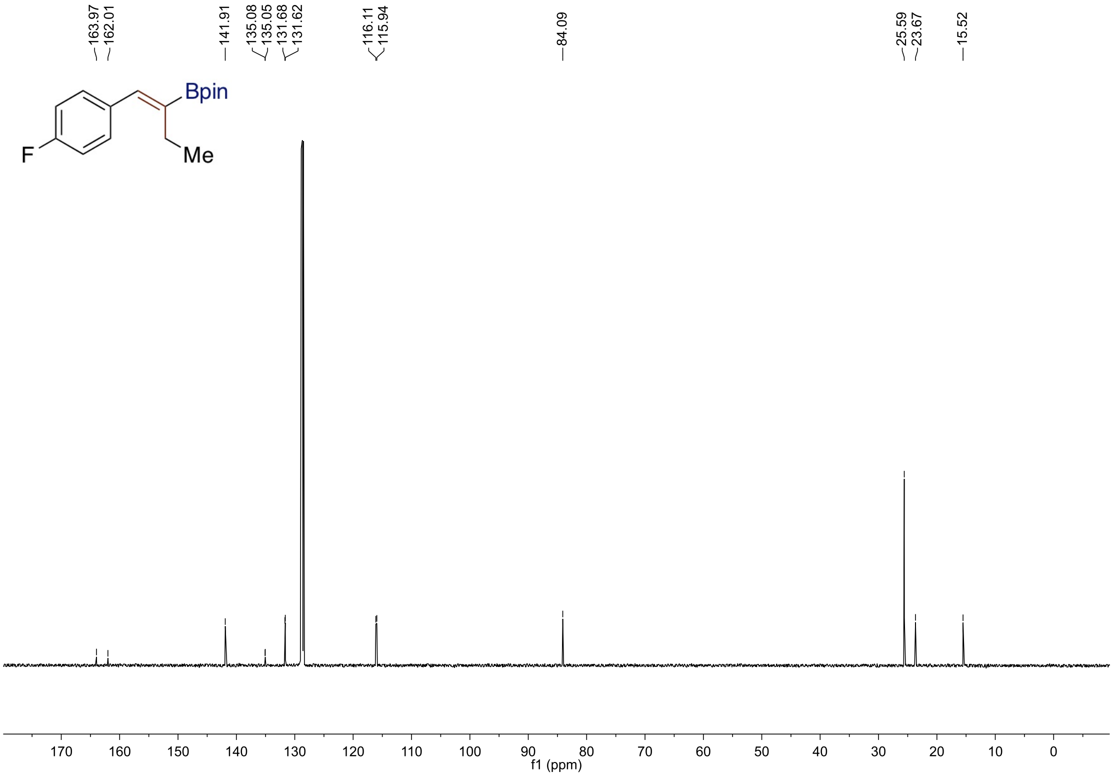

163.97
162.01
141.91
135.08
135.05
131.68
131.62
116.11
115.94
84.09
25.59
23.67
15.52
Bpin
F
Me
170
160
150
140
130
120
110
100
90
80
70
60
50
40
30
20
10
0
f1 (ppm)

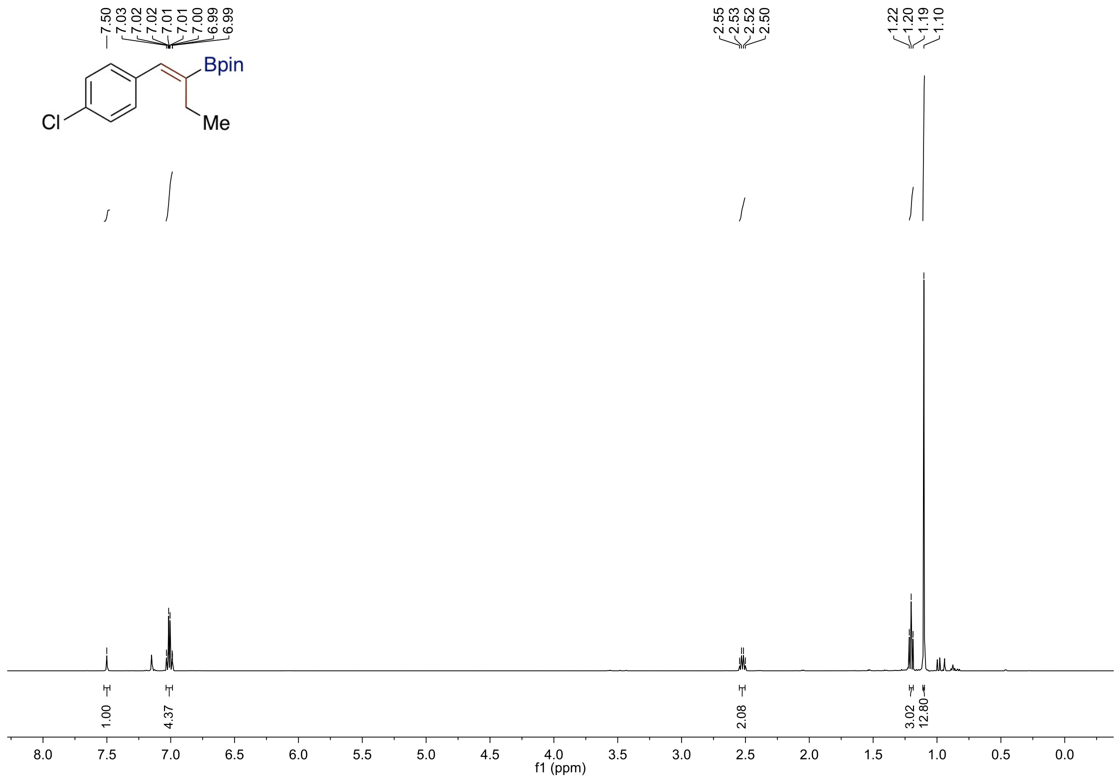
7.50
7.03
7.02
7.02
7.01
7.01
7.00
6.99
6.99
2.55
2.53
2.52
2.50
1.22
1.20
1.19
1.10
Bpin
Cl
Me
1.00
4.37
2.08
3.02
12.80
8.0
7.5
7.0
6.5
6.0
5.5
5.0
4.5
4.0
3.5
3.0
2.5
2.0
1.5
1.0
0.5
0.0
f1 (ppm)

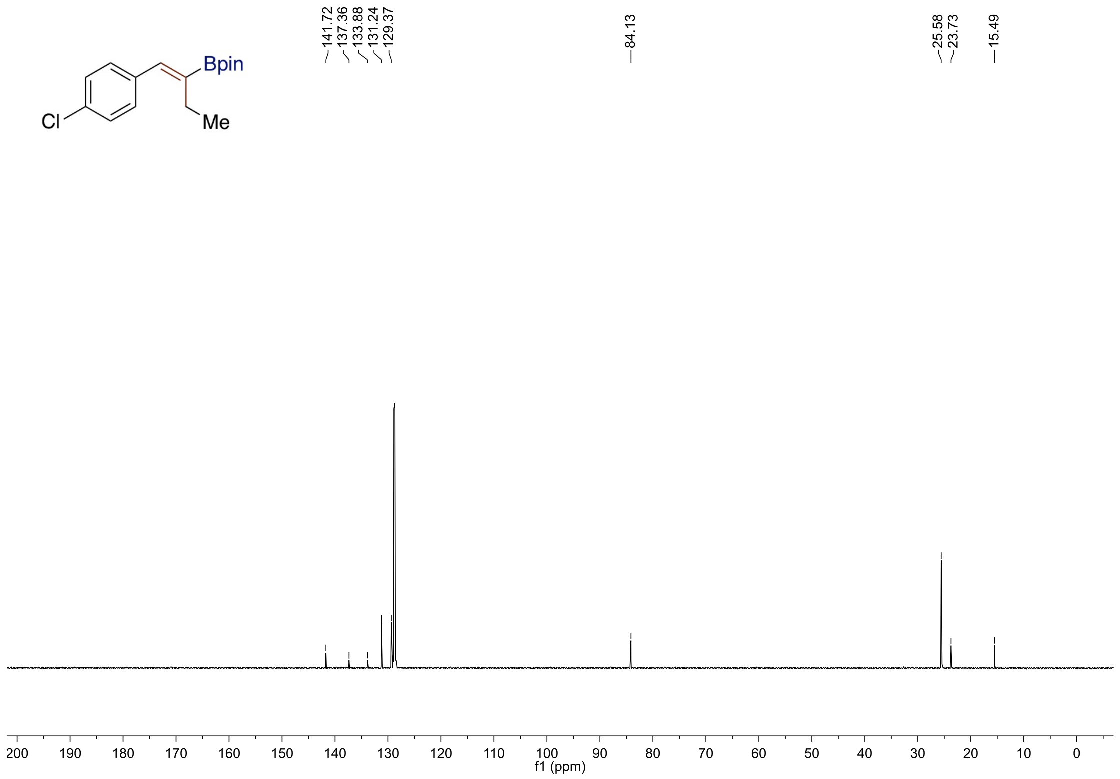
141.72
137.36
133.88
131.24
129.37
84.13
25.58
23.73
15.49
Bpin
Cl
Me
200
190
180
170
160
150
140
130
120
110
100
90
80
70
60
50
40
30
20
10
0
f1 (ppm)

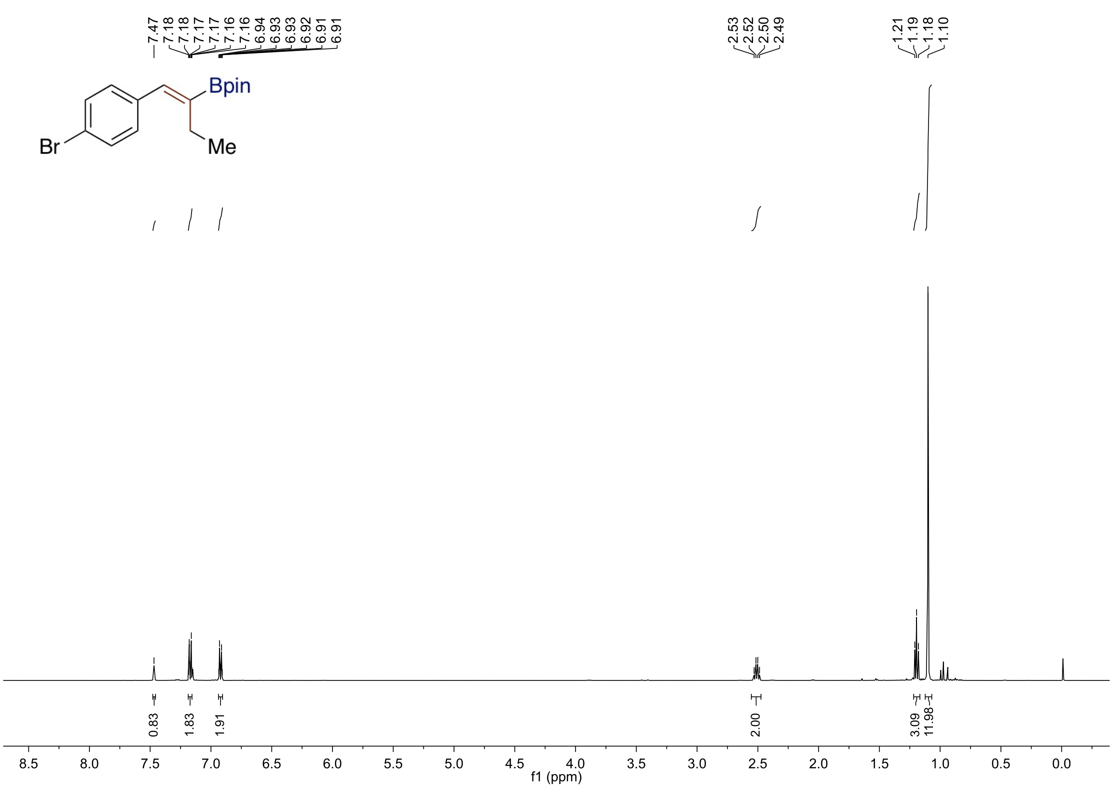

7.47
7.18
7.18
7.17
7.17
7.16
7.16
6.94
6.93
6.93
6.92
6.91
6.91
2.53
2.52
2.50
2.49
1.21
1.19
1.18
1.10
Bpin
Br
Me
0.83
1.83
1.91
2.00
3.09
11.98
8.5 8.0 7.5 7.0 6.5 6.0 5.5 5.0 4.5 4.0 3.5 3.0 2.5 2.0 1.5 1.0 0.5 0.0
f1 (ppm)


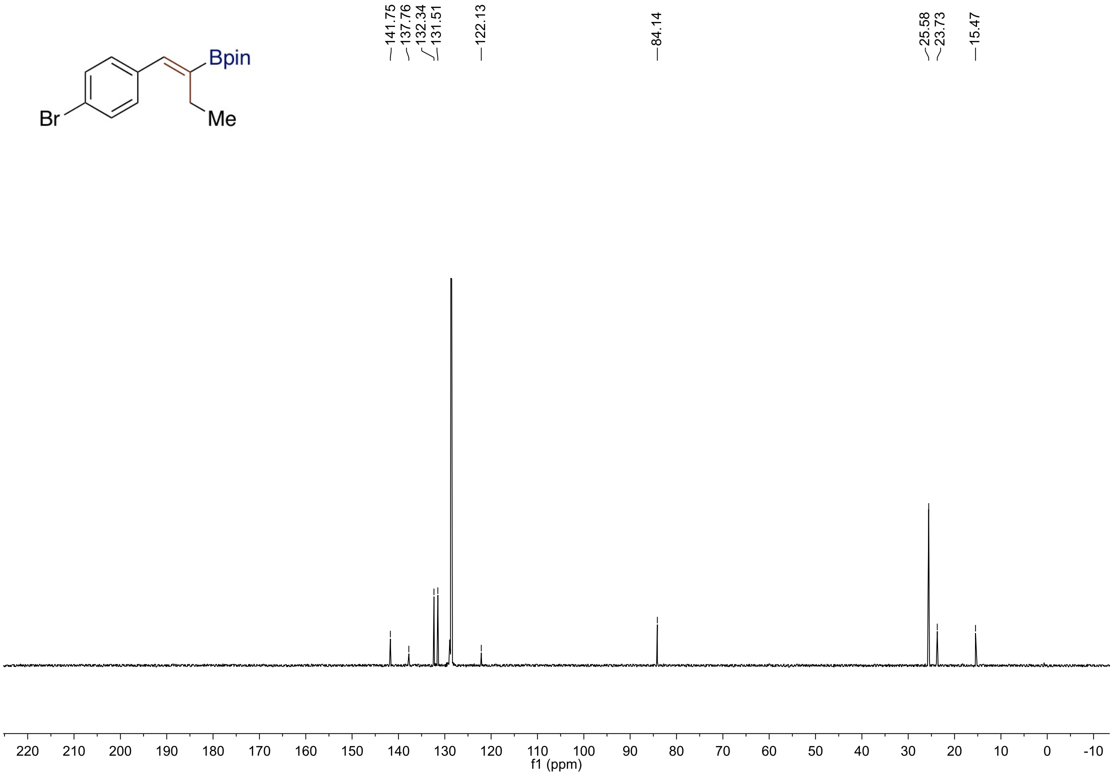

141.75
137.76
132.34
131.51
122.13
84.14
25.58
23.73
15.47
Bpin
Br
Me
220 210 200 190 180 170 160 150 140 130 120 110 100 90 80 70 60 50 40 30 20 10 0 -10
f1 (ppm)

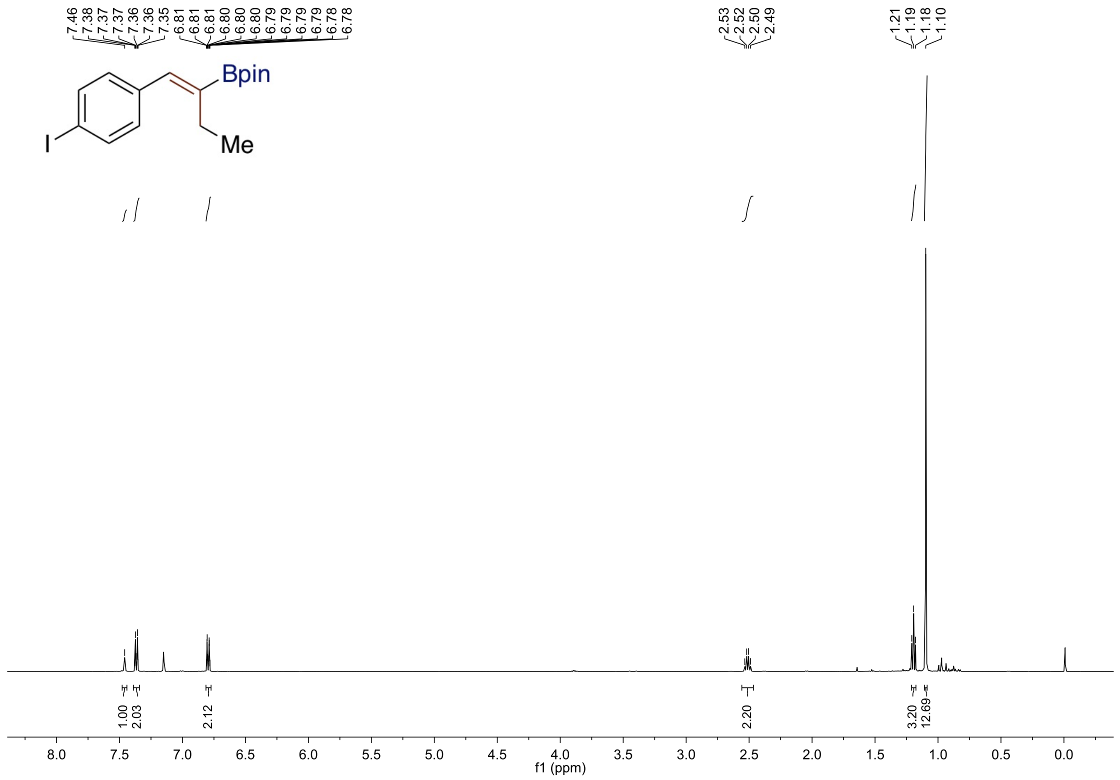
7.46
7.38
7.37
7.37
7.36
7.36
7.35
6.81
6.81
6.80
6.80
6.79
6.79
6.79
6.78
6.78
2.53
2.52
2.50
2.49
1.21
1.19
1.18
1.10
Bpin
I
Me
1.00
2.03
2.12
2.20
3.20
12.69
f1 (ppm)

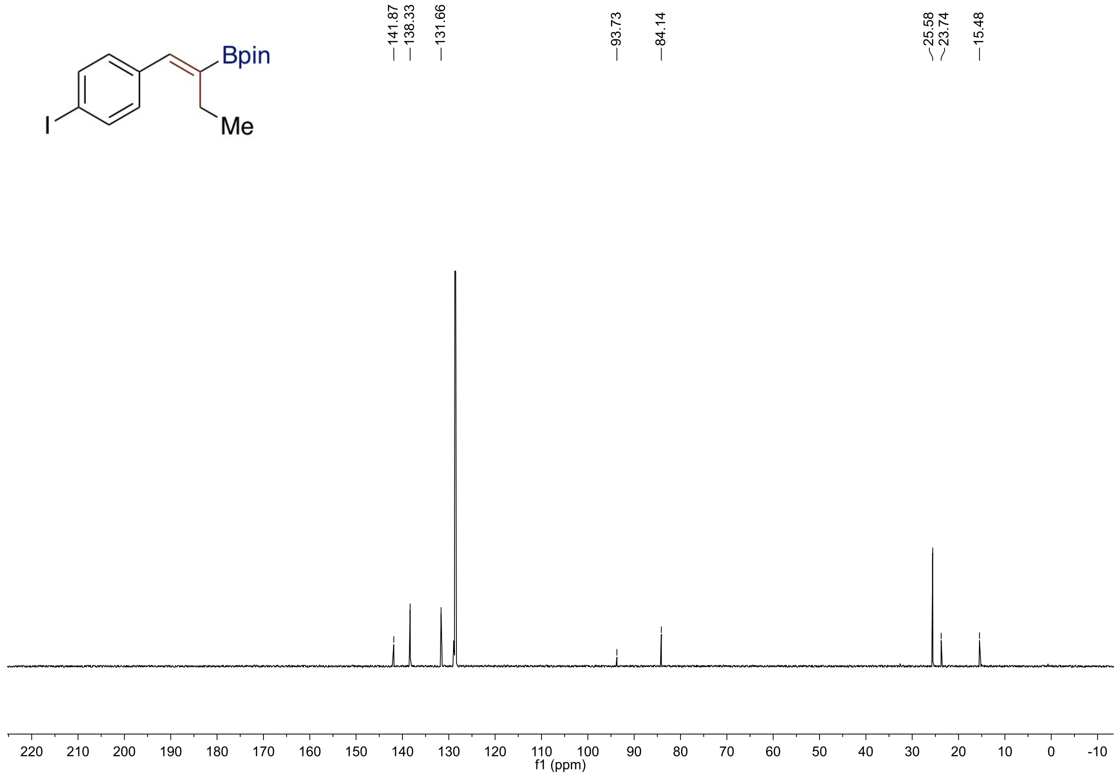
141.87
138.33
131.66
93.73
84.14
25.58
23.74
15.48
Bpin
I
Me
f1 (ppm)

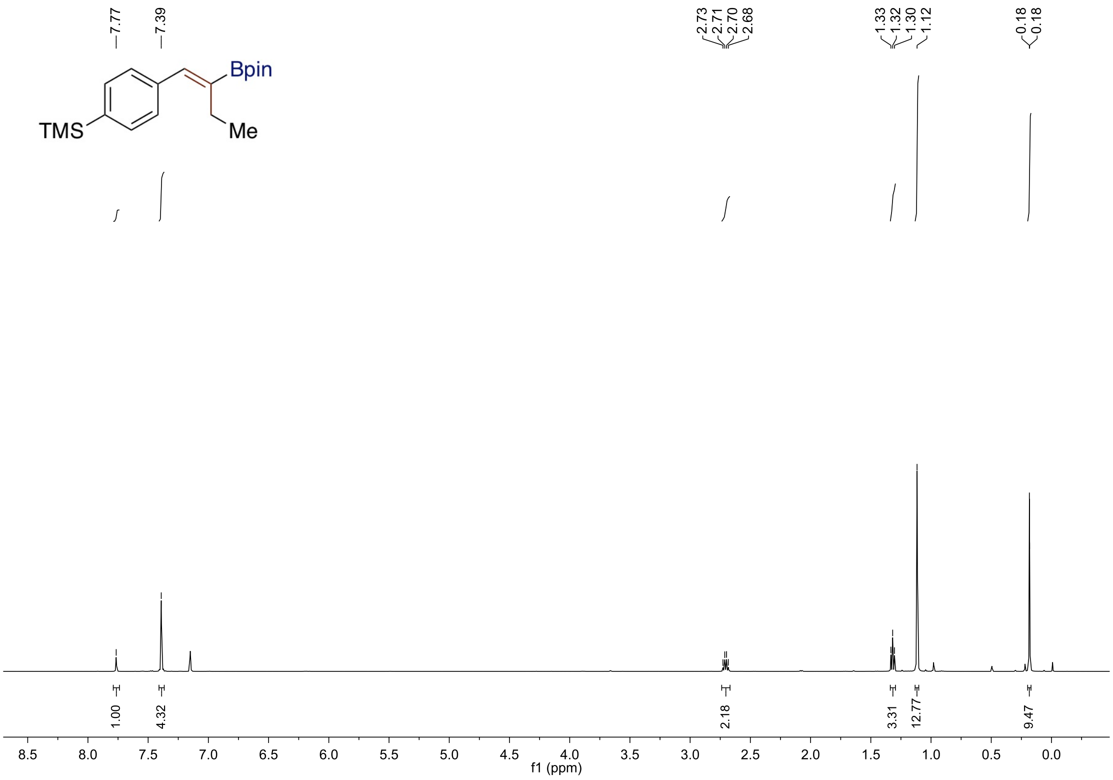
7.77
7.39
2.73
2.71
2.70
2.68
1.33
1.32
1.30
1.12
0.18
0.18
Bpin
TMS
Me
1.00
4.32
2.18
3.31
12.77
9.47
8.5 8.0 7.5 7.0 6.5 6.0 5.5 5.0 4.5 4.0 3.5 3.0 2.5 2.0 1.5 1.0 0.5 0.0
f1 (ppm)

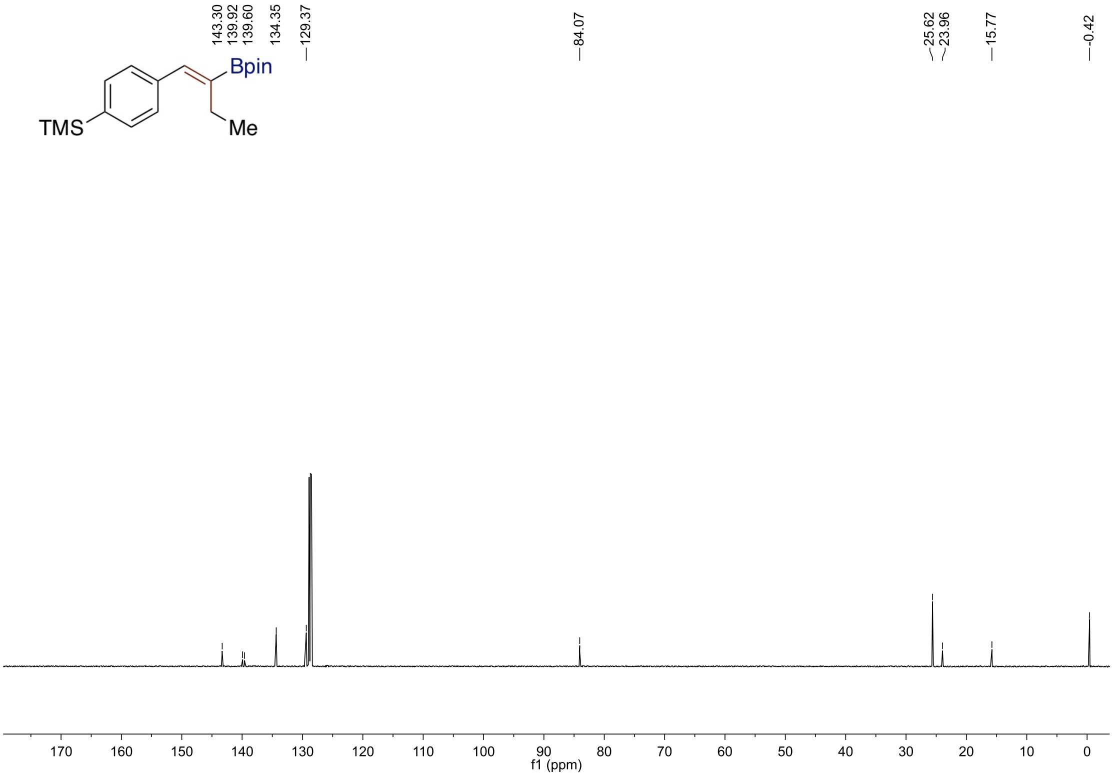
143.30
139.92
139.60
134.35
129.37
84.07
25.62
23.96
15.77
0.42
Bpin
TMS
Me
170 160 150 140 130 120 110 100 90 80 70 60 50 40 30 20 10 0
f1 (ppm)

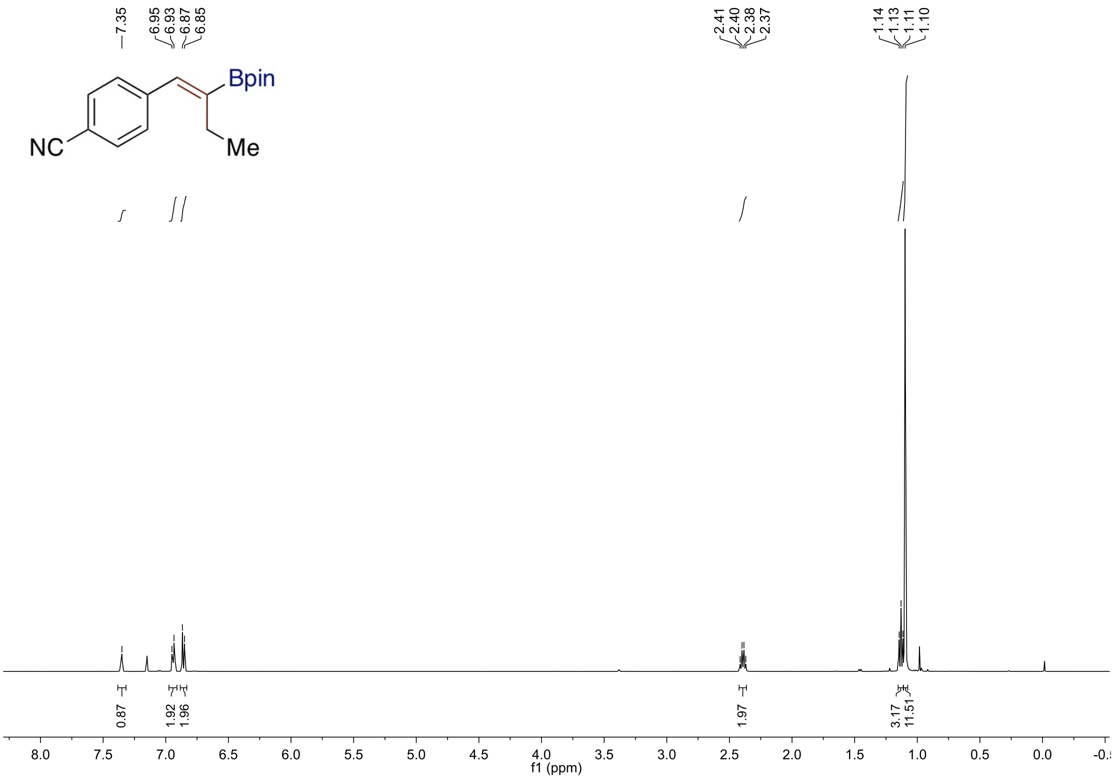
7.35
6.95
6.93
6.87
6.85
2.41
2.40
2.38
2.37
1.14
1.13
1.11
1.10
Bpin
NC
Me
0.87
1.92
1.96
1.97
3.17
11.51
f1 (ppm)

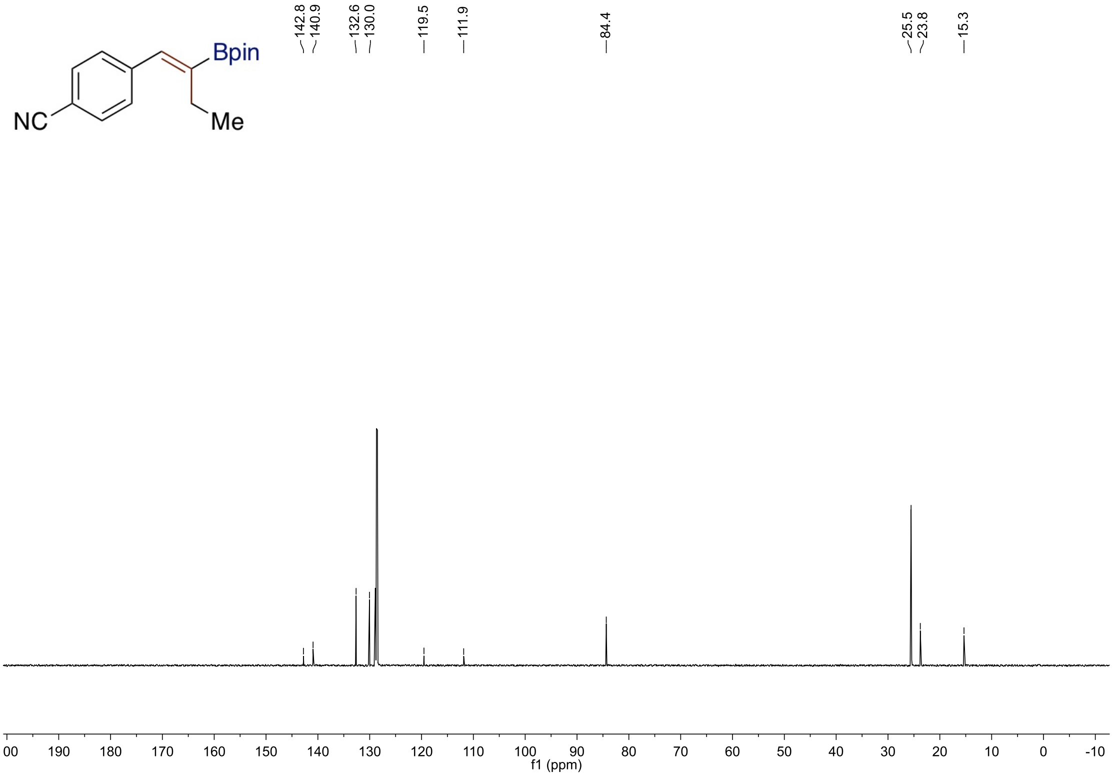
142.8
140.9
132.6
130.0
119.5
111.9
84.4
25.5
23.8
15.3
Bpin
NC
Me
f1 (ppm)

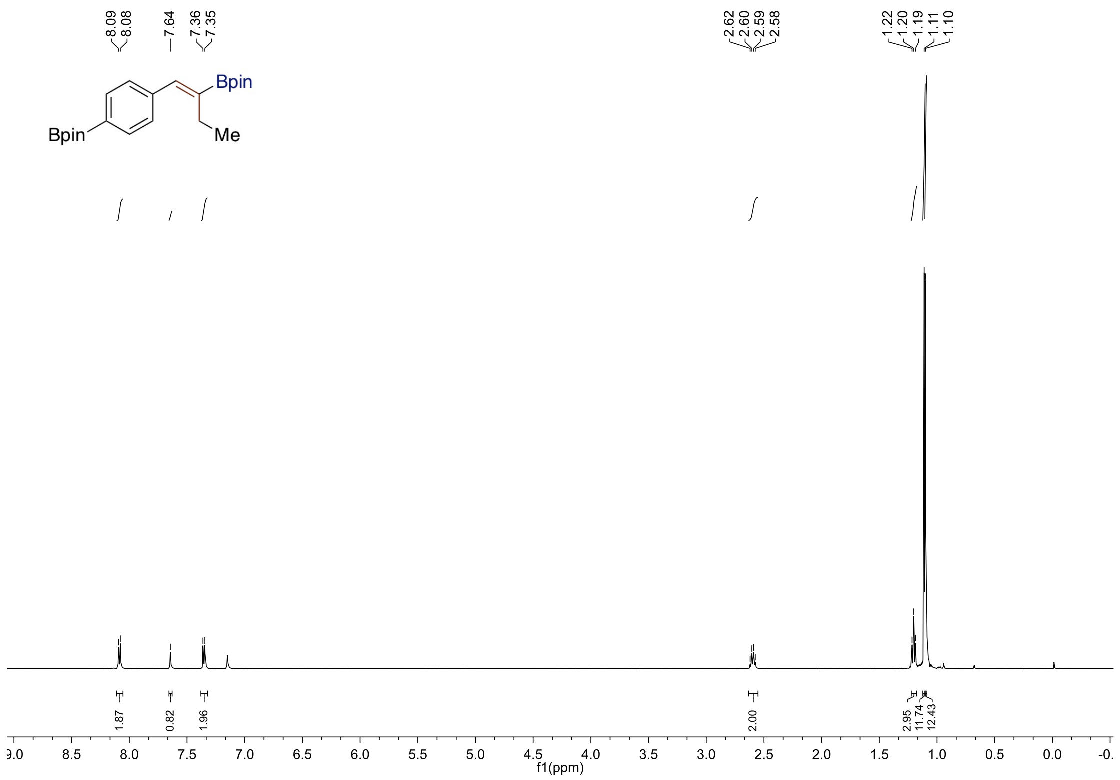

Bpin
Bpin
Me
8.09
8.08
7.64
7.36
7.35
2.62
2.60
2.59
2.58
1.22
1.20
1.19
1.11
1.10
1.87
0.82
1.96
2.00
2.95
11.74
12.43
f1(ppm)


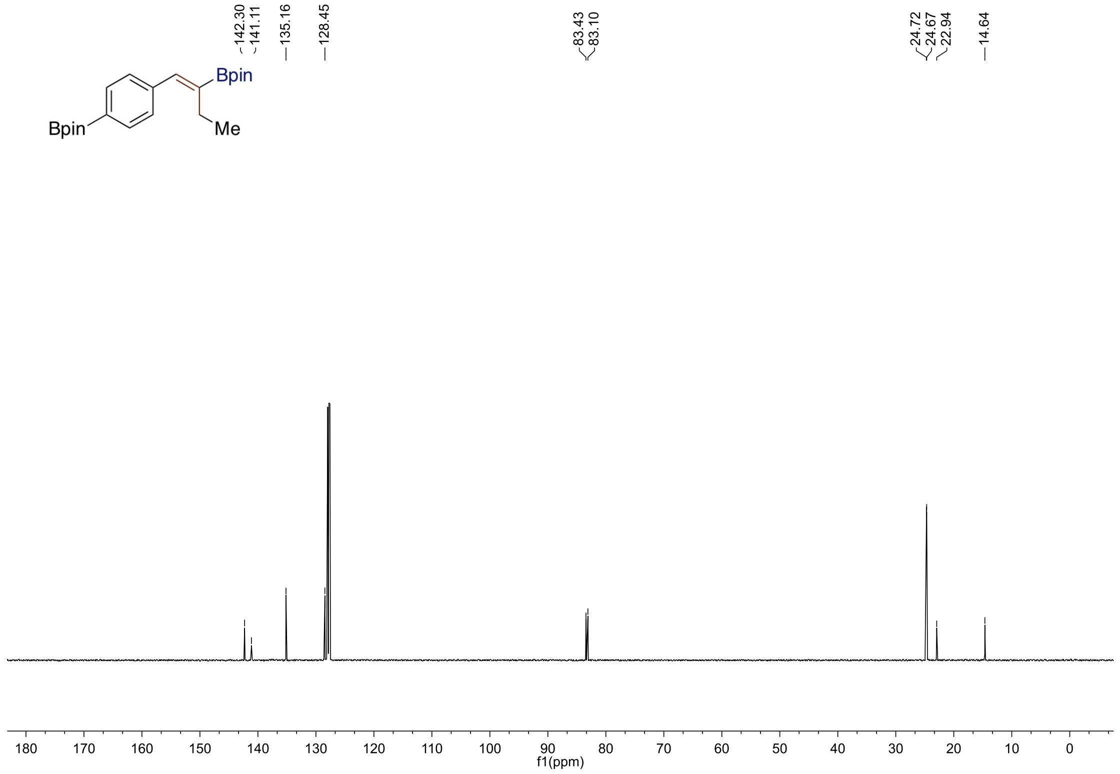

Bpin
Bpin
Me
142.30
141.11
135.16
128.45
83.43
83.10
24.72
24.67
22.94
14.64
f1(ppm)

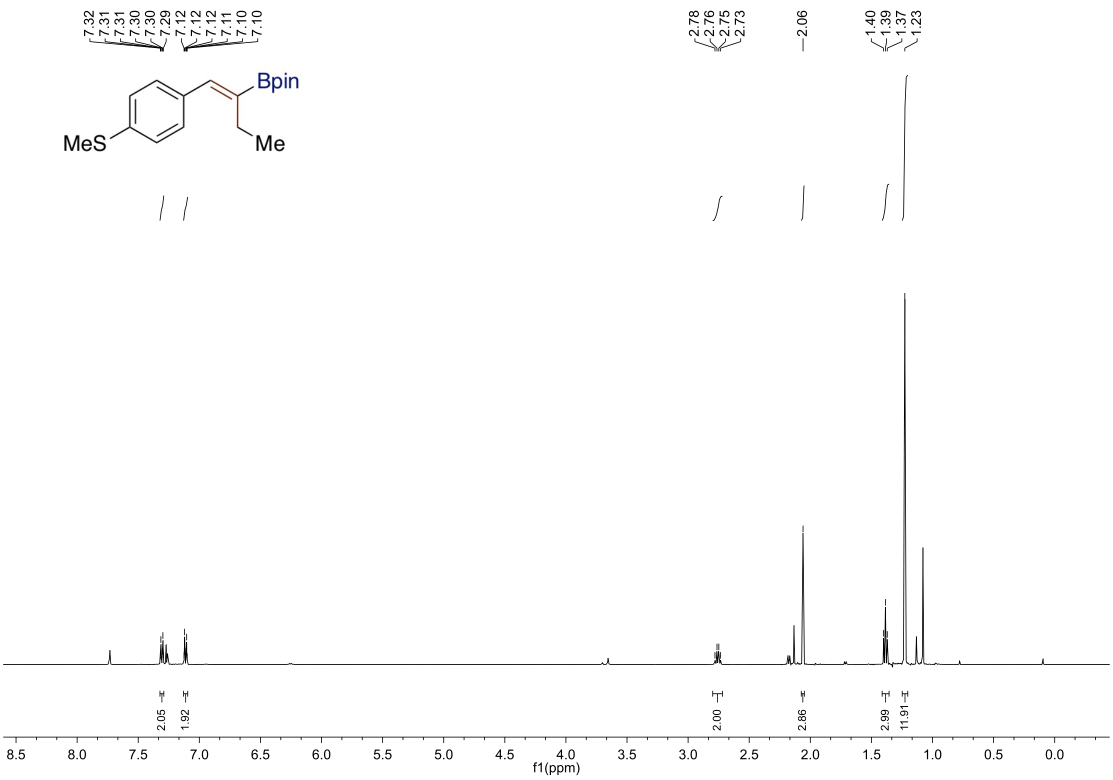
7.32
7.31
7.31
7.30
7.30
7.29
7.12
7.12
7.12
7.11
7.10
7.10
2.78
2.76
2.75
2.73
2.06
1.40
1.39
1.37
1.23
Bpin
MeS
Me
2.05
1.92
2.00
2.86
2.99
11.91
f1(ppm)

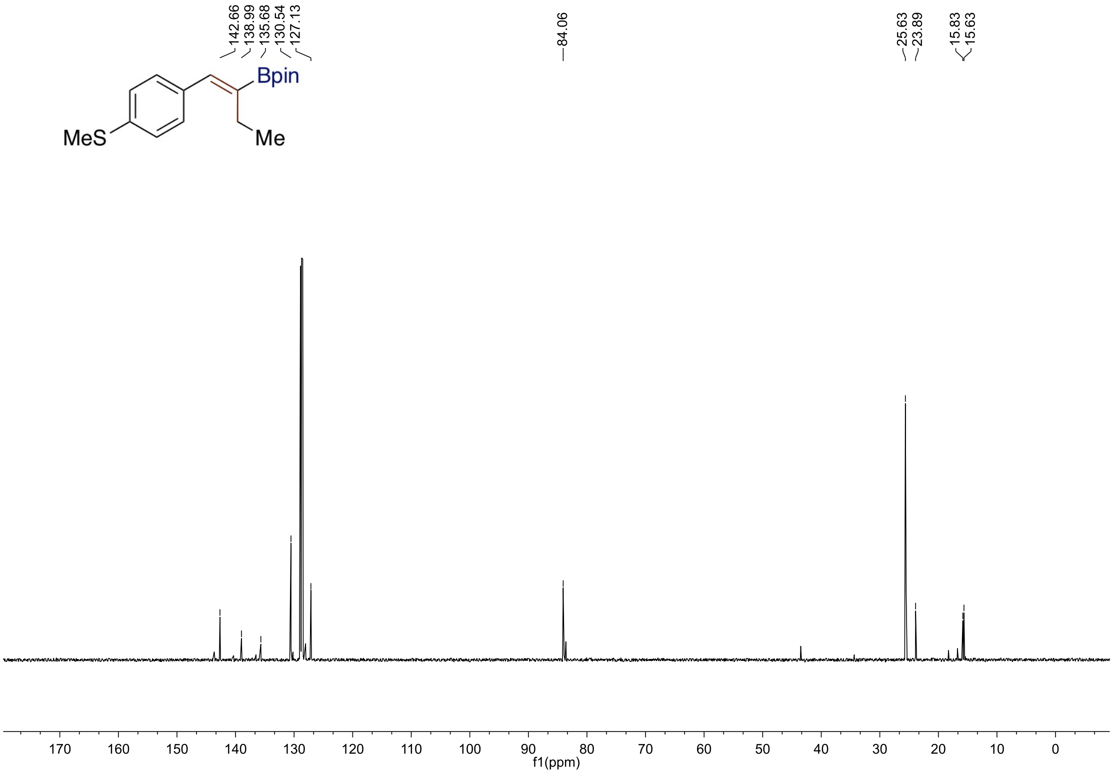
142.66
138.99
135.68
130.54
127.13
84.06
25.63
23.89
15.83
15.63
Bpin
MeS
Me
f1(ppm)

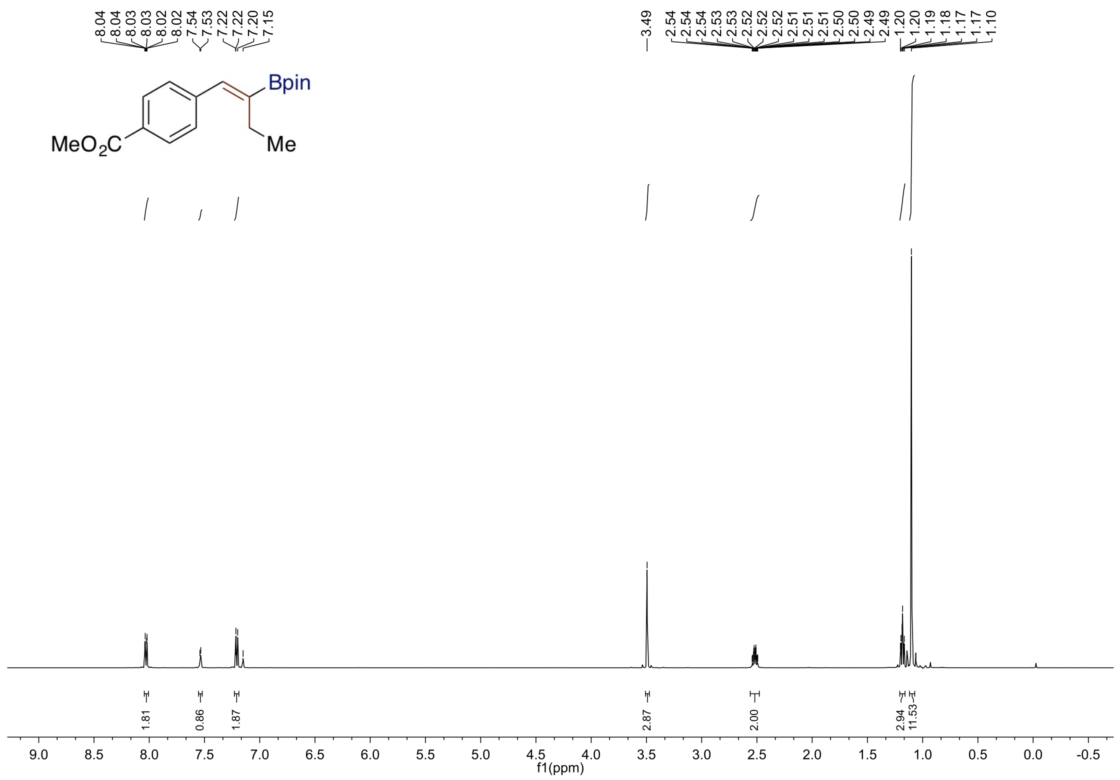
Bpin
MeO2C
Me
f1(ppm)

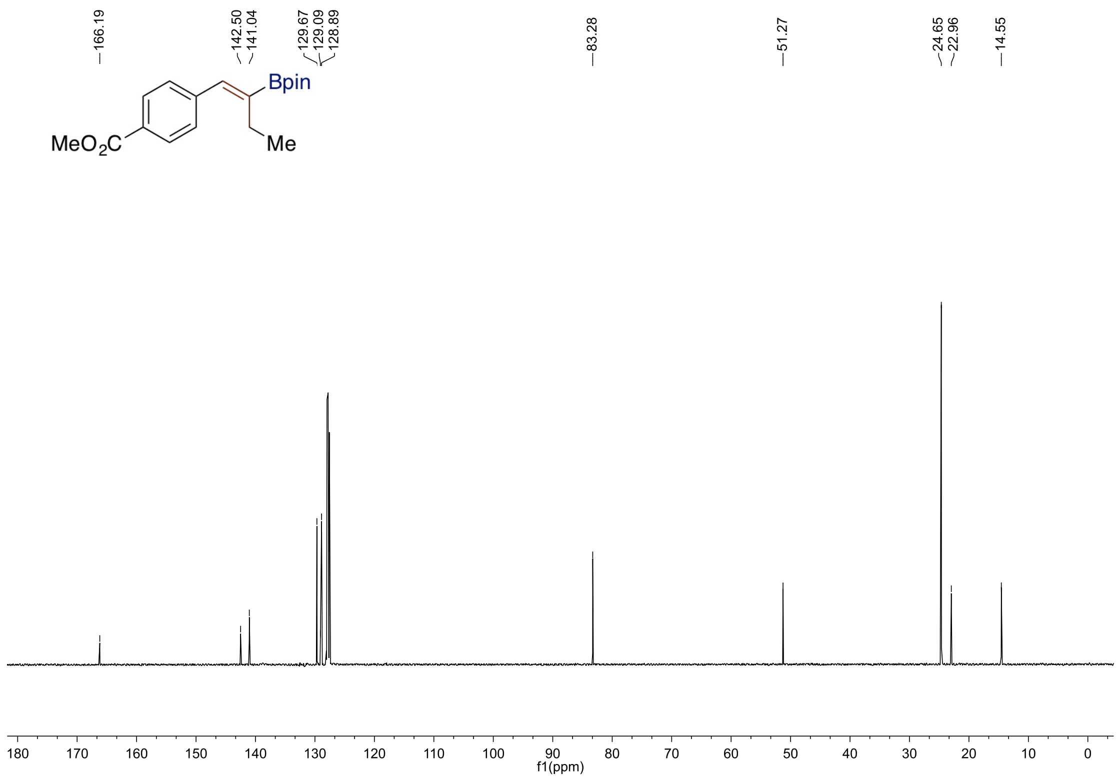
Bpin
MeO2C
Me
f1(ppm)

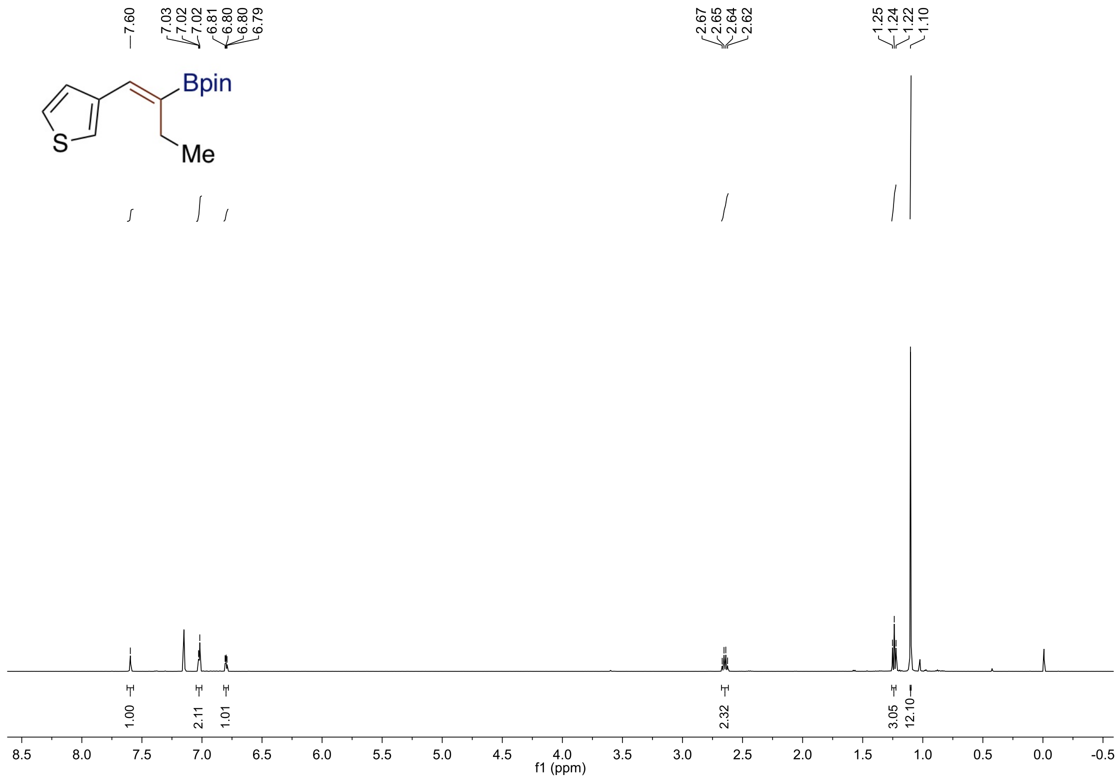
7.60
7.03
7.02
7.02
6.81
6.80
6.80
6.79
2.67
2.65
2.64
2.62
1.25
1.24
1.22
1.10
Bpin
S
Me
1.00
2.11
1.01
2.32
3.05
12.10
8.5
8.0
7.5
7.0
6.5
6.0
5.5
5.0
4.5
4.0
3.5
3.0
2.5
2.0
1.5
1.0
0.5
0.0
-0.5
f1 (ppm)

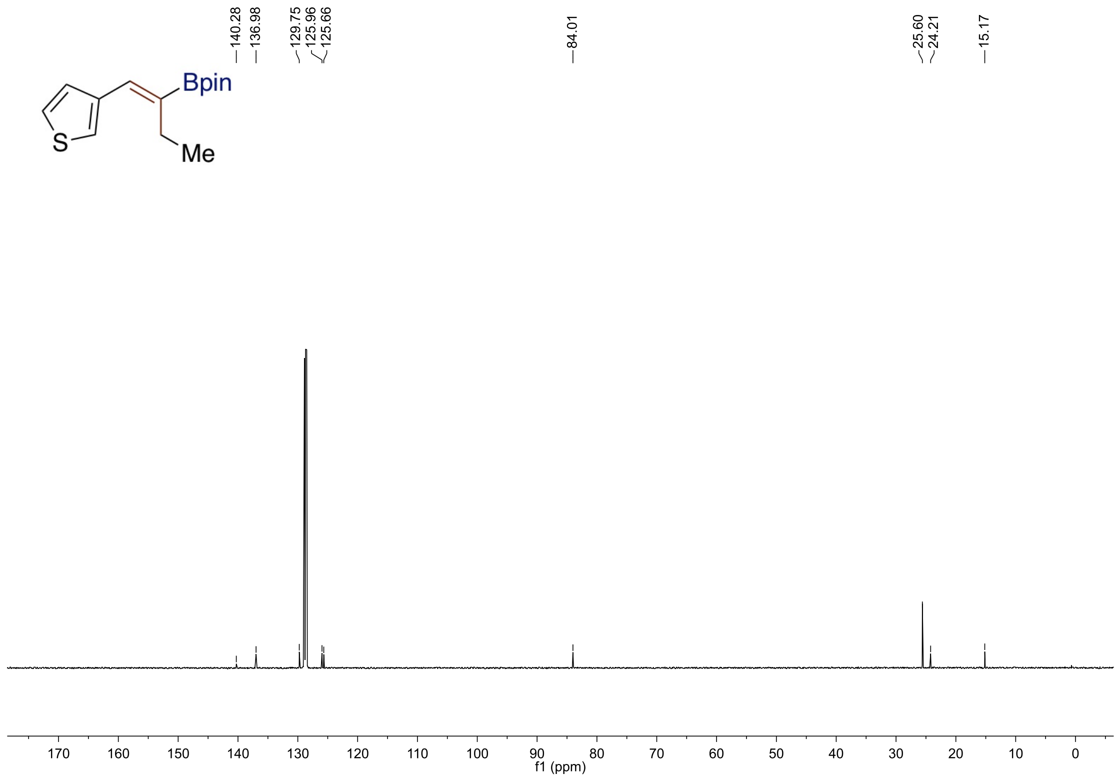
140.28
136.98
129.75
125.96
125.66
84.01
25.60
24.21
15.17
Bpin
S
Me
170
160
150
140
130
120
110
100
90
80
70
60
50
40
30
20
10
0
f1 (ppm)

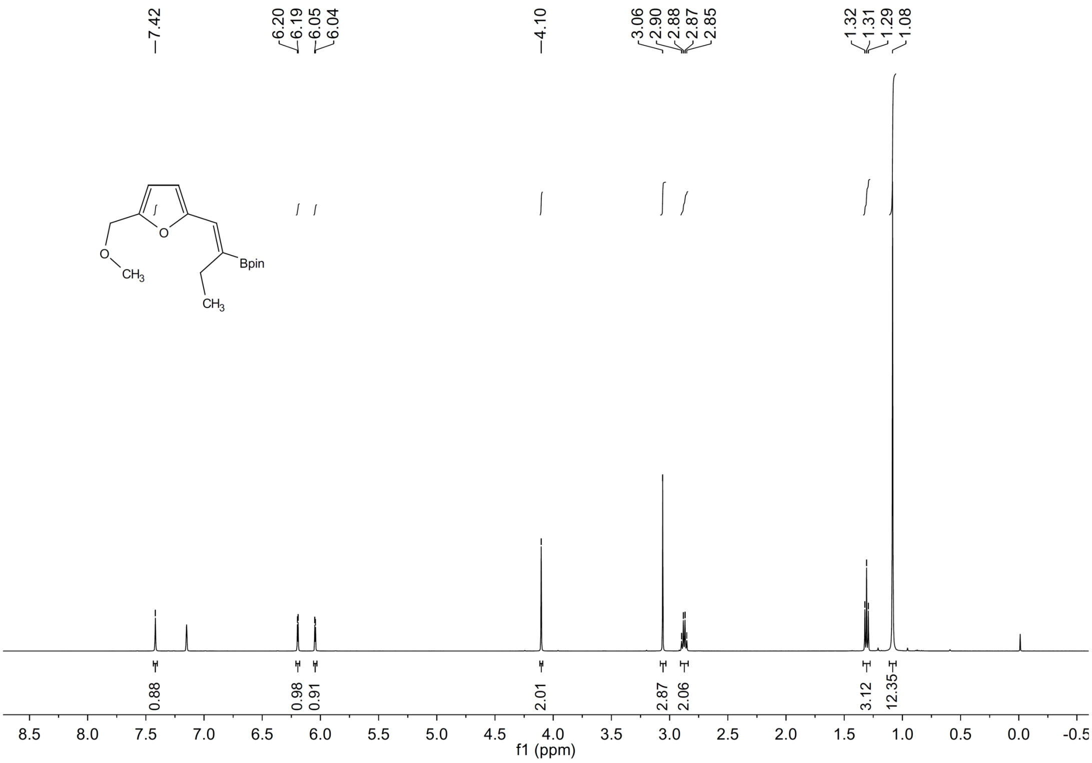
7.42
6.20
6.19
6.05
6.04
4.10
3.06
2.90
2.88
2.87
2.85
1.32
1.31
1.29
1.08
O
CH3
Bpin
CH3
0.88
0.98
0.91
2.01
2.87
2.06
3.12
12.35
f1 (ppm)

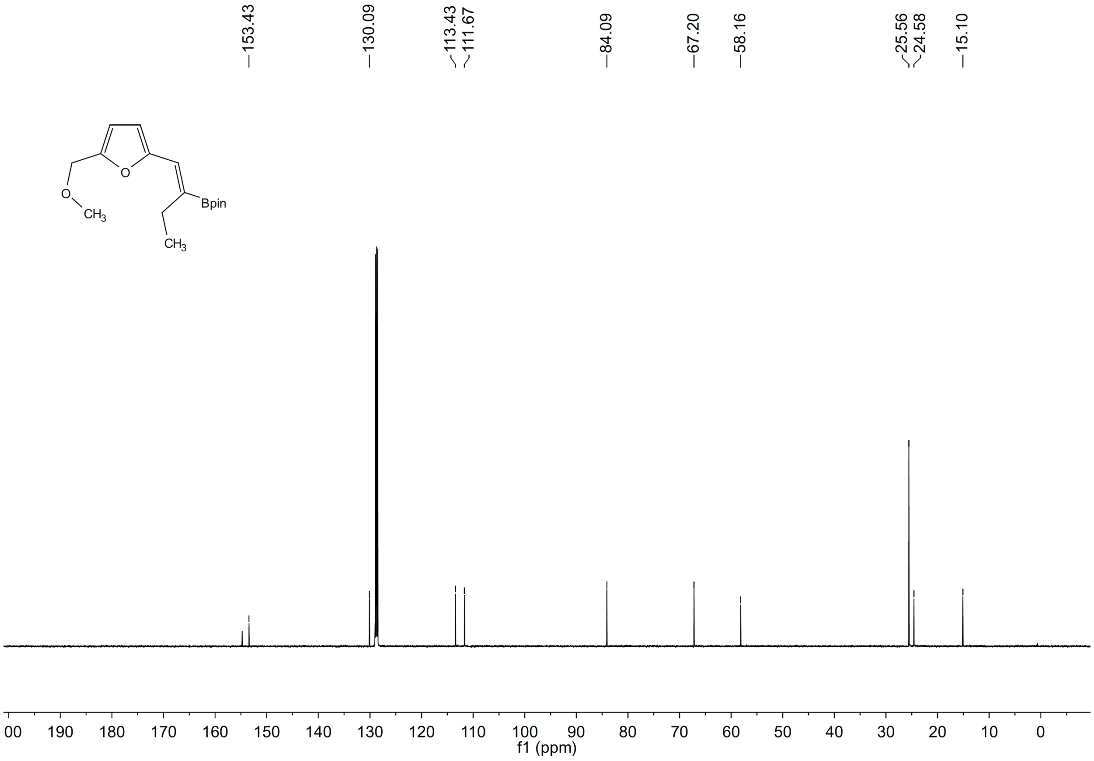
153.43
130.09
113.43
111.67
84.09
67.20
58.16
25.56
24.58
15.10
O
CH3
Bpin
CH3
f1 (ppm)

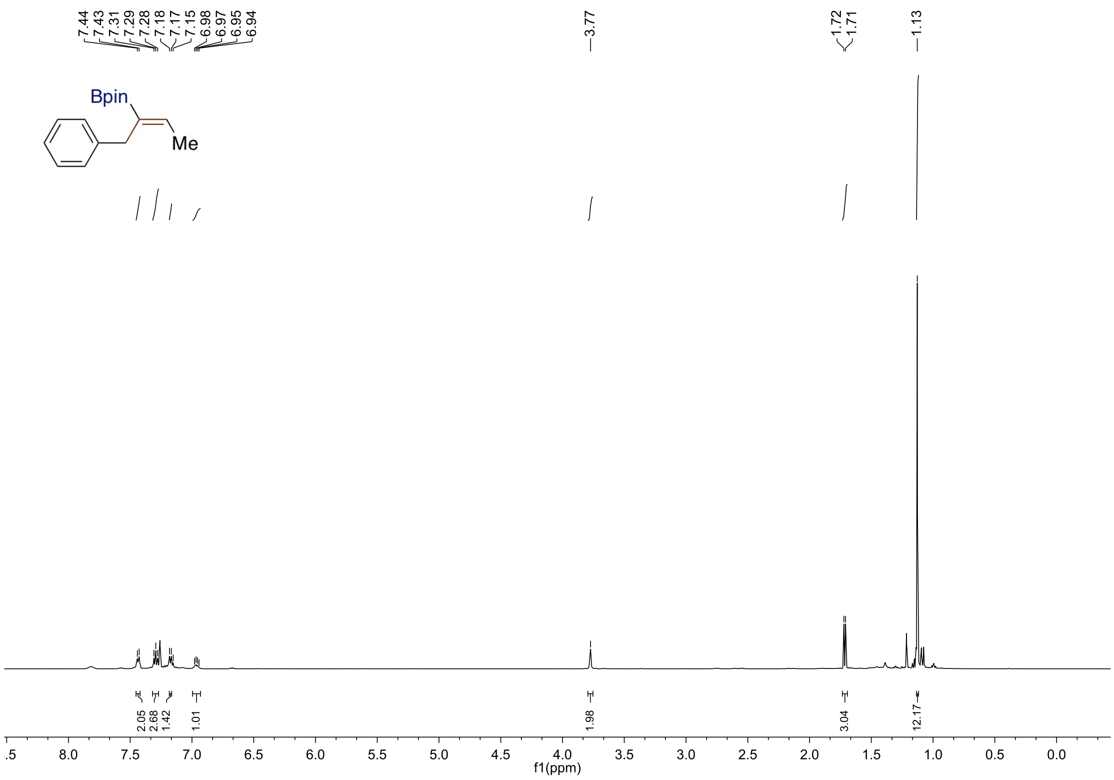
7.44
7.43
7.31
7.29
7.28
7.18
7.17
7.15
6.98
6.97
6.95
6.94
3.77
1.72
1.71
1.13
Bpin
Me
2.05
2.68
1.42
1.01
1.98
3.04
12.17
f1(ppm)

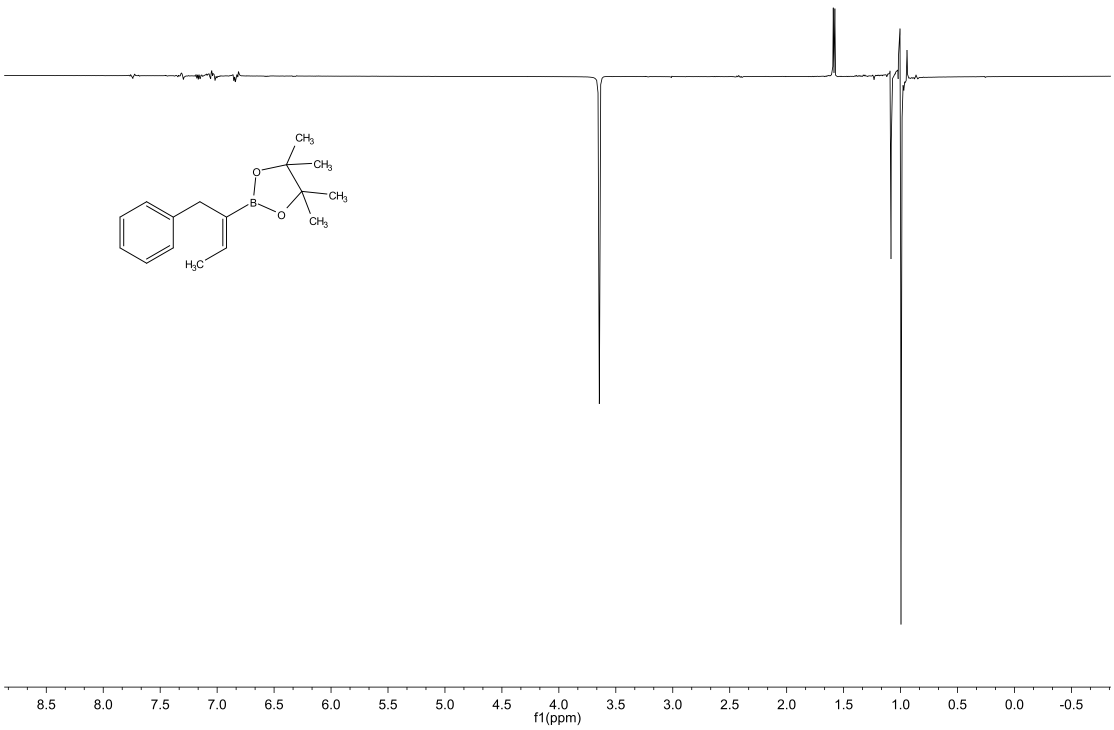
CH3
CH3
CH3
CH3
O
O
B
H3C
f1(ppm)

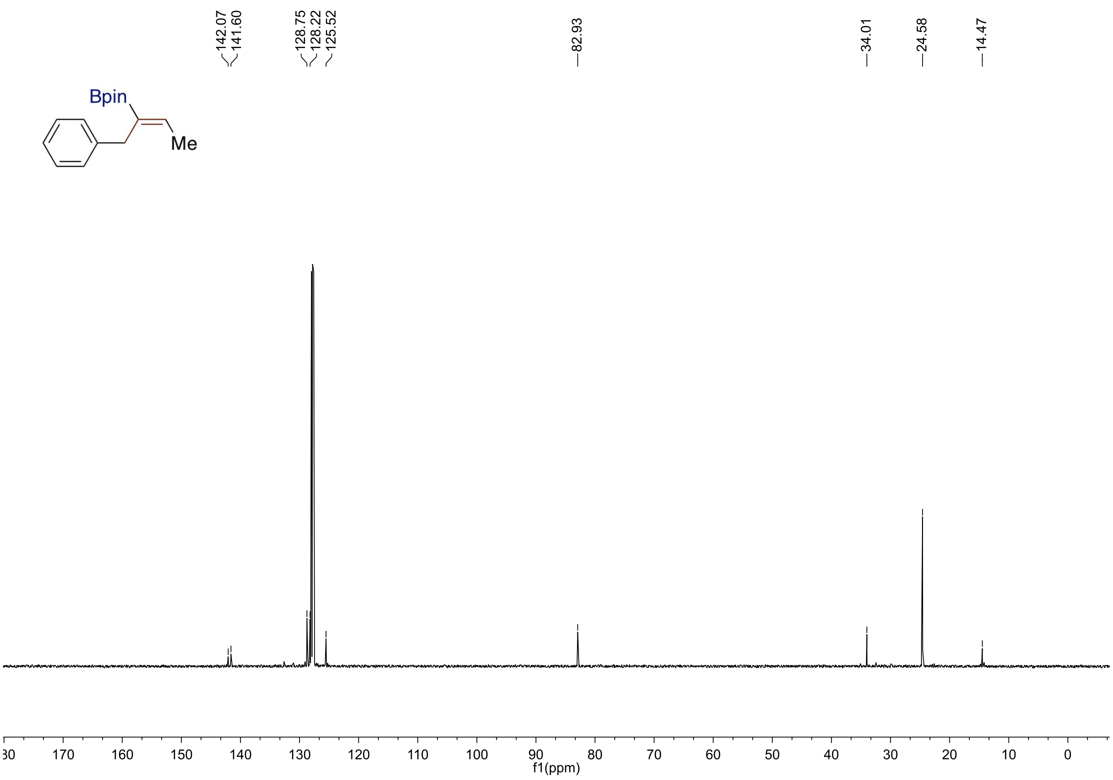
142.07
141.60
128.75
128.22
125.52
82.93
34.01
24.58
14.47
Bpin
Me
180 170 160 150 140 130 120 110 100 90 80 70 60 50 40 30 20 10 0
f1(ppm)

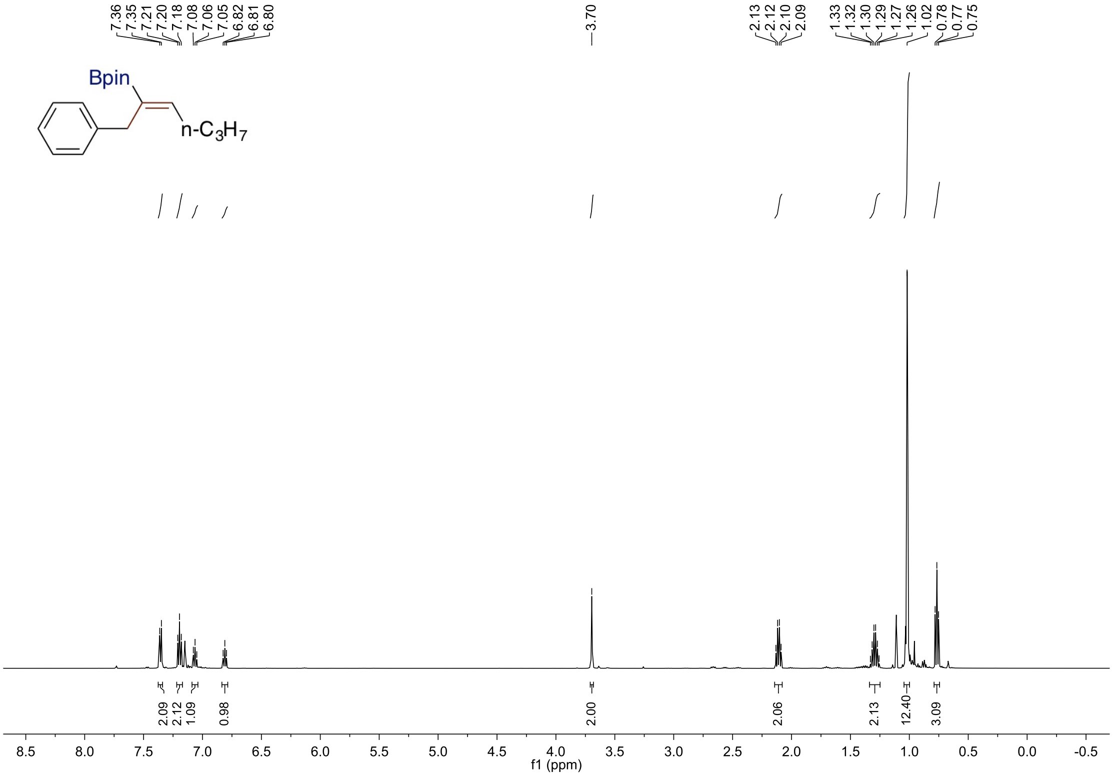
7.36
7.35
7.21
7.20
7.18
7.08
7.06
7.05
6.82
6.81
6.80
3.70
2.13
2.12
2.10
2.09
1.33
1.32
1.30
1.29
1.27
1.26
1.02
0.78
0.77
0.75
Bpin
n-C3H7
2.09
2.12
1.09
0.98
2.00
2.06
2.13
12.40
3.09
8.5 8.0 7.5 7.0 6.5 6.0 5.5 5.0 4.5 4.0 3.5 3.0 2.5 2.0 1.5 1.0 0.5 0.0 -0.5
f1 (ppm)

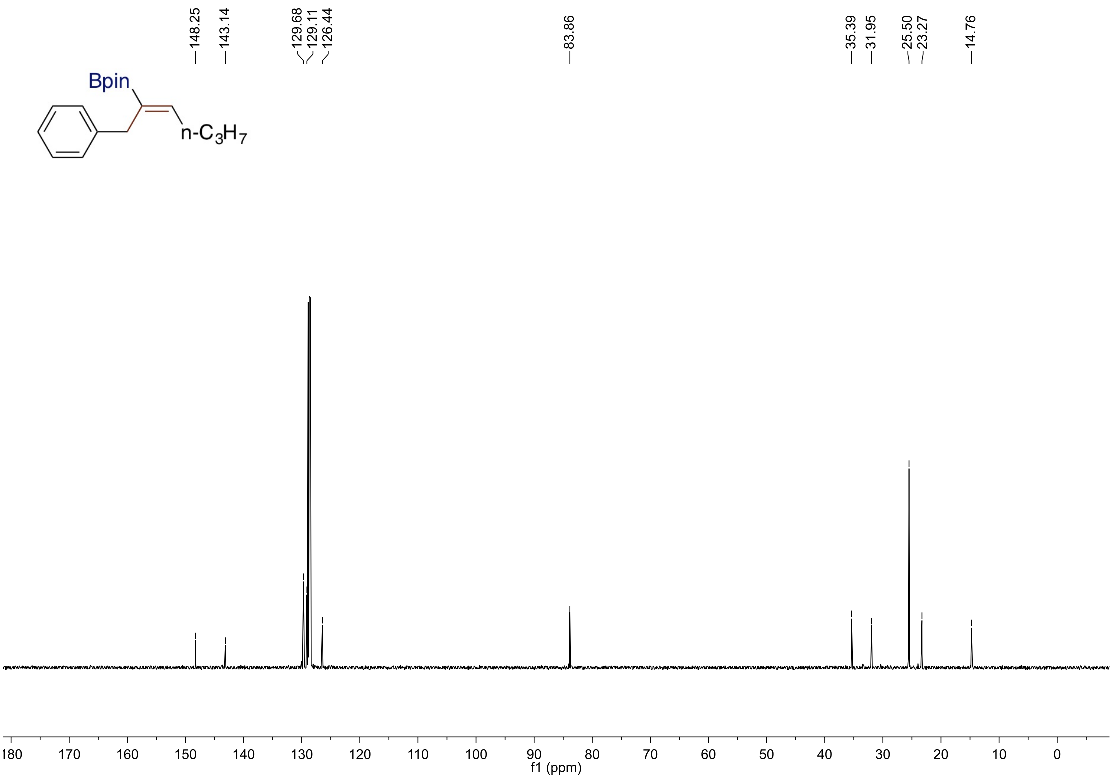

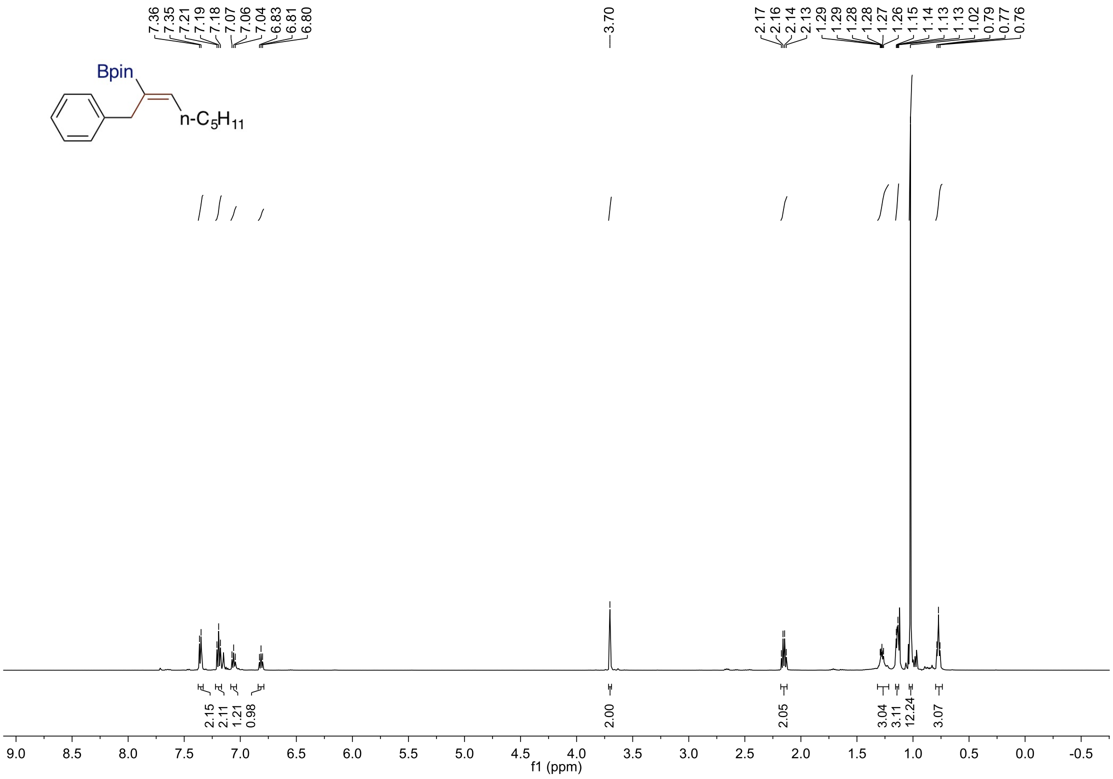

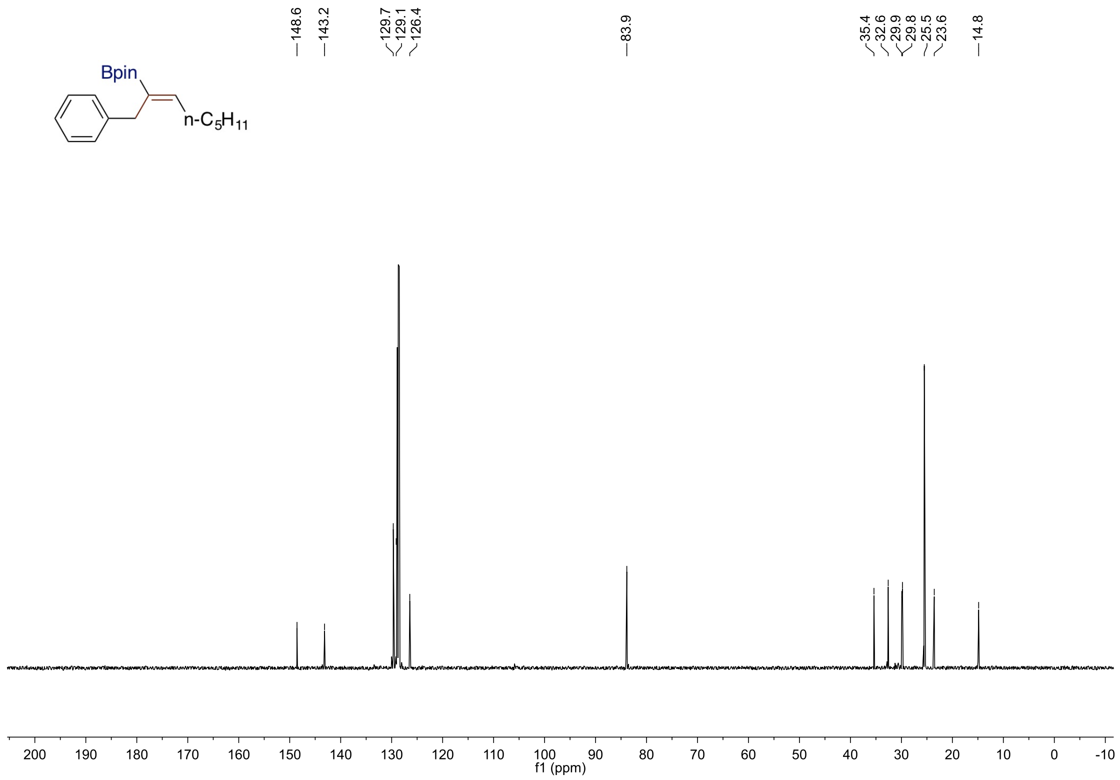
148.6
143.2
129.7
129.1
126.4
83.9
35.4
32.6
29.9
29.8
25.5
23.6
14.8
Bpin
n-C5H11
200 190 180 170 160 150 140 130 120 110 100 90 80 70 60 50 40 30 20 10 0 -10
f1 (ppm)

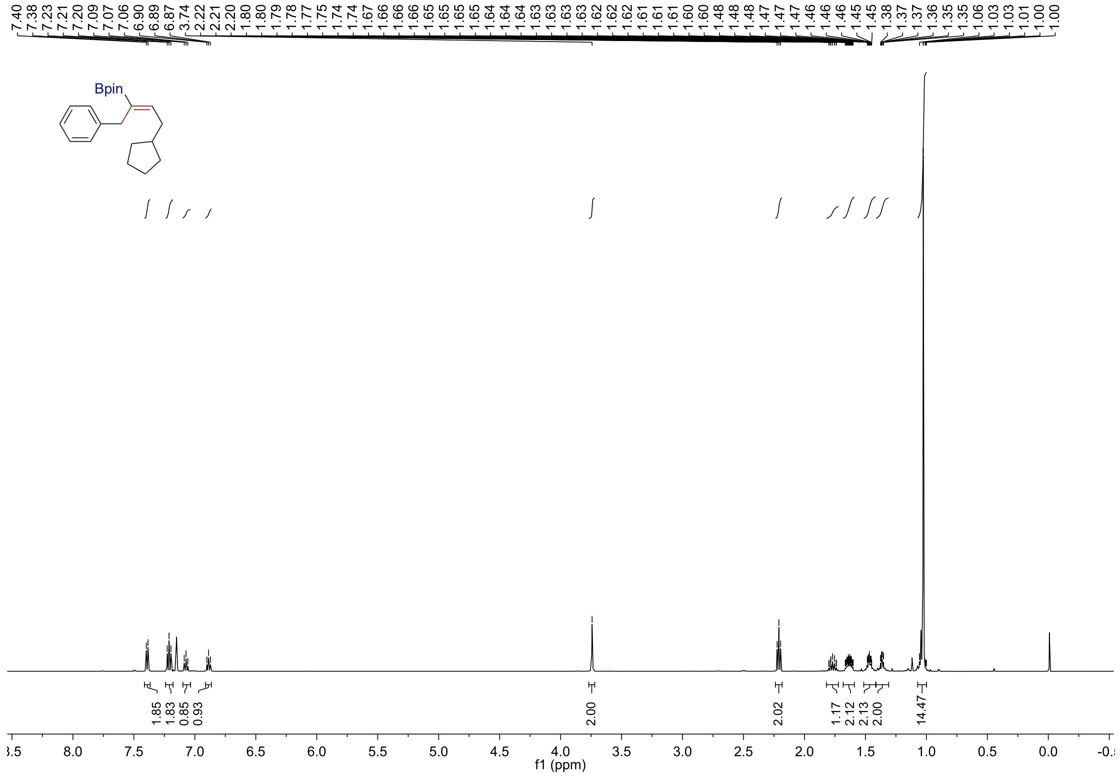
7.40
7.38
7.23
7.21
7.20
7.09
7.07
7.06
6.90
6.89
6.87
3.74
2.22
2.21
2.20
1.80
1.80
1.79
1.78
1.77
1.75
1.74
1.74
1.67
1.66
1.66
1.66
1.65
1.65
1.65
1.64
1.64
1.64
1.63
1.63
1.63
1.62
1.62
1.62
1.61
1.61
1.61
1.60
1.60
1.48
1.48
1.47
1.47
1.46
1.46
1.46
1.45
1.45
1.38
1.37
1.37
1.36
1.35
1.35
1.06
1.03
1.03
1.01
1.00
1.00
Bpin
1.85
1.83
0.85
0.93
2.00
2.02
1.17
2.12
2.13
2.00
14.47
8.0 7.5 7.0 6.5 6.0 5.5 5.0 4.5 4.0 3.5 3.0 2.5 2.0 1.5 1.0 0.5 0.0
f1 (ppm)

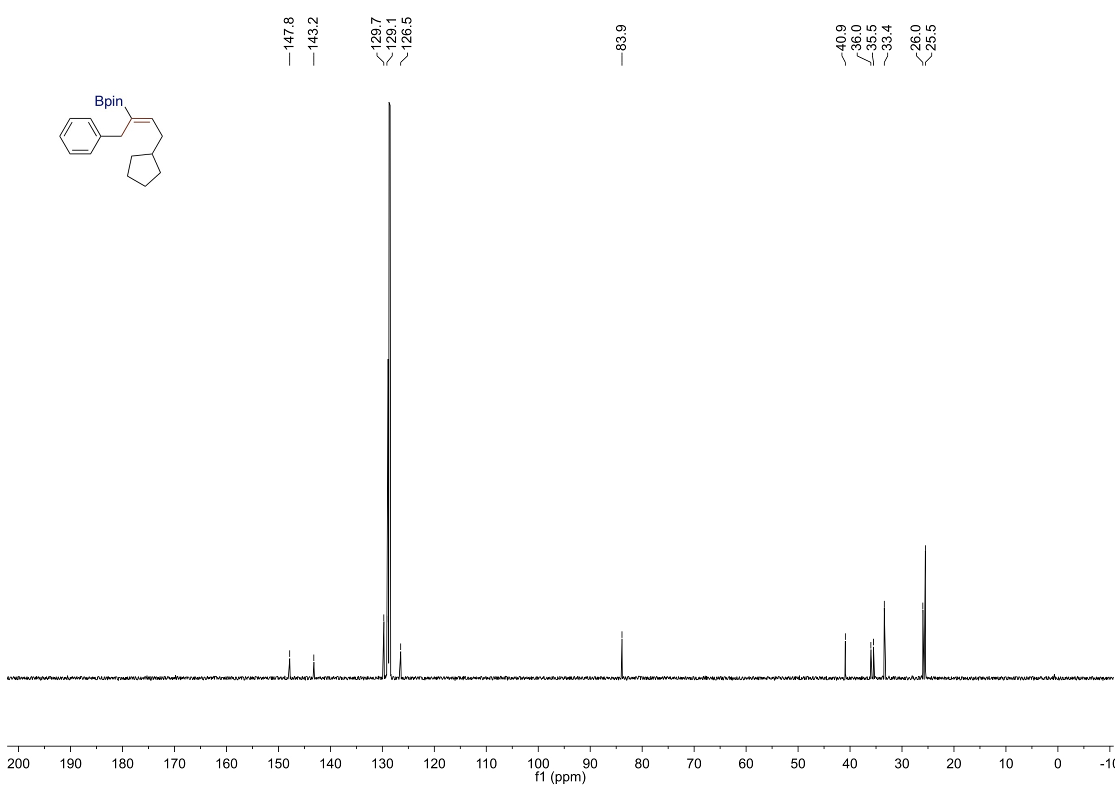
147.8
143.2
129.7
129.1
126.5
83.9
40.9
36.0
35.5
33.4
26.0
25.5
Bpin
200 190 180 170 160 150 140 130 120 110 100 90 80 70 60 50 40 30 20 10 0
f1 (ppm)

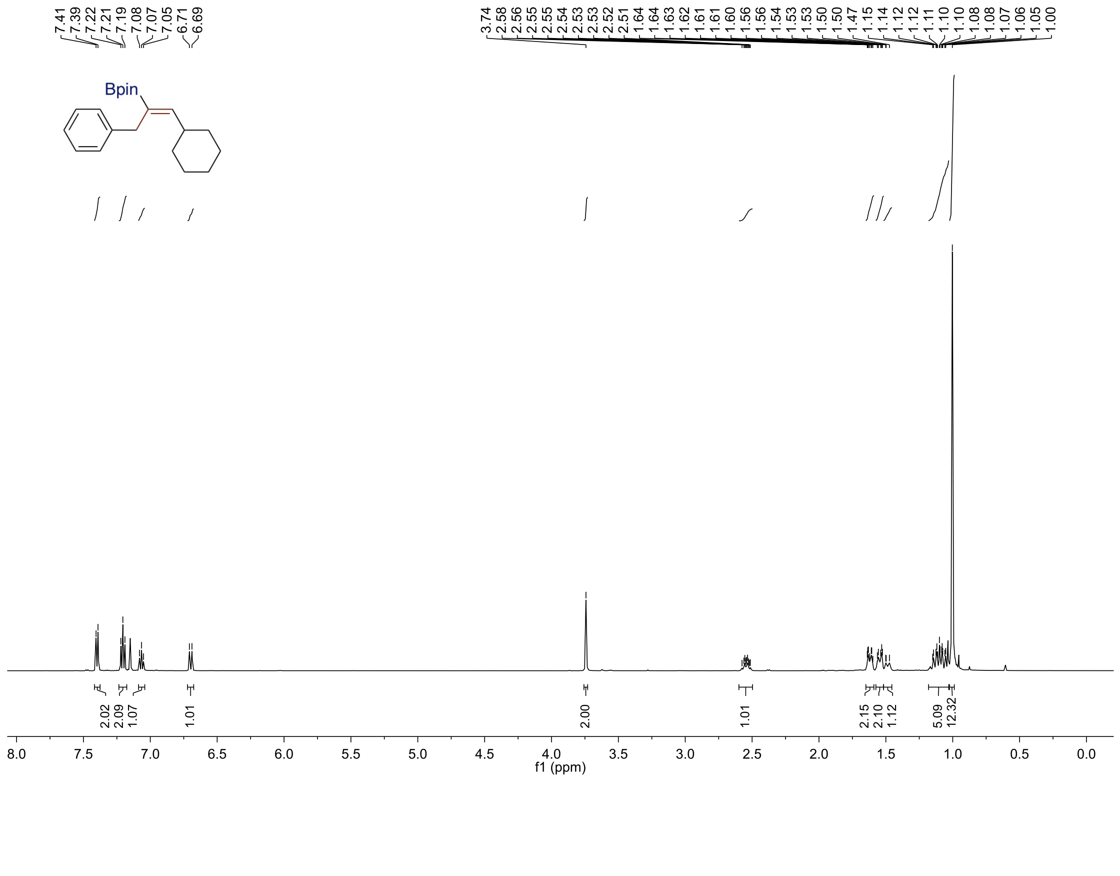
7.41
7.39
7.22
7.21
7.19
7.08
7.07
7.05
6.71
6.69
3.74
2.58
2.56
2.55
2.55
2.54
2.53
2.53
2.52
2.51
1.64
1.64
1.63
1.62
1.61
1.61
1.60
1.56
1.56
1.54
1.53
1.53
1.50
1.50
1.47
1.15
1.14
1.12
1.12
1.11
1.10
1.10
1.08
1.08
1.07
1.06
1.05
1.00
Bpin
2.02
2.09
1.07
1.01
2.00
1.01
2.15
2.10
1.12
5.09
12.32
8.0 7.5 7.0 6.5 6.0 5.5 5.0 4.5 4.0 3.5 3.0 2.5 2.0 1.5 1.0 0.5 0.0
f1 (ppm)

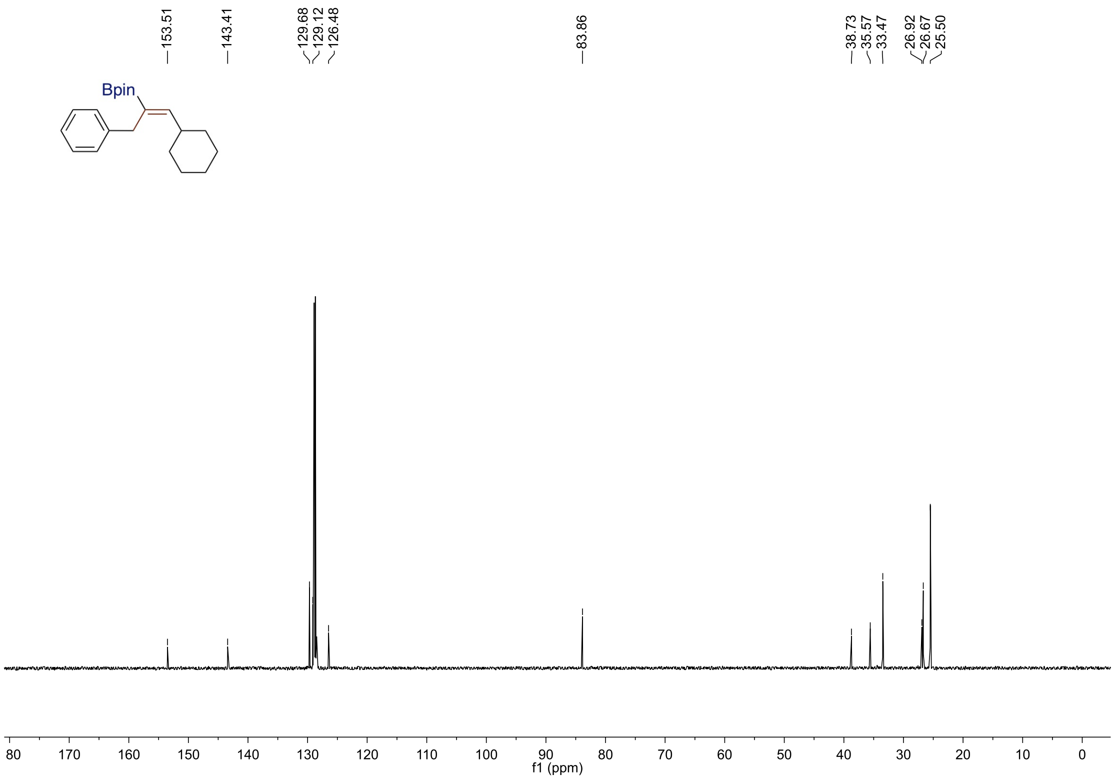
153.51
143.41
129.68
129.12
126.48
83.86
38.73
35.57
33.47
26.92
26.67
25.50
Bpin
f1 (ppm)

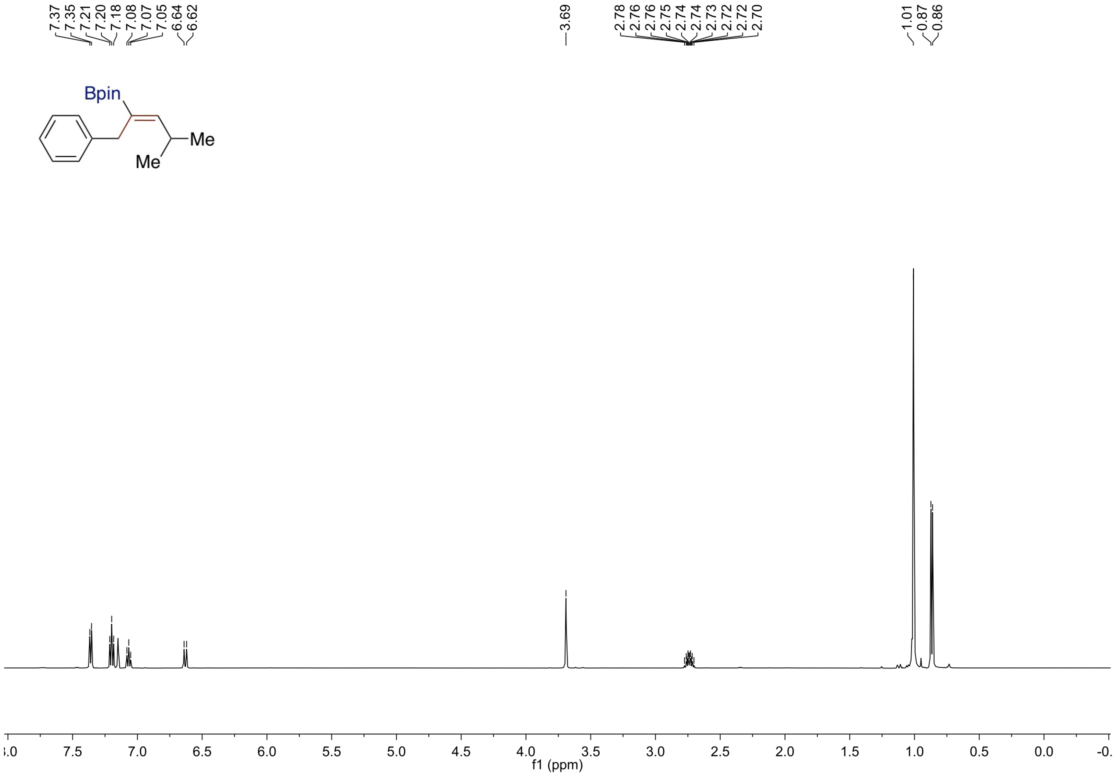
7.37
7.35
7.21
7.20
7.18
7.08
7.07
7.05
6.64
6.62
3.69
2.78
2.76
2.76
2.75
2.74
2.74
2.73
2.72
2.72
2.70
1.01
0.87
0.86
Bpin
Me
Me
f1 (ppm)

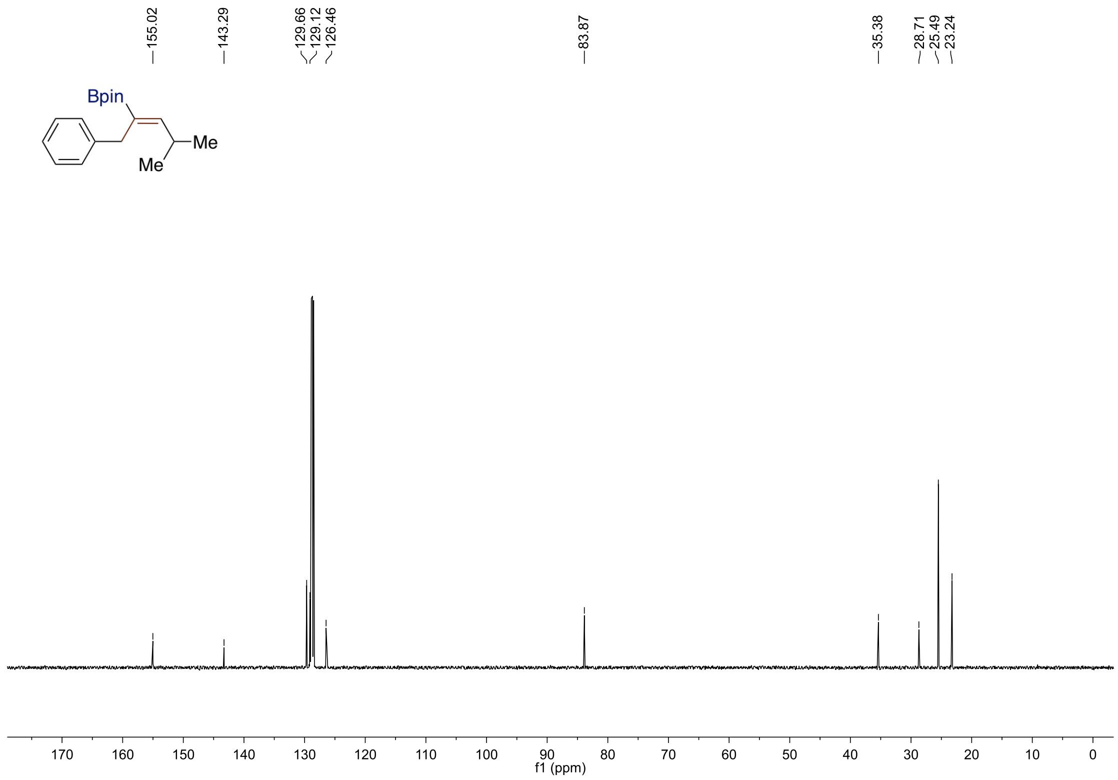
155.02
143.29
129.66
129.12
126.46
83.87
35.38
28.71
25.49
23.24
Bpin
Me
Me
f1 (ppm)

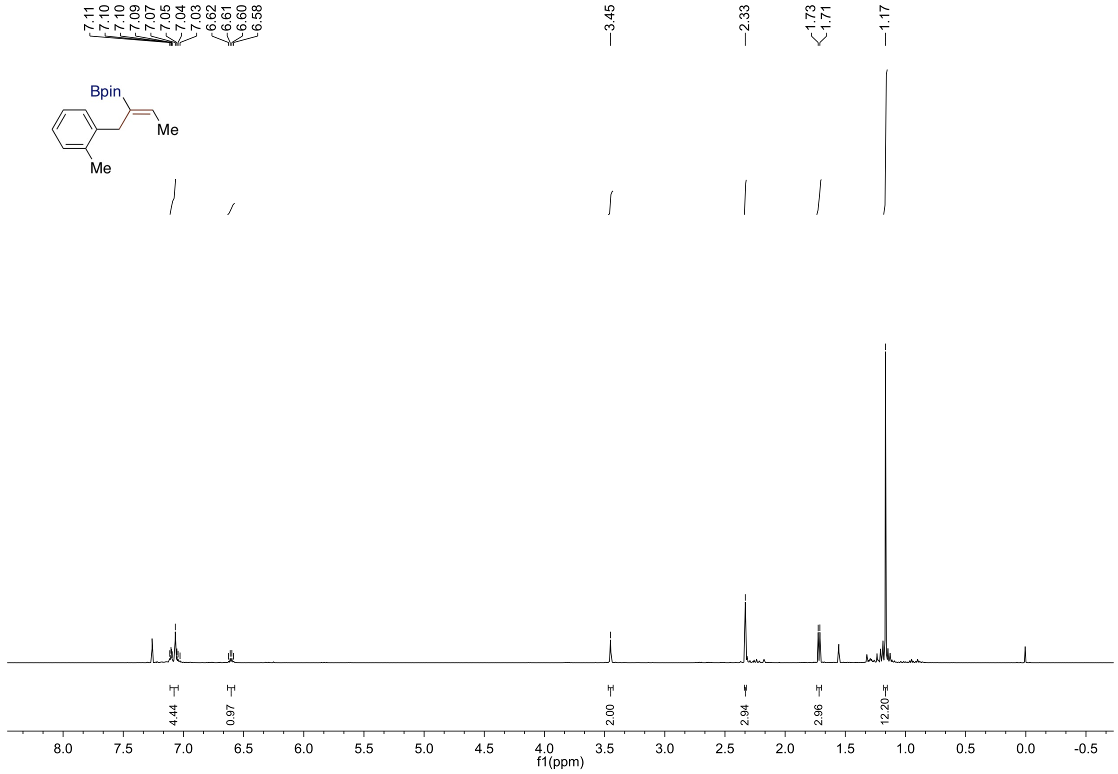
7.11
7.10
7.10
7.09
7.07
7.05
7.04
7.03
6.62
6.61
6.60
6.58
3.45
2.33
1.73
1.71
1.17
Bpin
Me
Me
4.44
0.97
2.00
2.94
2.96
12.20
f1(ppm)

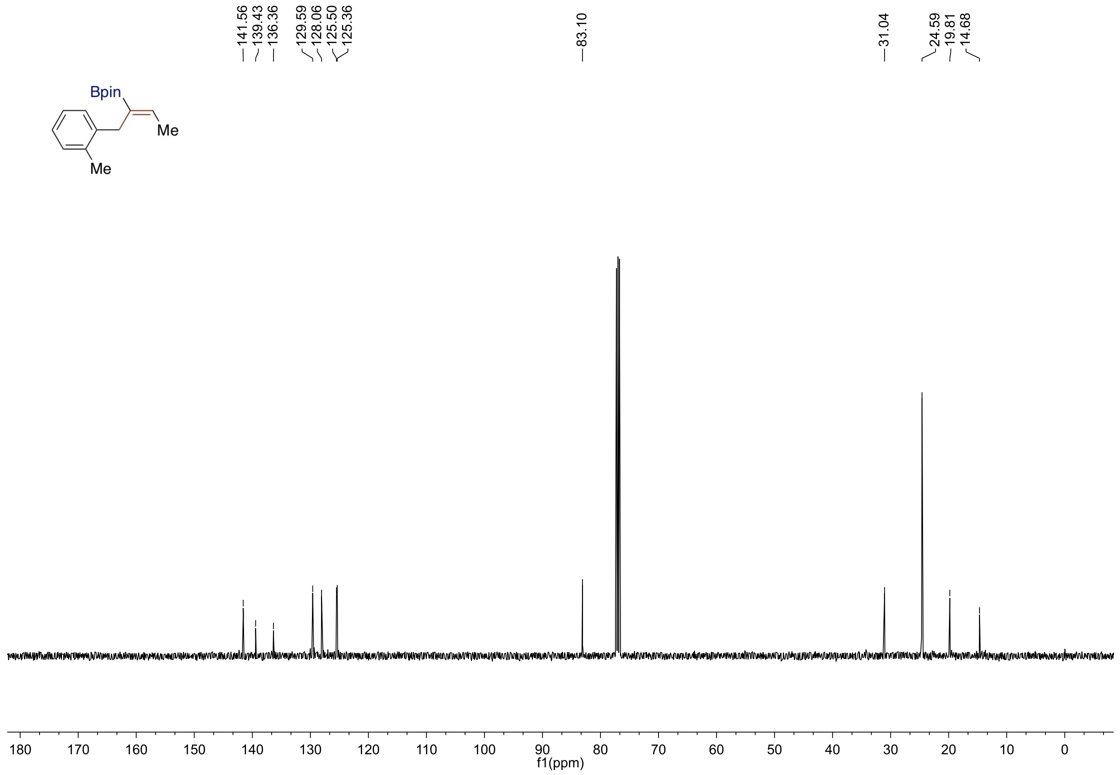
141.56
139.43
136.36
129.59
128.06
125.50
125.36
83.10
31.04
24.59
19.81
14.68
Bpin
Me
Me
f1(ppm)

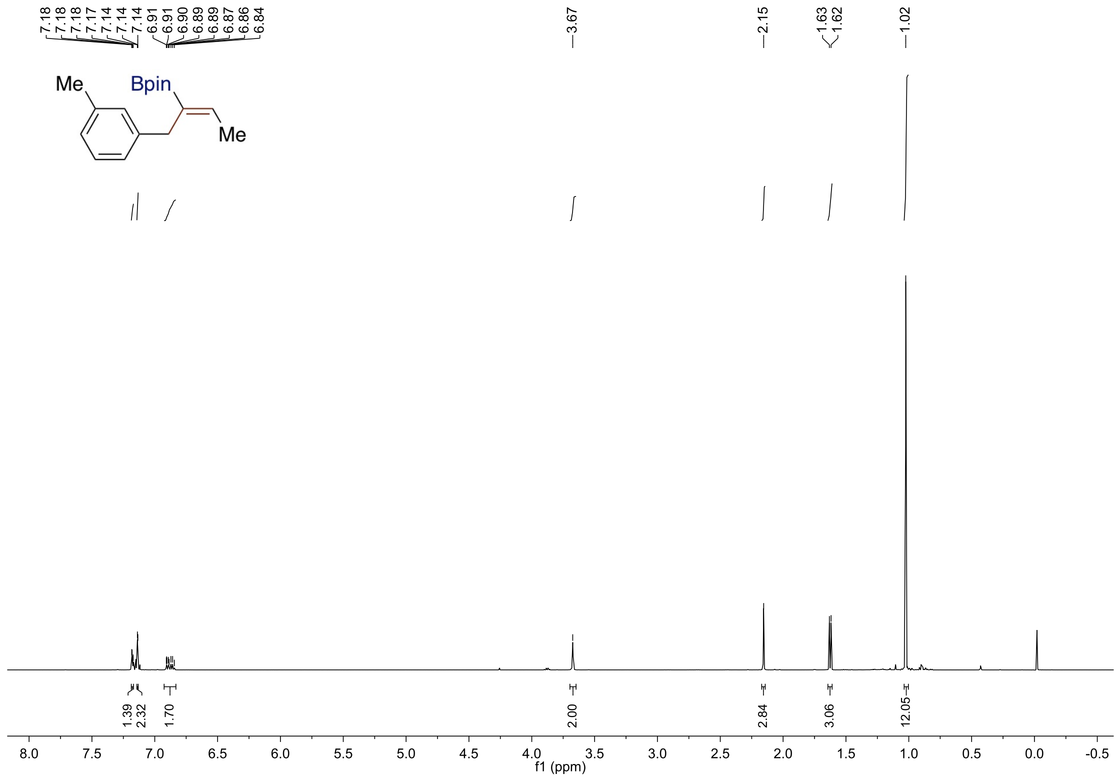
7.18
7.18
7.18
7.17
7.14
7.14
7.14
6.91
6.91
6.90
6.89
6.89
6.87
6.86
6.84
3.67
2.15
1.63
1.62
1.02
Me
Bpin
Me
1.39
2.32
1.70
2.00
2.84
3.06
12.05
f1 (ppm)

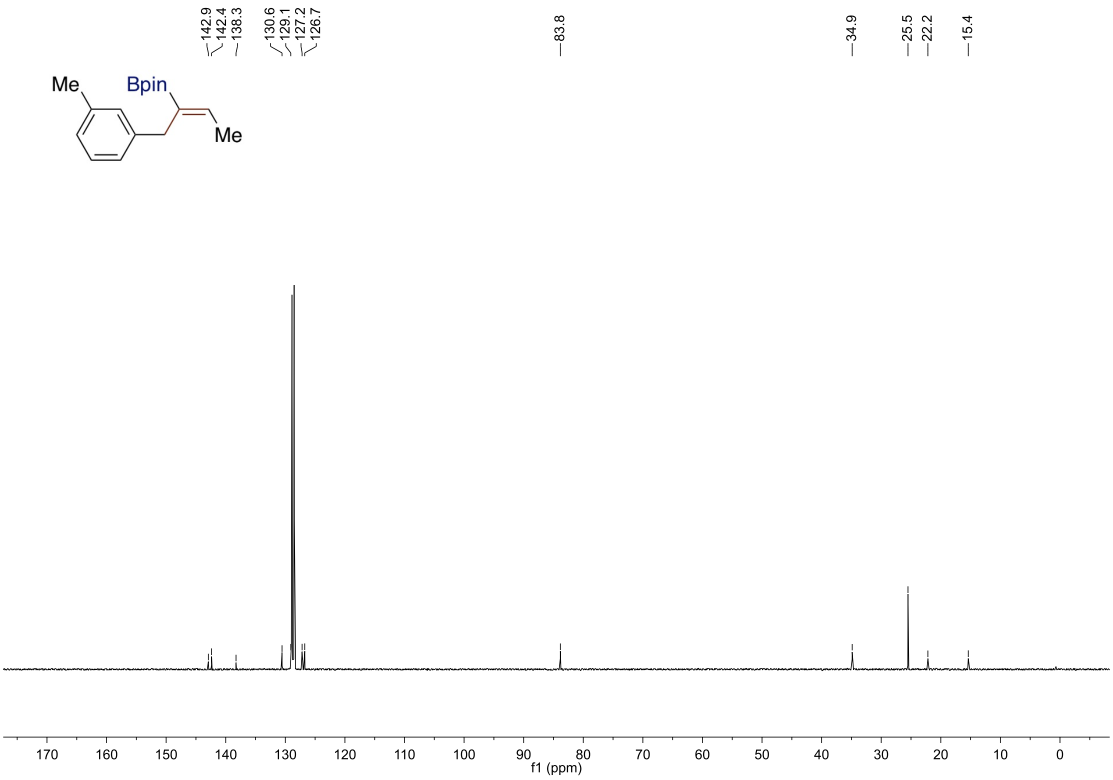

142.9
142.4
138.3
130.6
129.1
127.2
126.7
83.8
34.9
25.5
22.2
15.4
Me
Bpin
Me
170 160 150 140 130 120 110 100 90 80 70 60 50 40 30 20 10 0
f1 (ppm)


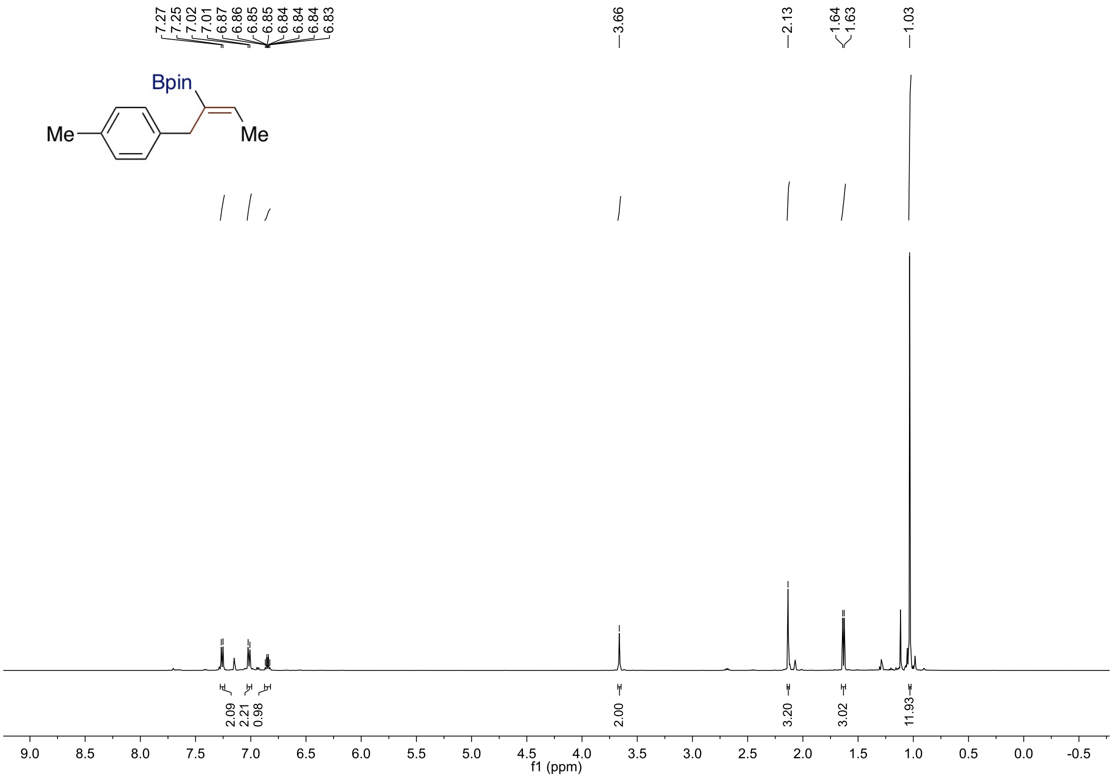

7.27
7.25
7.02
7.01
6.87
6.86
6.85
6.85
6.84
6.84
6.84
6.83
3.66
2.13
1.64
1.63
1.03
Bpin
Me
Me
2.09
2.21
0.98
2.00
3.20
3.02
11.93
9.0 8.5 8.0 7.5 7.0 6.5 6.0 5.5 5.0 4.5 4.0 3.5 3.0 2.5 2.0 1.5 1.0 0.5 0.0 -0.5
f1 (ppm)

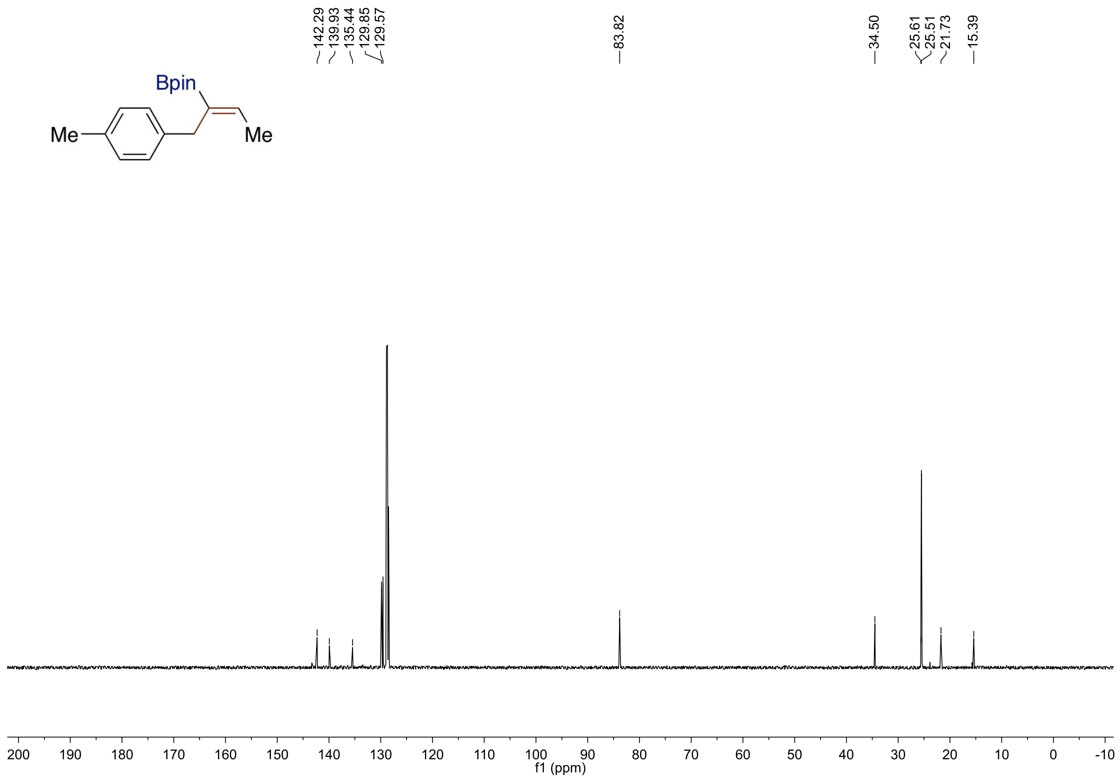

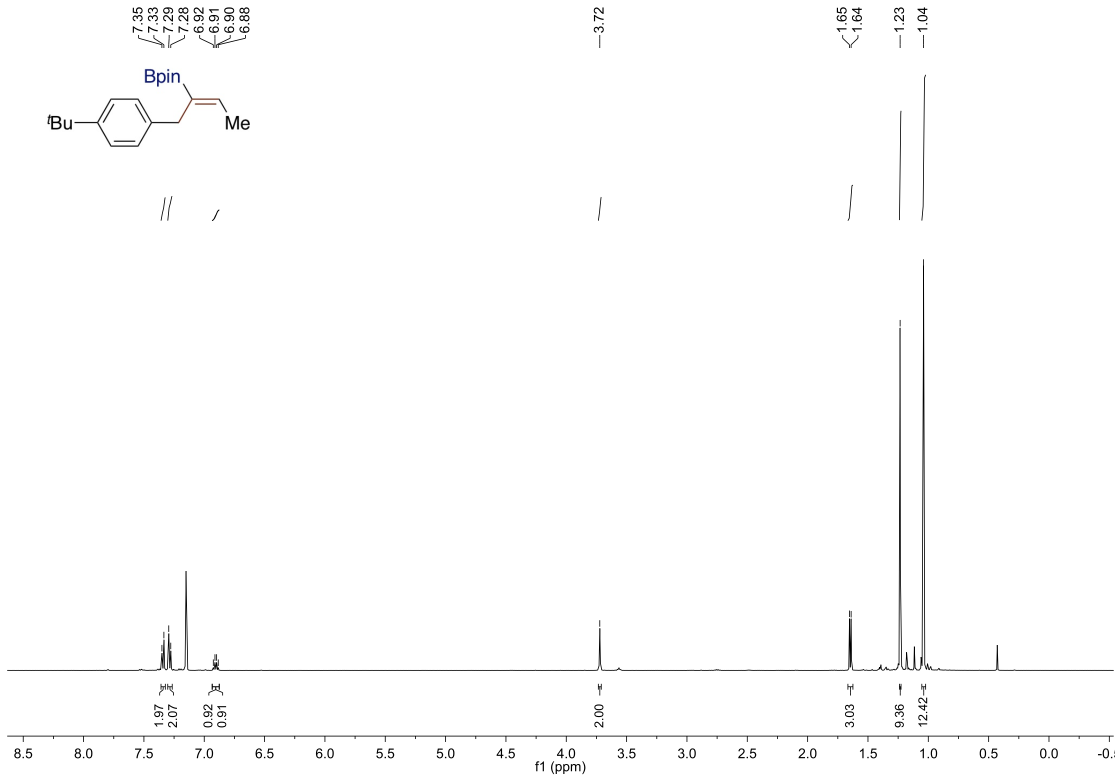

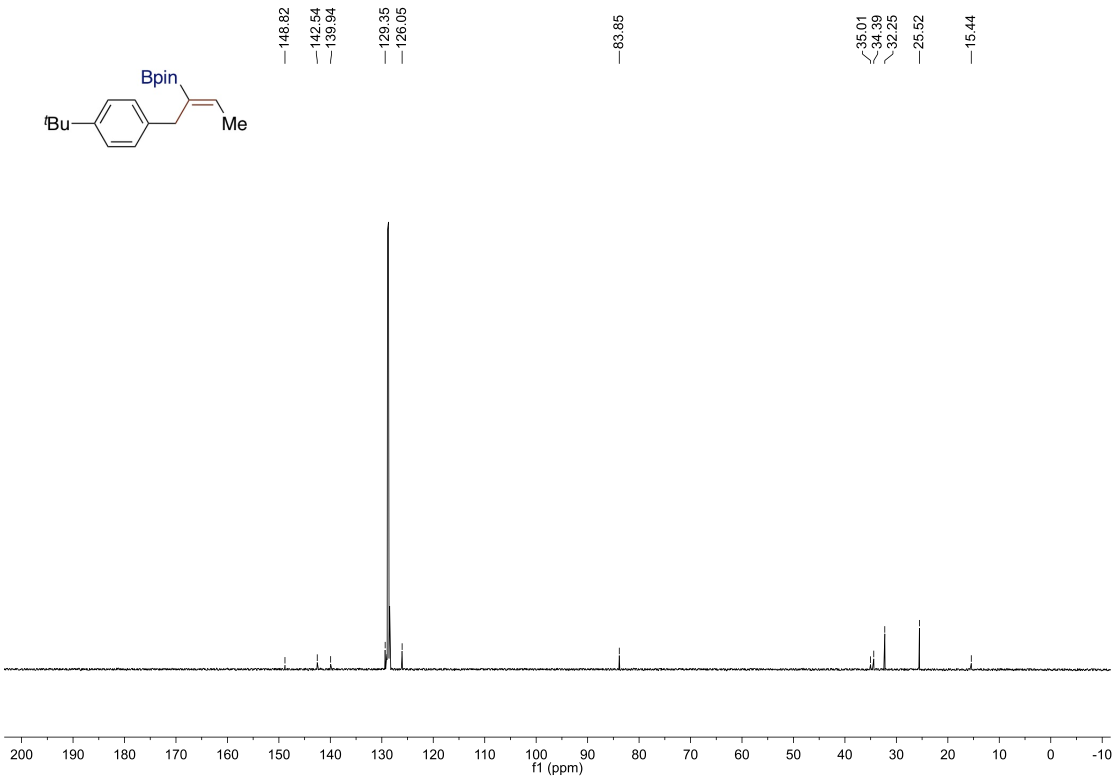
148.82
142.54
139.94
129.35
126.05
83.85
35.01
34.39
32.25
25.52
15.44
Bpin
tBu
Me
f1 (ppm)

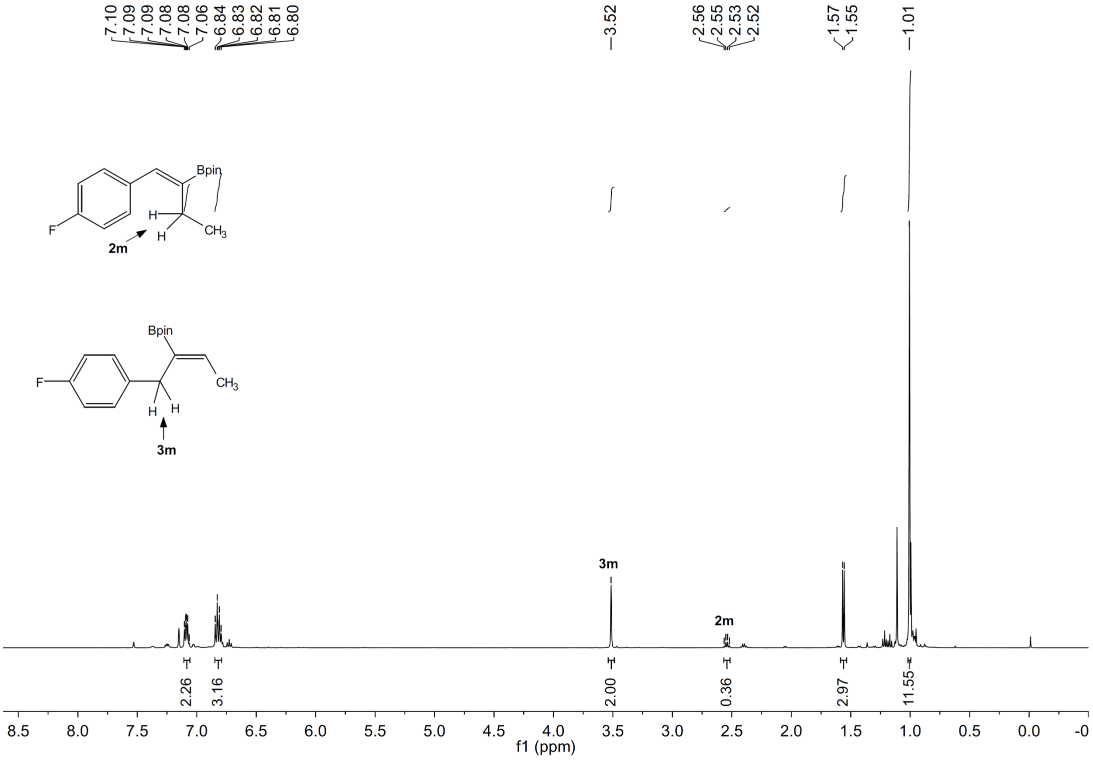
7.10
7.09
7.09
7.08
7.08
7.06
6.84
6.83
6.82
6.81
6.80
3.52
2.56
2.55
2.53
2.52
1.57
1.55
1.01
Bpin
F
H
H
CH3
2m
3m
2.26
3.16
2.00
0.36
2.97
11.55
f1 (ppm)

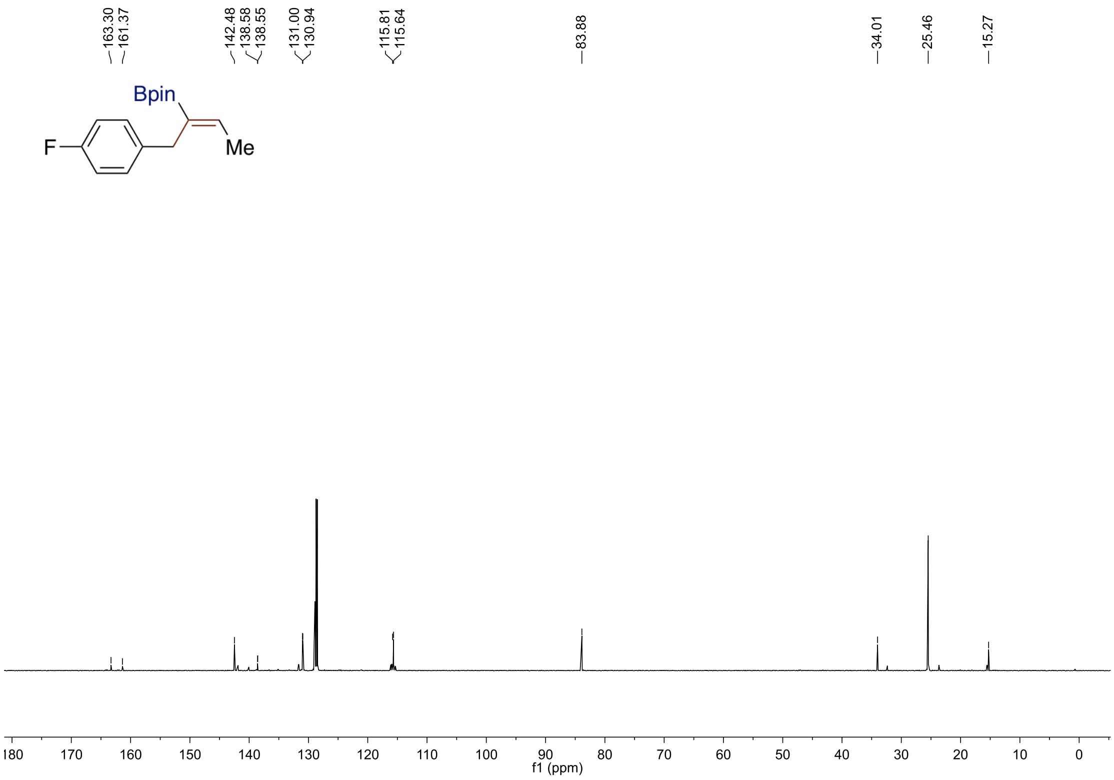
163.30
161.37
142.48
138.58
138.55
131.00
130.94
115.81
115.64
83.88
34.01
25.46
15.27
Bpin
F
Me
f1 (ppm)

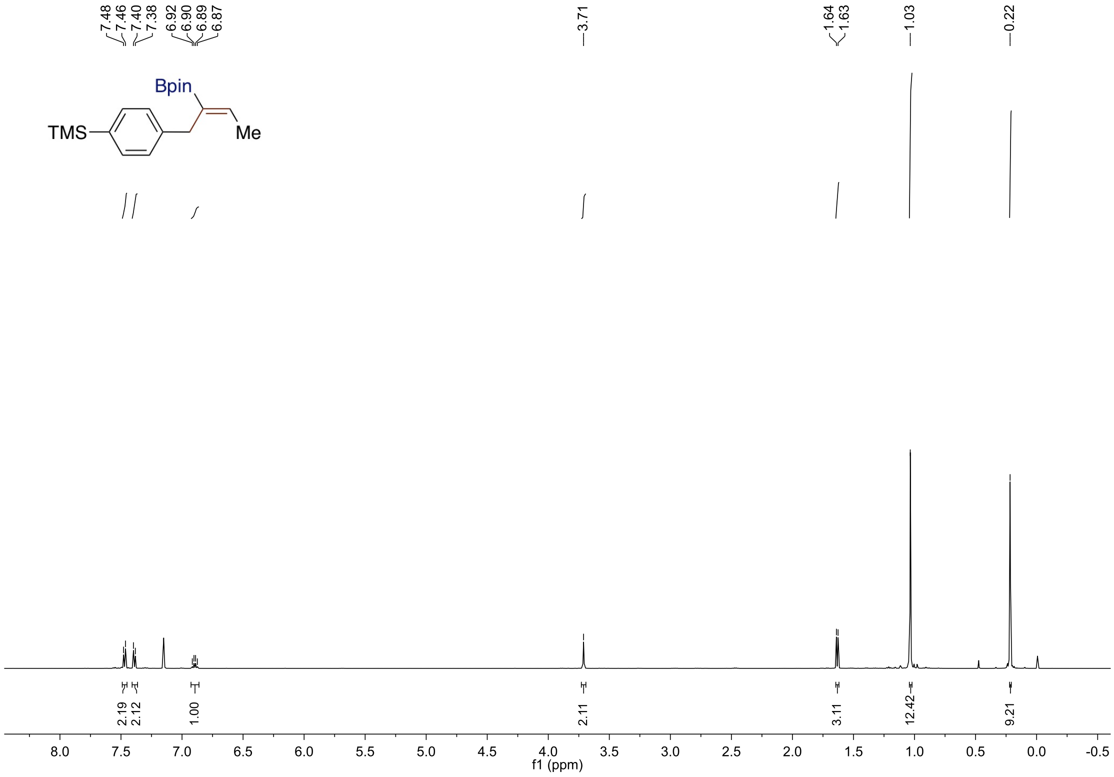
7.48
7.46
7.40
7.38
6.92
6.90
6.89
6.87
3.71
1.64
1.63
1.03
0.22
Bpin
TMS
Me
2.19
2.12
1.00
2.11
3.11
12.42
9.21
f1 (ppm)

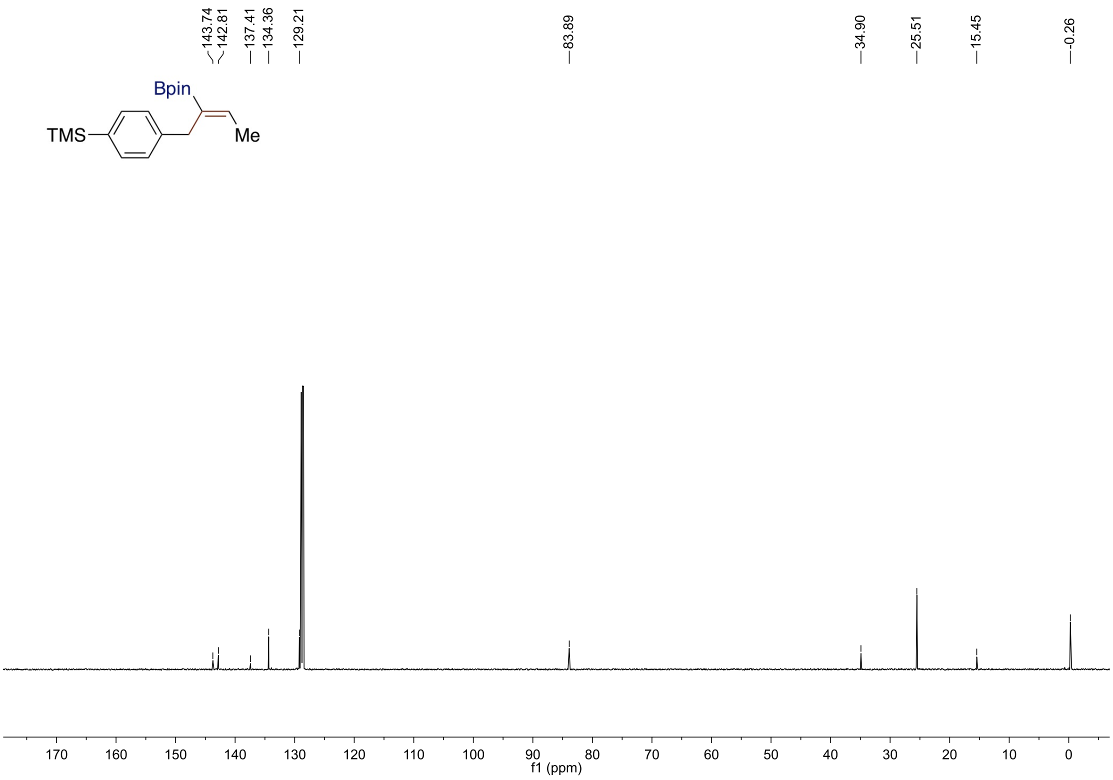
143.74
142.81
137.41
134.36
129.21
83.89
34.90
25.51
15.45
-0.26
Bpin
TMS
Me
170 160 150 140 130 120 110 100 90 80 70 60 50 40 30 20 10 0
f1 (ppm)

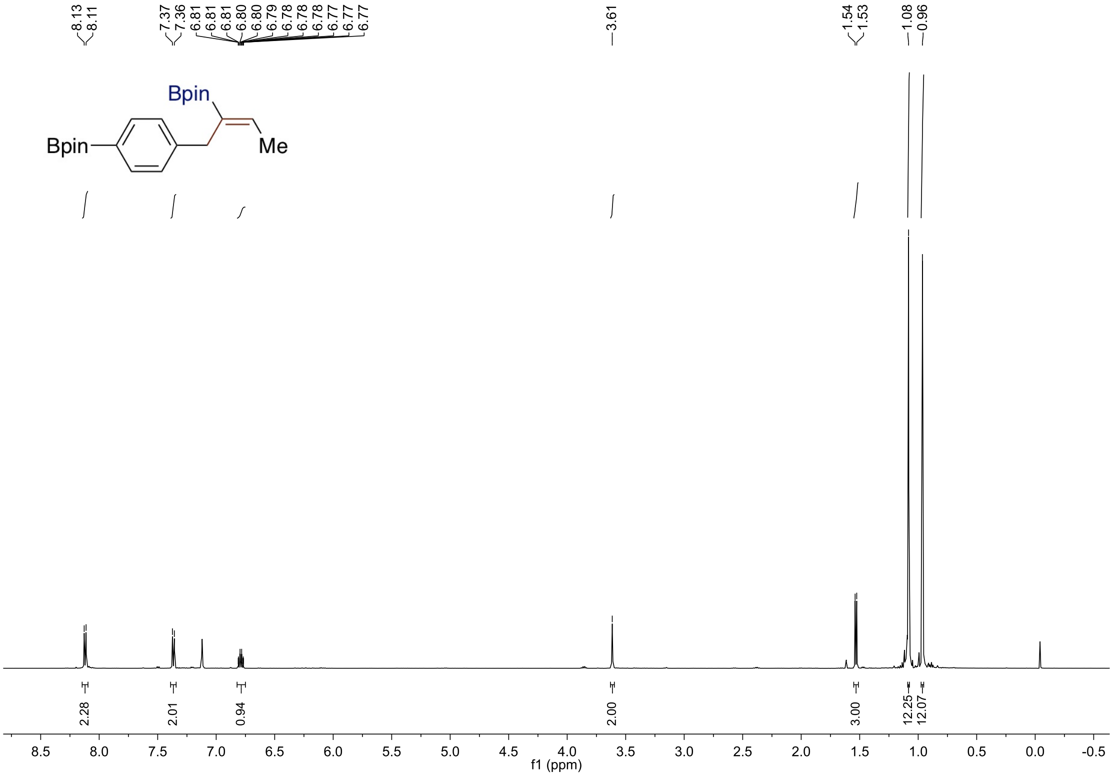
8.13
8.11
7.37
7.36
6.81
6.81
6.80
6.80
6.79
6.78
6.78
6.78
6.77
6.77
6.77
3.61
1.54
1.53
1.08
0.96
Bpin
Bpin
Me
2.28
2.01
0.94
2.00
3.00
12.25
12.07
8.5 8.0 7.5 7.0 6.5 6.0 5.5 5.0 4.5 4.0 3.5 3.0 2.5 2.0 1.5 1.0 0.5 0.0 -0.5
f1 (ppm)

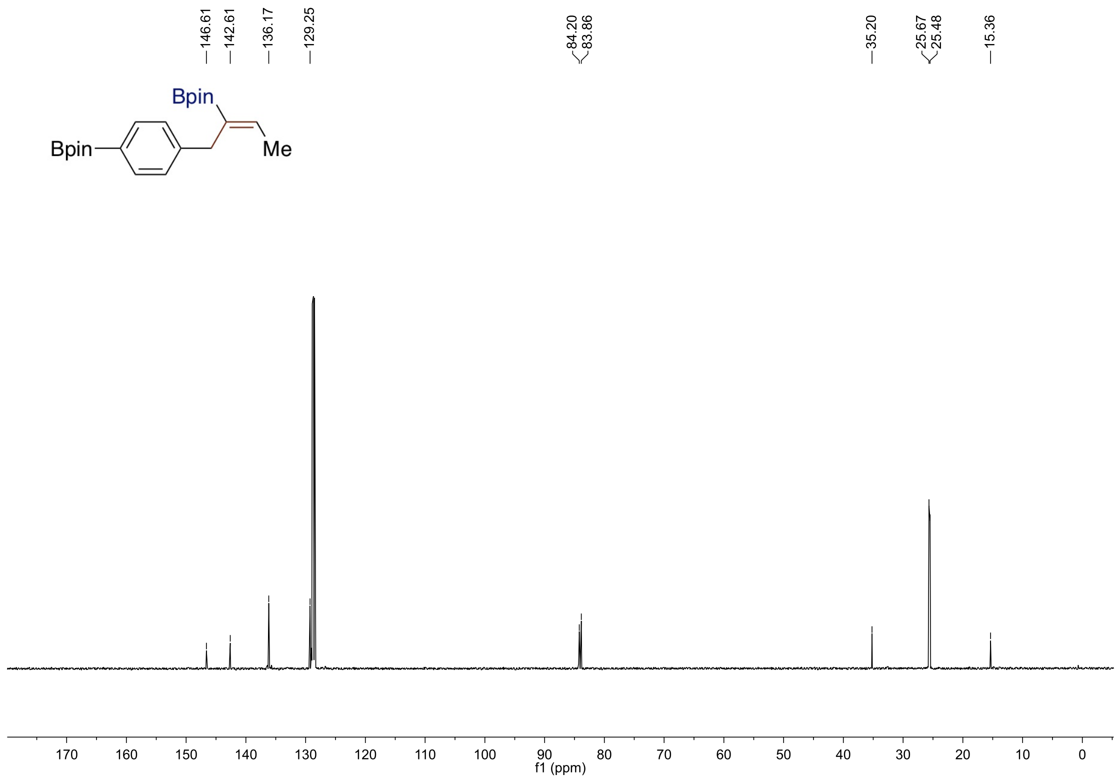

146.61
142.61
136.17
129.25
84.20
83.86
35.20
25.67
25.48
15.36
Bpin
Bpin
Me
170 160 150 140 130 120 110 100 90 80 70 60 50 40 30 20 10 0
f1 (ppm)


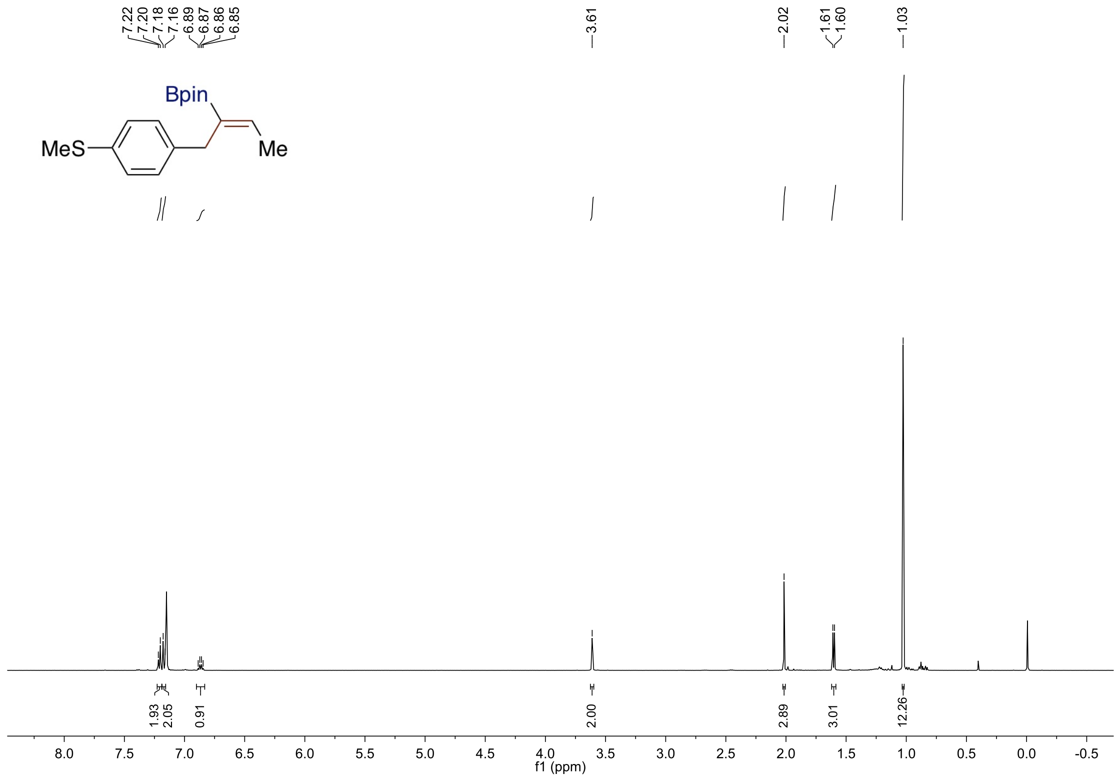

7.22
7.20
7.18
7.16
6.89
6.87
6.86
6.85
3.61
2.02
1.61
1.60
1.03
Bpin
MeS
Me
1.93
2.05
0.91
2.00
2.89
3.01
12.26
8.0 7.5 7.0 6.5 6.0 5.5 5.0 4.5 4.0 3.5 3.0 2.5 2.0 1.5 1.0 0.5 0.0 -0.5
f1 (ppm)

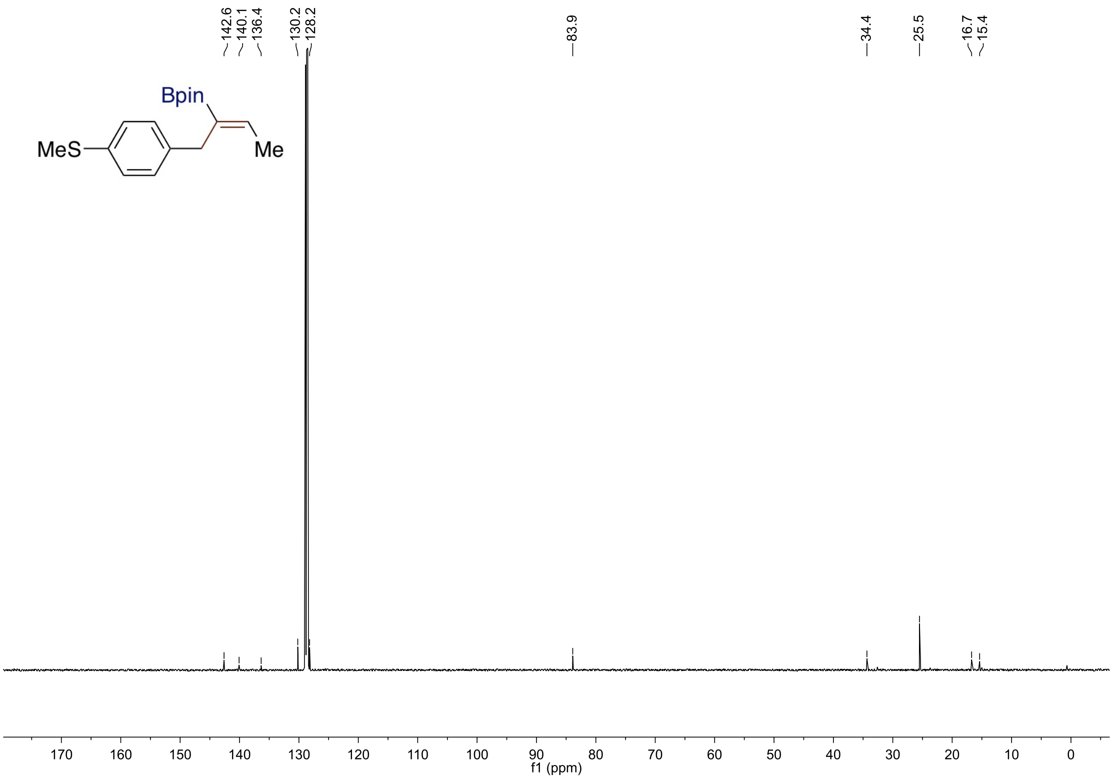
142.6
140.1
136.4
130.2
128.2
83.9
34.4
25.5
16.7
15.4
Bpin
MeS
Me
170 160 150 140 130 120 110 100 90 80 70 60 50 40 30 20 10 0
f1 (ppm)

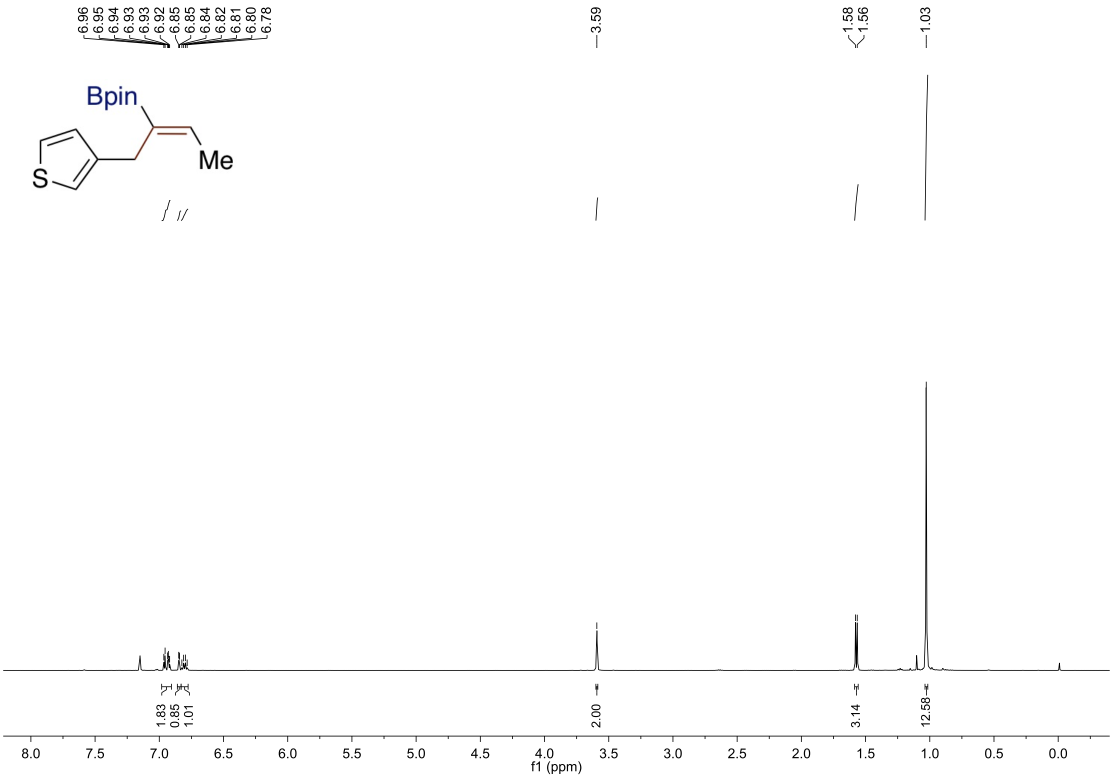
6.96
6.95
6.94
6.93
6.93
6.92
6.85
6.85
6.84
6.82
6.81
6.80
6.78
3.59
1.58
1.56
1.03
Bpin
S
Me
1.83
0.85
1.01
2.00
3.14
12.58
8.0 7.5 7.0 6.5 6.0 5.5 5.0 4.5 4.0 3.5 3.0 2.5 2.0 1.5 1.0 0.5 0.0
f1 (ppm)

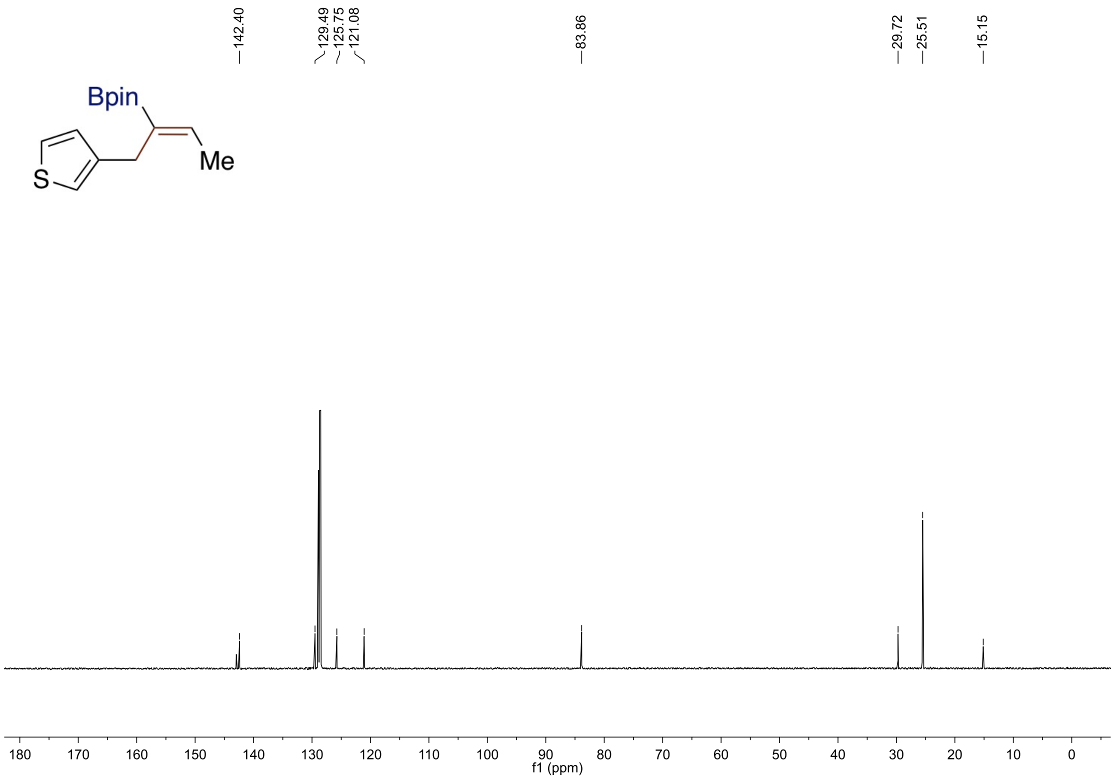
142.40
129.49
125.75
121.08
83.86
29.72
25.51
15.15
Bpin
S
Me
f1 (ppm)

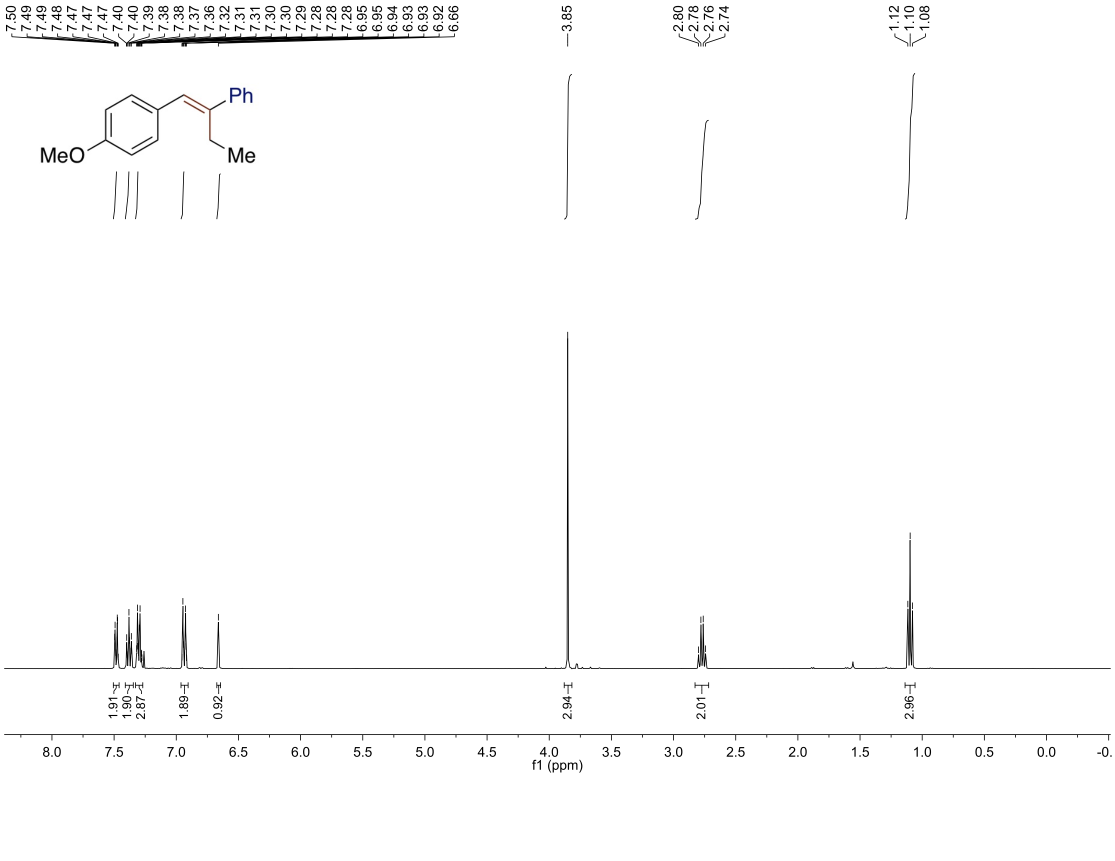
3.85
2.80
2.78
2.76
2.74
1.12
1.10
1.08
Ph
MeO
Me
1.91
1.90
2.87
1.89
0.92
2.94
2.01
2.96
f1 (ppm)

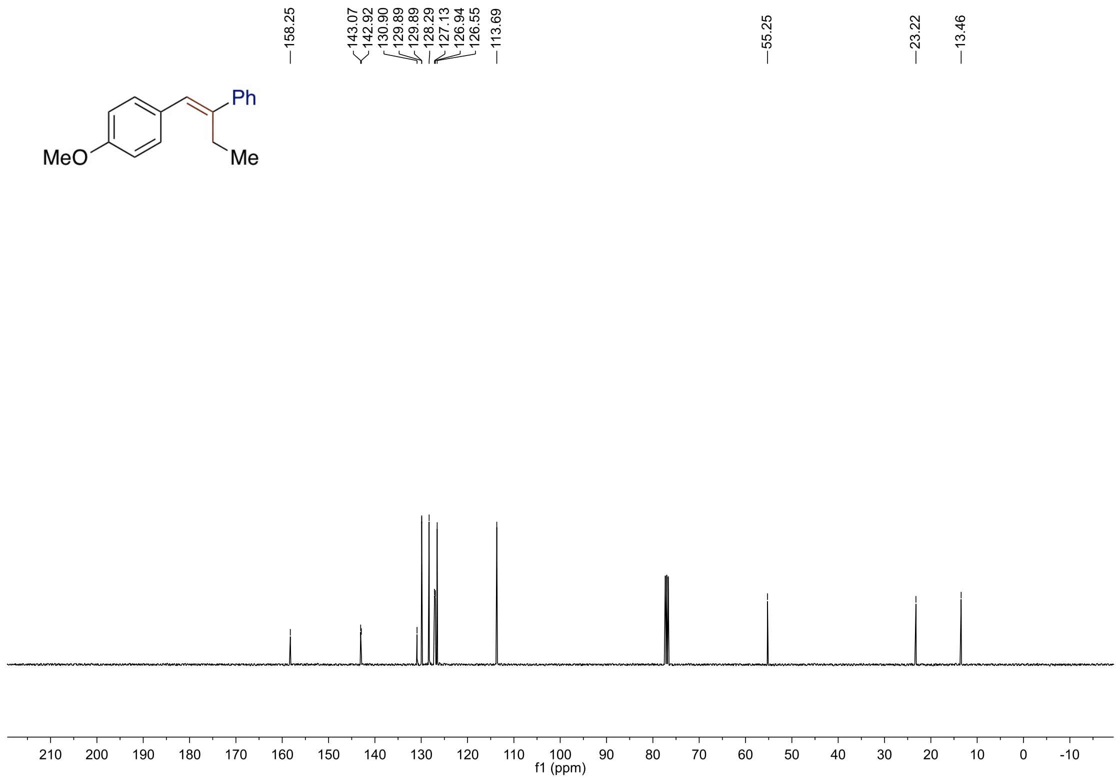

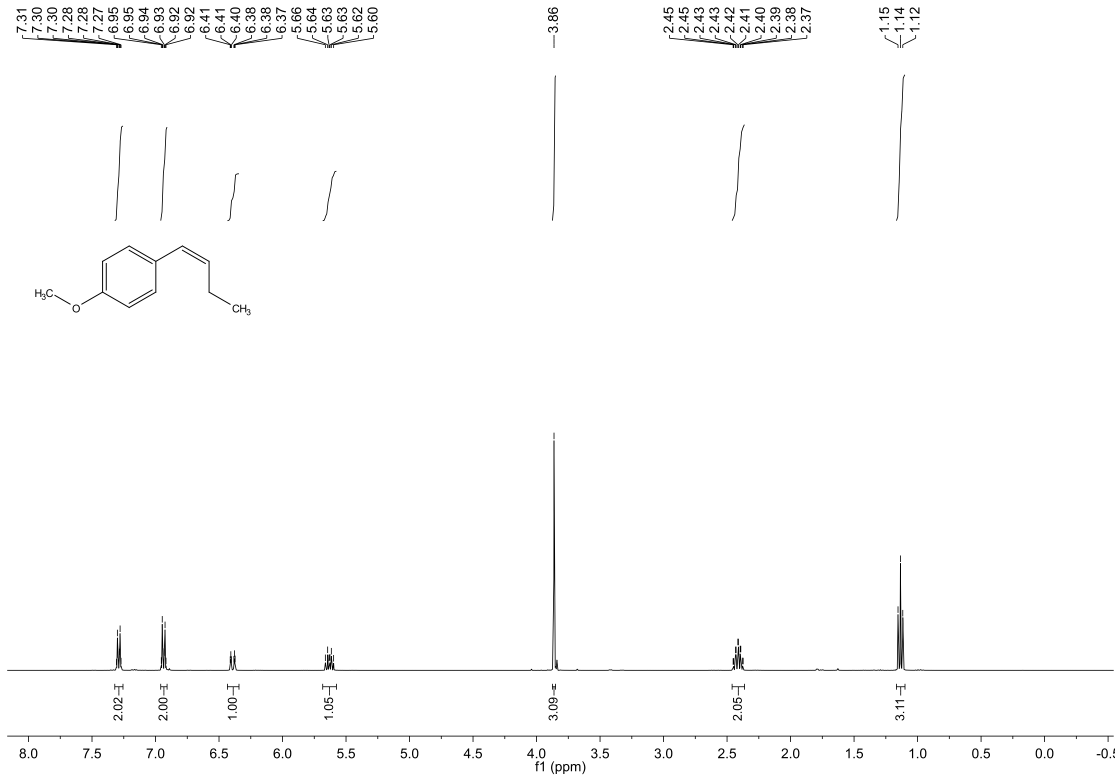

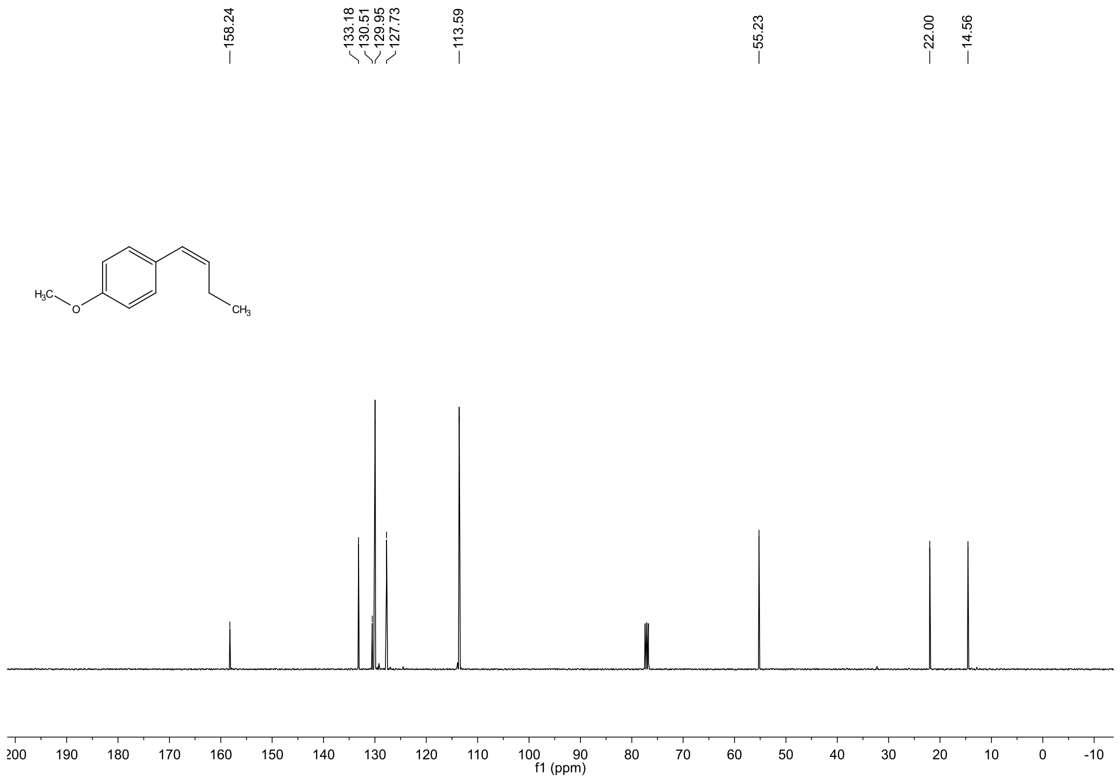
158.24
133.18
130.51
129.95
127.73
113.59
55.23
22.00
14.56
H3C
O
CH3
200 190 180 170 160 150 140 130 120 110 100 90 80 70 60 50 40 30 20 10 0 -10
f1 (ppm)

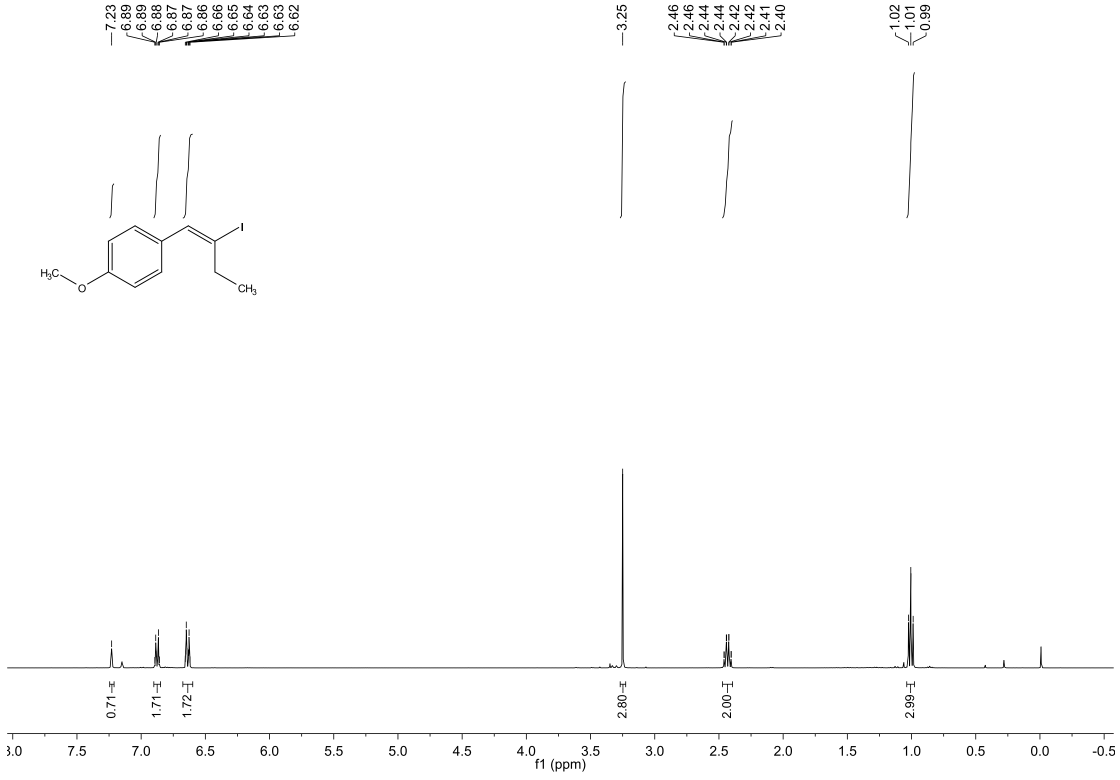
7.23
6.89
6.89
6.88
6.87
6.87
6.86
6.66
6.65
6.64
6.63
6.63
6.62
3.25
2.46
2.46
2.44
2.44
2.42
2.42
2.41
2.40
1.02
1.01
0.99
I
H3C
O
CH3
0.71
1.71
1.72
2.80
2.00
2.99
7.5 7.0 6.5 6.0 5.5 5.0 4.5 4.0 3.5 3.0 2.5 2.0 1.5 1.0 0.5 0.0 -0.5
f1 (ppm)

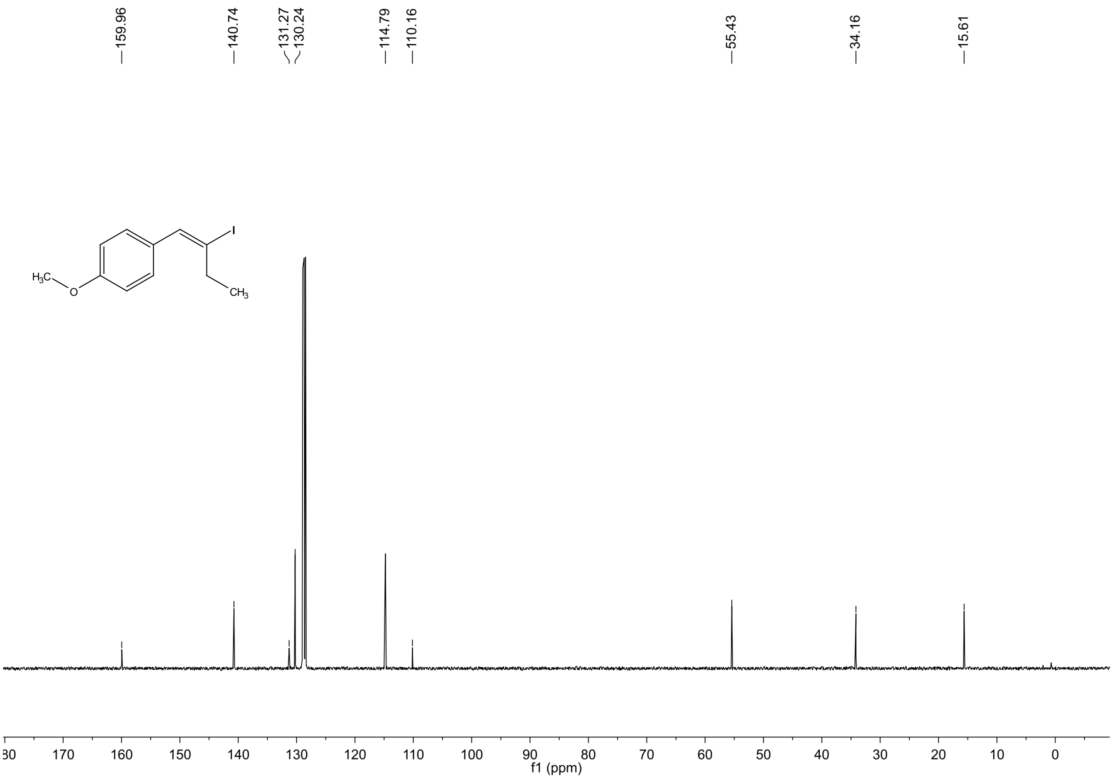

159.96
140.74
131.27
130.24
114.79
110.16
55.43
34.16
15.61
H3C
O
I
CH3
f1 (ppm)


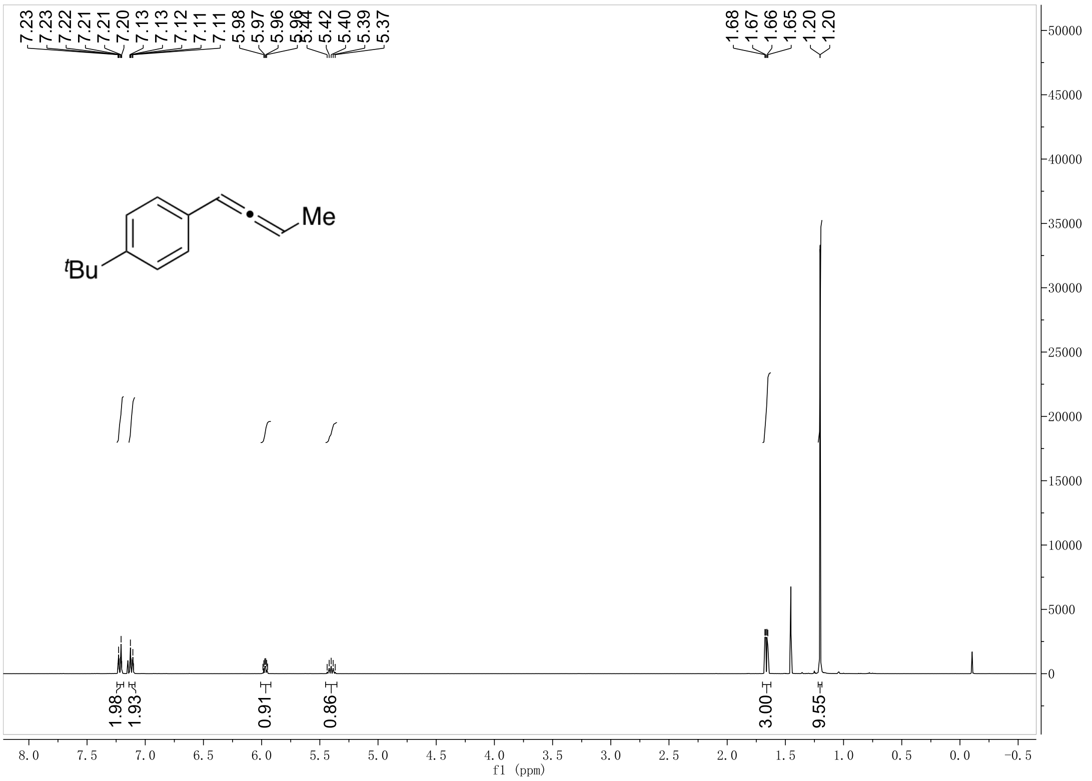

7.23
7.23
7.22
7.21
7.21
7.20
7.13
7.13
7.12
7.11
7.11
5.98
5.97
5.96
5.42
5.40
5.39
5.37
1.68
1.67
1.66
1.65
1.20
1.20
Me
tBu
1.98
1.93
0.91
0.86
3.00
9.55
f1 (ppm)

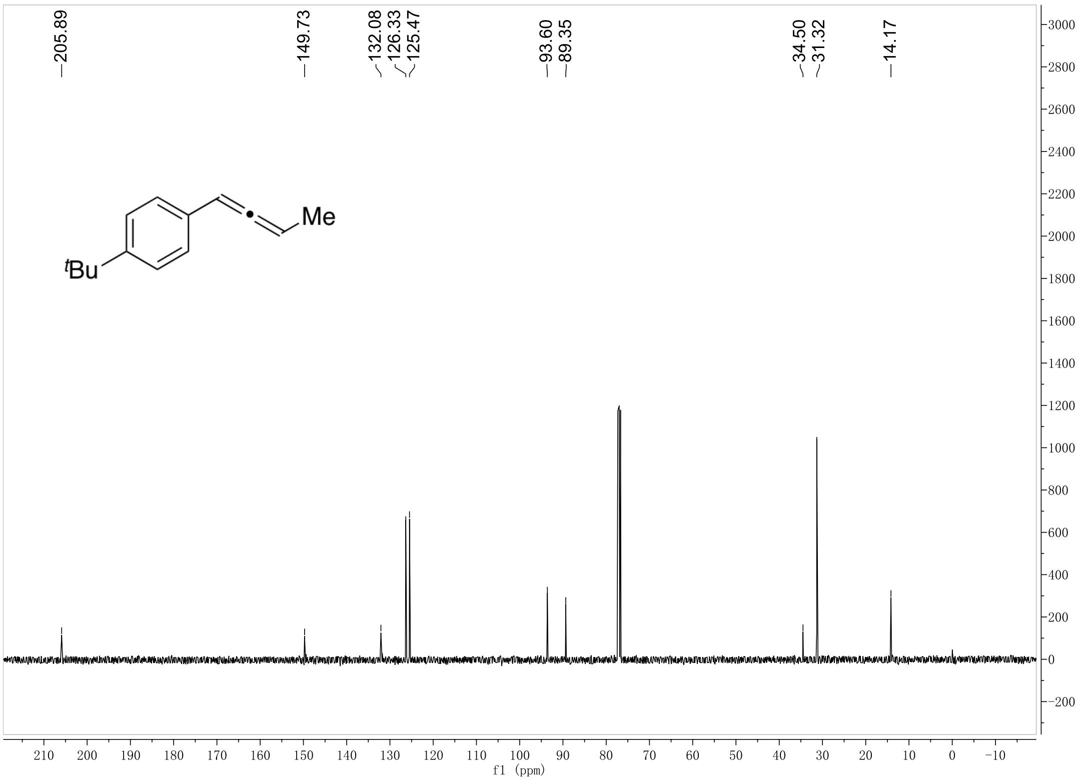
205.89
149.73
132.08
126.33
125.47
93.60
89.35
34.50
31.32
14.17
Me
tBu
f1 (ppm)

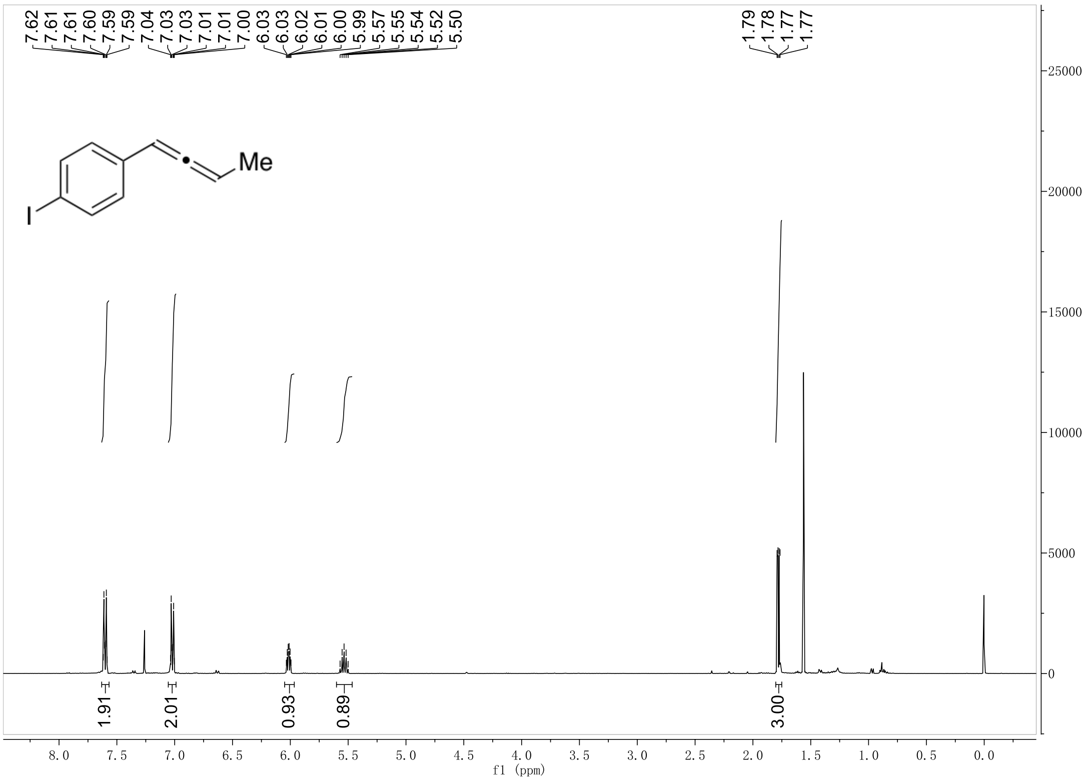
7.62
7.61
7.61
7.60
7.59
7.59
7.04
7.03
7.03
7.01
7.01
7.00
6.03
6.03
6.02
6.01
6.00
5.99
5.57
5.55
5.54
5.52
5.50
1.79
1.78
1.77
1.77
Me
I
1.91
2.01
0.93
0.89
3.00
f1 (ppm)

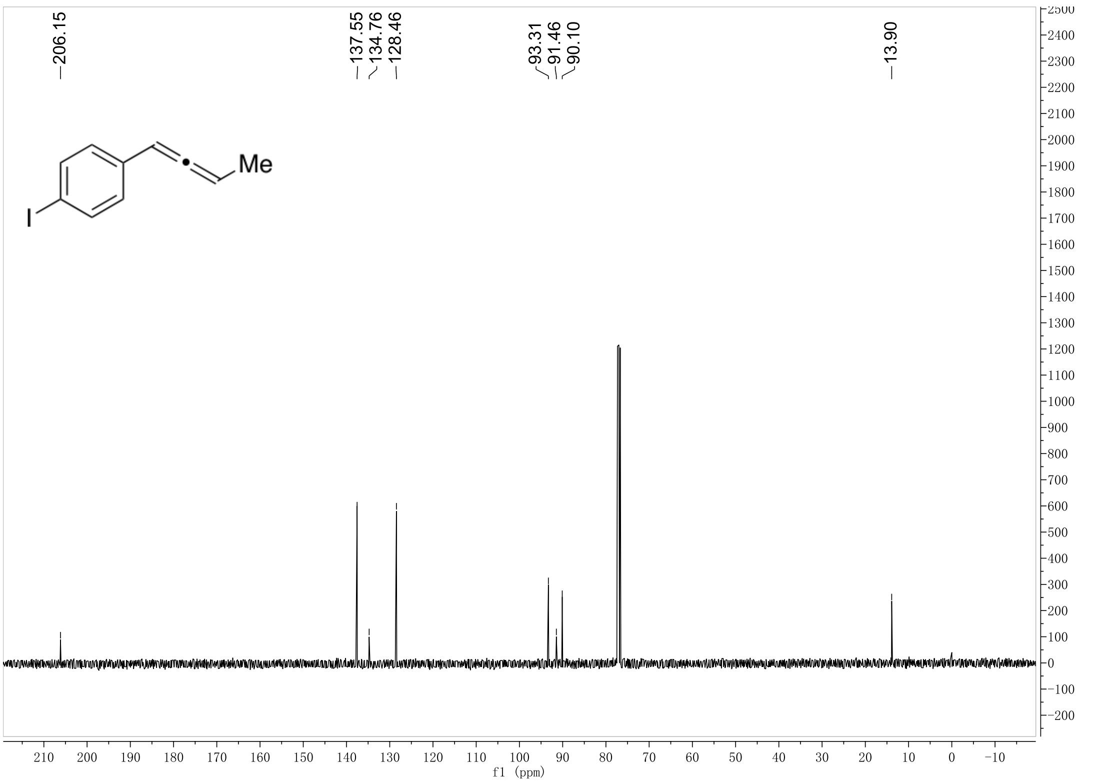
206.15
137.55
134.76
128.46
93.31
91.46
90.10
13.90
Me
I
f1 (ppm)

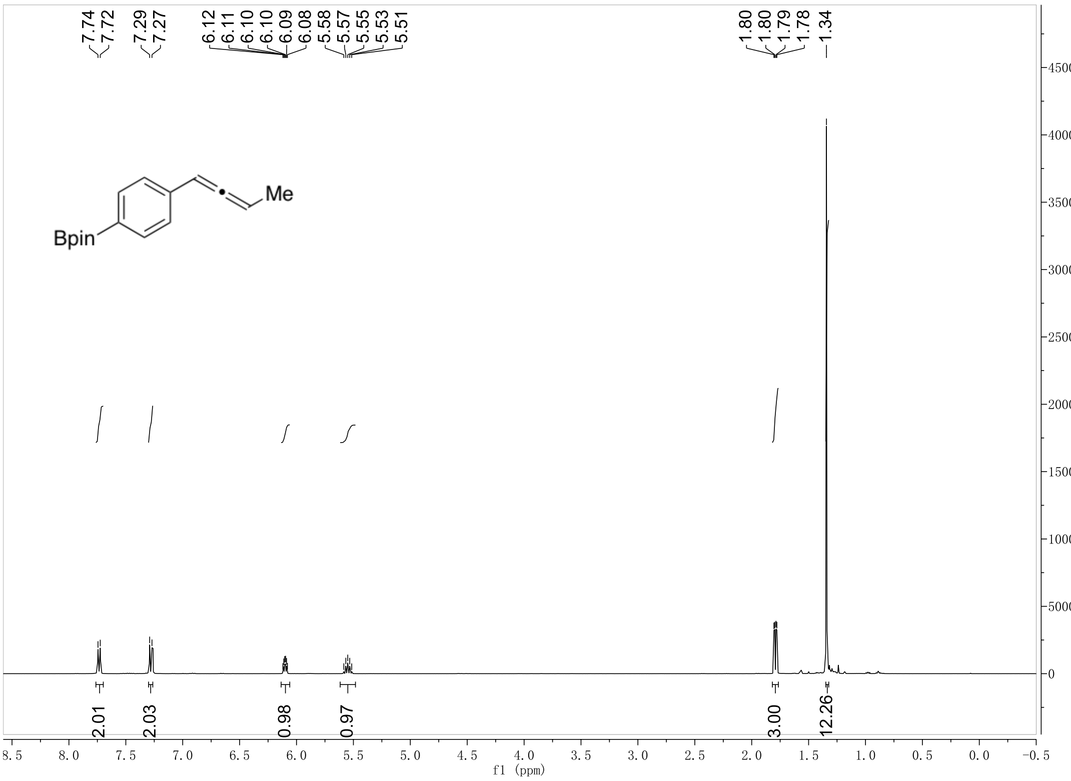
7.74
7.72
7.29
7.27
6.12
6.11
6.10
6.10
6.09
6.08
5.58
5.57
5.55
5.53
5.51
1.80
1.80
1.79
1.78
1.34
Me
Bpin
2.01
2.03
0.98
0.97
3.00
12.26
f1 (ppm)

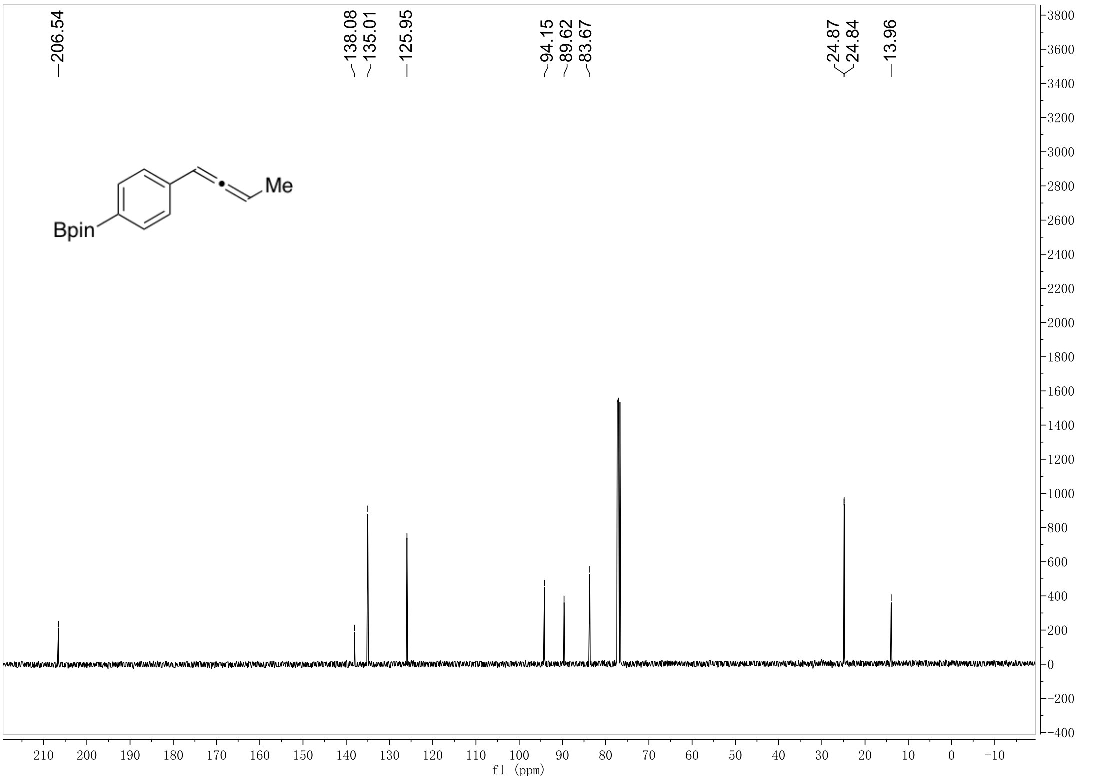
206.54
138.08
135.01
125.95
94.15
89.62
83.67
24.87
24.84
13.96
Bpin
Me
f1 (ppm)

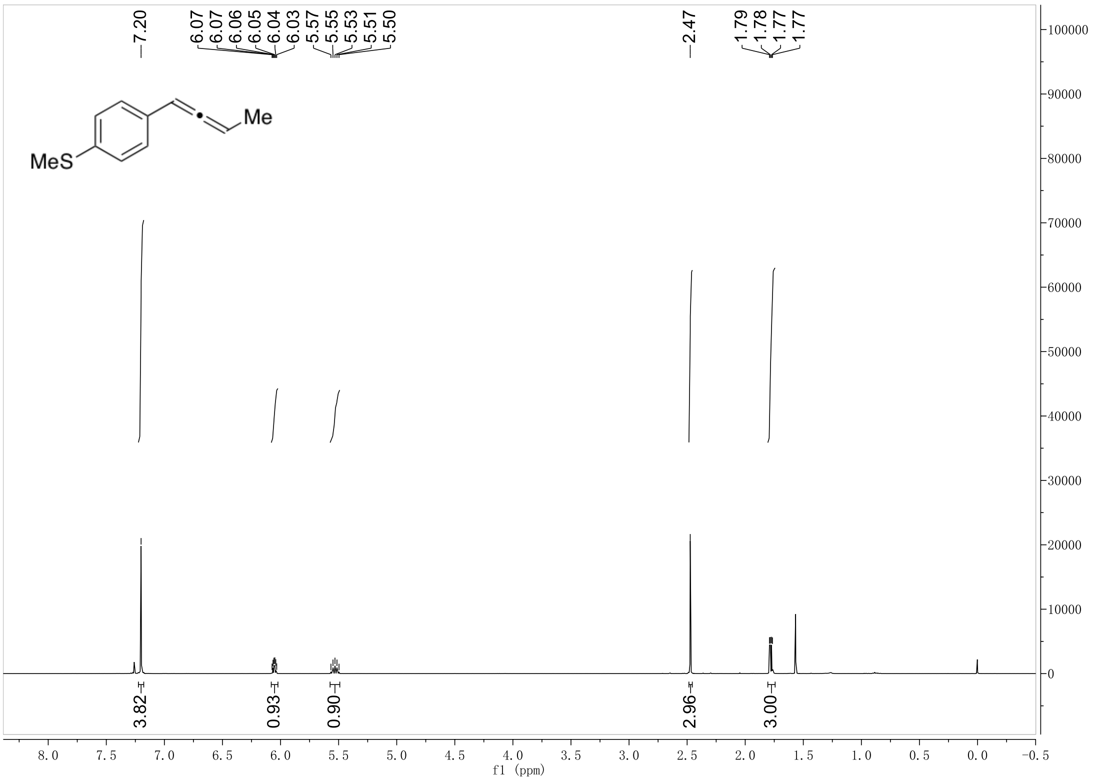
7.20
6.07
6.07
6.06
6.05
6.04
6.03
5.57
5.55
5.53
5.51
5.50
2.47
1.79
1.78
1.77
1.77
MeS
Me
3.82
0.93
0.90
2.96
3.00
f1 (ppm)

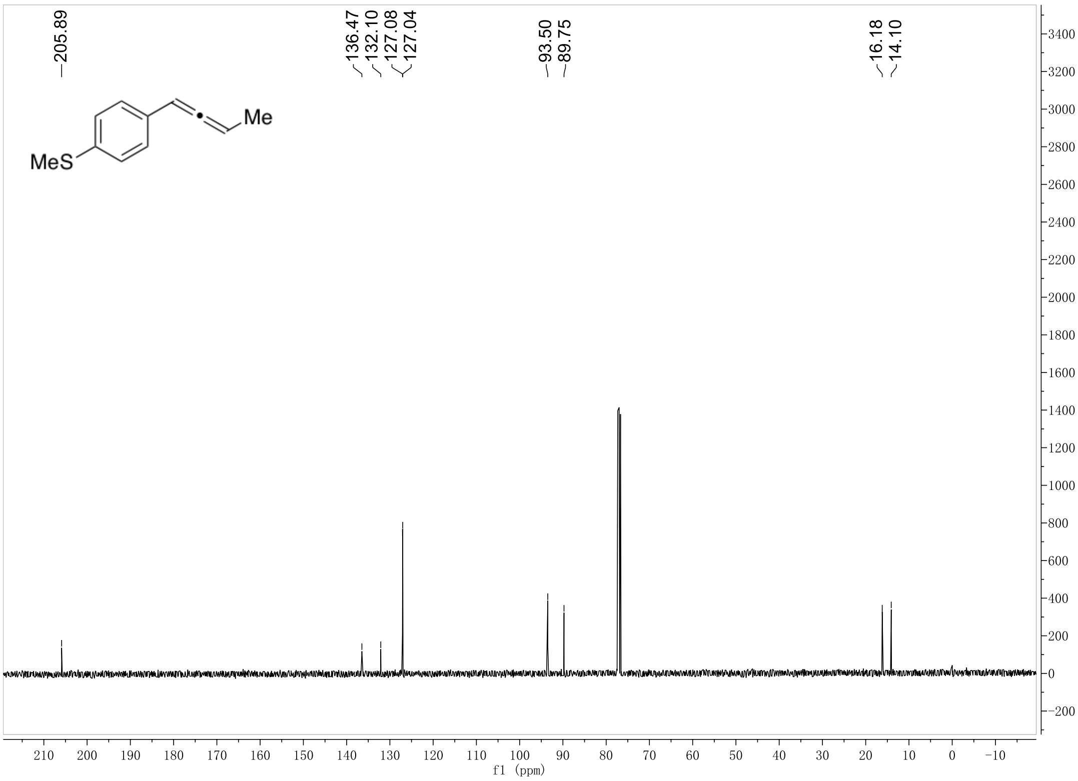
205.89
136.47
132.10
127.08
127.04
93.50
89.75
16.18
14.10
Me
MeS
f1 (ppm)
